# Programmed Aggregation of Lipidated Nitrobenzoselenadiazole as a Photo‐Activatable Pyroptosis Inducer

**DOI:** 10.1002/adhm.202501567

**Published:** 2025-08-05

**Authors:** Jong Min An, Hyunyoung Choi, Hyo In Kim, Do‐Yeon Kim, Jahyun Kim, Jinbong Park, Junyang Jung, Na Young Jeong, Dokyoung Kim

**Affiliations:** ^1^ College of Medicine Kyung Hee University Seoul 02447 Republic of Korea; ^2^ Department of Science in Korean Medicine Graduate School Kyung Hee University Seoul 02447 Republic of Korea; ^3^ Medical Research Center for Bioreaction to Reactive Oxygen Species and Biomedical Science Institute Core Research Institute (CRI) Kyung Hee University Seoul 02447 Republic of Korea; ^4^ Department of Biomedical Science Graduate School Kyung Hee University Seoul 02447 Republic of Korea; ^5^ Department of Precision Medicine Graduate School Kyung Hee University Seoul 02447 Republic of Korea; ^6^ Department of Anatomy and Neurobiology College of Medicine Kyung Hee University Seoul 02447 Republic of Korea; ^7^ Department of Anatomy and Cell Biology College of Medicine Dong‐A University Busan 49201 Republic of Korea; ^8^ KHU‐KIST Department of Converging Science and Technology Kyung Hee University Seoul 02447 Republic of Korea; ^9^ UC San Diego Materials Research Science and Engineering Center (UCSD MRSEC) La Jolla California 92093 USA

**Keywords:** aggregation, lipidation, photodynamic therapy, photosensitizer, pyroptosis

## Abstract

Bioactive photosensitizers in photodynamic therapy (PDT) have emerged as a promising therapeutic approach for tumor treatment. However, the aggregation of photosensitizers in aqueous solutions could hinder their efficacy, leading to reduced generation of reactive oxygen species (ROS) in biological systems and lower therapeutic effectiveness. For the first time, this work discloses a programmed aggregation system based on the nitrobenzoselenadiazole (NBSD) scaffold with varying alkyl chain lengths (C1, C3, and C8), focusing on their potential as photo‐activable pyroptosis inducers. This study underscores the significance of molecular design in developing effective photosensitizers and marks a new era in controlling molecular packing and photophysical properties. Among the candidates, NBSD‐NOc exhibits superior performances in several areas: (i) aggregation‐enhanced PDT effect, (ii) high cellular uptake, (iii) induction of programmed cell death, (iv) implantable properties, and (v) high biocompatibility. Overall, this work highlights the critical balance between aggregation patterns and photophysical properties, presenting a promising strategy for post‐surgical management using implantable photosensitizers to address the potential challenge of tumor recurrence.

## Graphical abstract

This study presents alkylated nitrobenzoselenadiazole (NBSD) photosensitizers that self‐assemble into programmable aggregates, enabling photo‐triggered pyroptosis and enhanced ROS generation. The lead compound, NBSD‐NOc, exhibits superior photodynamic activity, high cellular uptake, and implantable gel compatibility with pH‐responsive release. This strategy offers a promising therapeutic approach for preventing tumor recurrence following surgical resection through localized pyroptosis‐driven cell death.

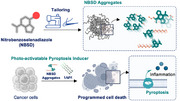

## 1 Introduction

Bioactive photosensitizers (PS) are light‐absorbing molecules that, upon excitation, release their absorbed energy in the form of a triplet state (TS), transitioning to a stable form in biological systems.^[^ ^1^ ^]^ The released energy generates reactive oxygen species (ROS), which are highly unstable and can cause damage to DNA, RNA, and proteins.^[^ ^2^, ^3^, ^4^ ^]^ These oxidative effects have attracted attention in the pharmaceutical field, particularly for cancer treatment, leading to the development of photodynamic therapy (PDT). Since the discovery of the first PS, hematoporphyrin in the 1960s, many researchers have reported various types of PS.^[^ ^5^ ^]^ Notably, porphyrin‐based PSs and others (such as acridine orange, mTHPC, and 5‐ALA) have been approved by the Food and Drug Administration (FDA) for tumor treatment.^[^ ^6^, ^7^, ^8^ ^]^ However, PSs in aqueous solutions (e.g., phosphate buffer, plasma, and other biological solutions) often experience lower fluorescence emission yields and aggregation‐induced quenching (AIQ).^[^ ^9^ ^]^ According to Jablonski's diagram, aggregation affects both the planar architecture and stacking interactions of PSs, disrupting charge transfer and leading to decreased ROS generation and fluorescence emission.^[^ ^10^, ^11^, ^12^ ^]^ This aggregation phenomenon can result in a significant loss of both ROS production and fluorescence.

Recent reports suggest that charge transfer within molecular structures can vary depending on the aggregate pattern.^[^ ^13^ ^]^ When small‐molecule fluorophores and PSs assemble in the solid state, they can often form either H‐type or J‐type aggregates, which are determined by the relative alignment of transition dipole moments between adjacent molecules.^[^ ^13^, ^14^ ^]^ In H‐aggregates, the molecules predominantly stack in a face‐to‐face configuration, while J‐aggregates are characterized by a head‐to‐tail stacking arrangement.^[^ ^15^ ^]^ These distinct structural features can alter charge transfer, resulting in increased intersystem crossing (ISC) and ROS generation. Based on this, several research groups have sought to overcome the limitations of AIQ in biological applications through aggregate pattern regulation.

In biological systems, lipidation occurs in various eukaryotic proteins and regulates numerous biological pathways by modulating hydrophobicity.^[^ ^16^ ^]^ This process has been applied to the modification of drugs and imaging agents to enhance their hydrophobicity. Recent studies have also suggested that lipidation of PSs can affect ROS generation, as observed in the photosensitization of long‐tailed BODIPY, alkylated PSs, and lipophilic decyl chain‐pterin conjugates.^[^ ^17^, ^18^, ^19^ ^]^ Based on this, we hypothesized that the lipidation of PSs could influence aggregate‐patterning features, affecting ROS generation, and mitigate the AIQ effect of PSs in biological systems.

**Figure 1:**
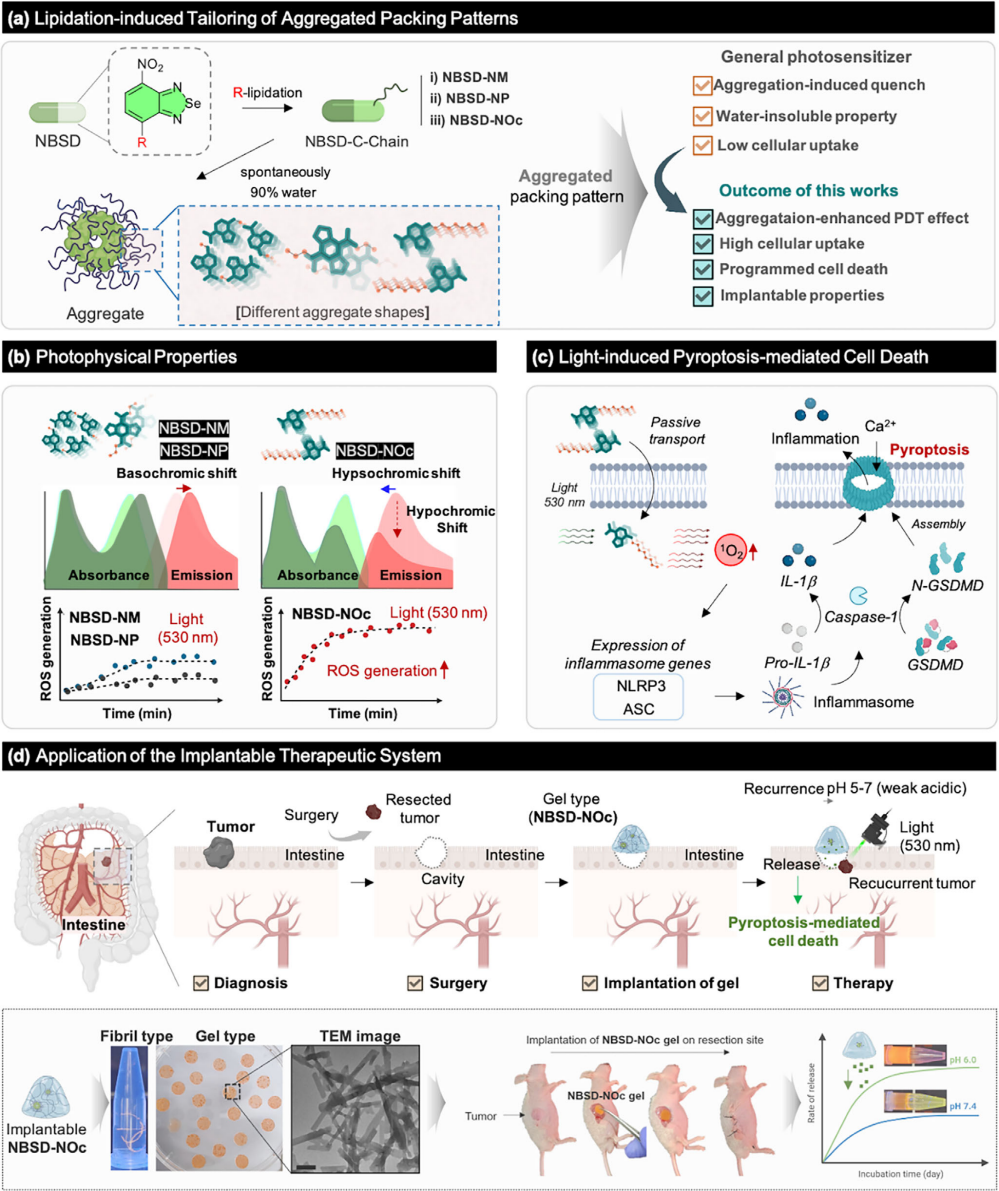
Schematic illustration of the strategy and research outline for this work:102 a) Lipidation‐induced tailoring of aggregated packing patterns to enhance photodynamic therapy (PDT) performance. b) Photophysical properties are caused by different aggregation patterns. c) Photoactivated pyroptosis‐mediated cell death. d) Application of the implantable therapeutic system.

For the first time, we disclosed the biological effects of programmed aggregation based on the alkylation of nitrobenzoselenadiazole (NBSD) as a photo‐activable pyroptosis inducer (**Figure** **1**). Programmed death is primarily classified into apoptosis and autophagy; however, recent reports have identified additional mechanisms, including ferroptosis, necroptosis, and pyroptosis.^[^ ^20^, ^21^ ^]^ Among these, pyroptosis is an inflammatory type of cell death related to caspase‐1 in response to external stimulation.^[^ ^22^ ^]^ The mechanism of caspase‐1 activation is well‐defined and has potential clinical applications. In this context, caspase‐1‐mediated pyroptosis using a photo‐activable inducer has gained attention as a targeted therapeutic strategy, leveraging the specificity of photo‐activation.

In this work, the NBSD series with different alkyl chain lengths were synthesized and evaluated. The alkylated chains induced the enhanced lipophilicity of NBSD, leading to specific aggregation patterns in aqueous solutions (Figure 1a). This patterning offers superior advantages over conventional PSs, including (i) aggregation‐enhanced PDT effect, (ii) high cellular uptake, (iii) induction of programmed cell death via pyroptosis, (iv) implantable properties, and (v) high biocompatibility. Notably, the aggregation pattern plays a crucial role in both ROS generation and the pyroptosis mechanism of NBSD (Figure 1b,c). Given that aggregation enhances ROS generation, the NBSD‐based PS was designed as a gel‐type formulation with strong adherence to the surgical cavity, allowing for pH‐controlled release (Figure 1d). Colorectal cancer recurs in 20–50% of patients at the resection site, even after curative surgery.^[^ ^23^, ^24^ ^]^ Post‐surgery, the tumor microenvironment gradually shifted to a weakly acidic condition (pH 5–7).^[^ ^25^ ^]^ The implantable NBSD gel released more NBSD at pH 6.0 compared to pH 7.4, indicating its outstanding potential to inhibit the initial tumor recurrence. Overall, this work highlights the importance of lipidation‐induced aggregate patterning and its biological application in preventing tumor recurrence through photoactivation‐induced programmed cell death.

## 2 Results and Discussion

### 2.1 Rationale

**Figure 2:**
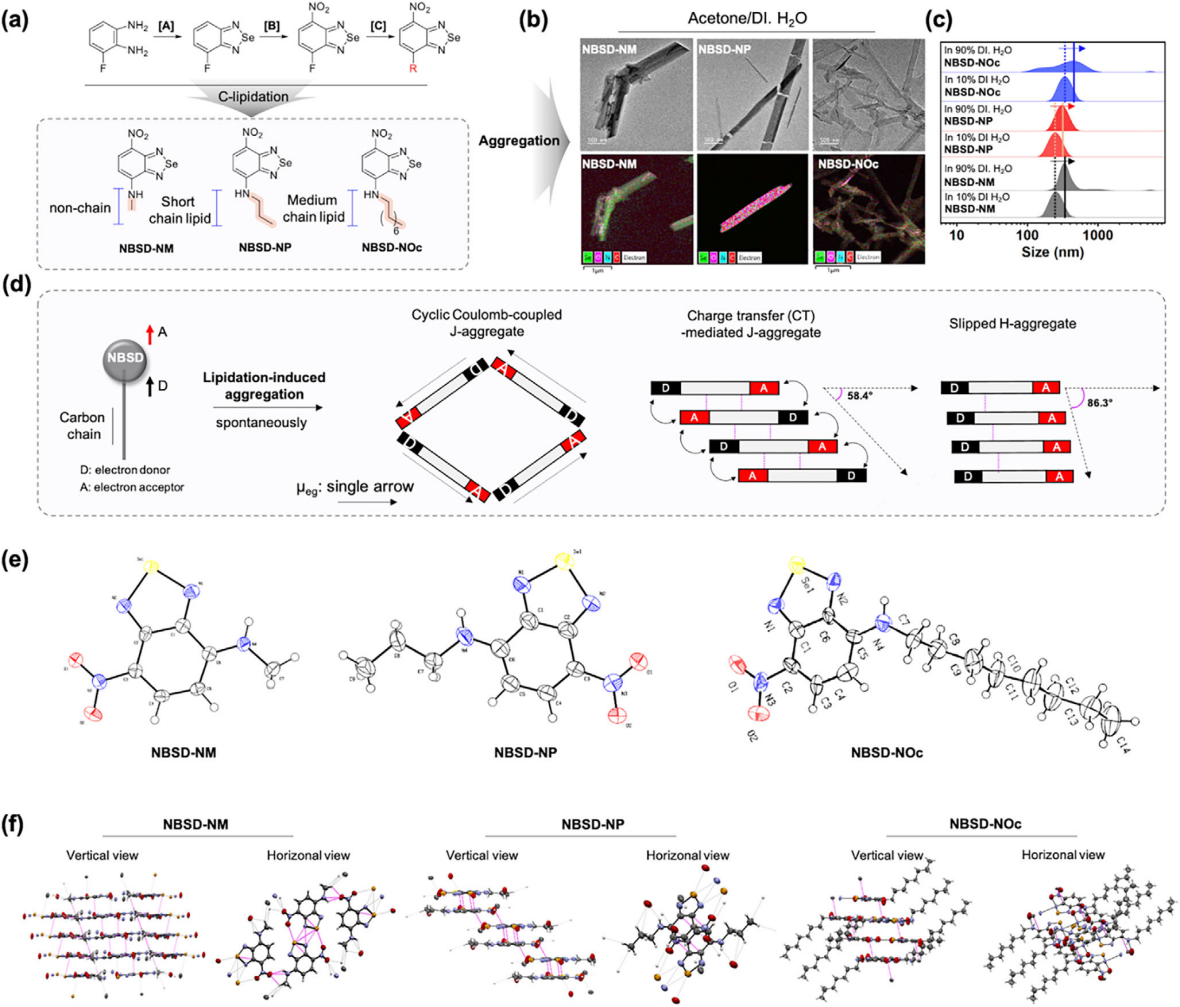
a) Synthetic scheme of the NBSD series (NBSD‐NM, NBSD‐NP, and NBSD‐NOc). Reagents and conditions: [A] SeO_2_, EtOH, reflux, 2 h. [B] HNO_3_, H_2_SO_4_, 0 °C, 10 min. [C] Corresponding alkyl amine (4 eq), ACN (0.3 M of 4‐fluoro‐nitrobenzoselenadizole), 25 °C, Yield: NBSD‐NM 68.9%, NBSD‐NP 81.2%, and NBSD‐NOc 82.6%. b) TEM/EDX analysis of the NBSD series after aggregate formulation in binary solution (10% acetone: 90% DI. H_2_O, *v/v*). c) DLS analysis in binary solutions (acetone/DI. H_2_O) with different percentages (*v/v*). See Table S1, Supporting Information. d) Classification of aggregation for lipidation‐induced NBSD structure. Single arrows indicate the µ_eg_, oscillation phase. NBSD‐NM: Cyclic Coulomb‐coupled J‐aggregate. NBSD‐NP: Charge transfer‐mediated J‐aggregate. NBSD‐NOc: Slipped H‐aggregate. e) Structure of the NBSD series verified by SC‐XRD. Yellow atom: selenium, Blue atom: nitrogen, Red atom: oxygen. Structural information is deposited in the Cambridge Crystallographic Data Centre (CCDC); deposit numbers: 2 421 472 (NBSD‐NM), 2 421 477 (NBSD‐NP), and 2 421 479 (NBSD‐NOc). See detailed information in Tables S2–S16, Supporting Information. f) SC‐XRD analysis using the crystal structure of the NBSD series. Purple line: pi‐pi interaction. Gray line: hydrogen bond or charge‐charge interaction.

To date, various PS core structures have been reported, including BODIPY, porphyrin, Ru(II) complexes, and metal‐free organic molecules.^[^ ^26^, ^27^, ^28^, ^29^ ^]^ Recently, our research group has focused on the development of a new molecular PS structure based on the NBSD core.^[^ ^30^ ^]^ In 2023, we reported an in situ activatable PS (named NCCNs), which forms a nano‐complex between NBSD and copper ions.^[^ ^30^ ^]^ NCCNs exhibited a turn‐on PDT effect upon reacting with glutathione in biological systems. In 2024, we introduced a controlled Smiles rearrangement approach using the NBSD structure, revealing distinct photophysical properties upon reaction with cysteine or homocysteine.^[^ ^31^ ^]^ Both studies demonstrated a turn‐on mechanism in their photophysical properties, including fluorescence and PDT. In this research scope, we have focused on understanding aggregation‐related photophysical and biological properties of the NBSD series by introducing alkyl chains of varying lengths. We also explored their practical applications to enhance PDT efficacy in tumor treatment. The alkylated chains contribute to structural lipophilicity and water solubility, leading us to hypothesize that variations in NBSD lipophilicity could result in distinct aggregate packing patterns. NBSD derivatives were constructed with different alkylated chain lengths: (i) NBSD‐NM (methylamine substitution), (ii) NBSD‐NP (propylamine substitution), and (iii) NBSD‐NOc (octylamine substitution). The synthesis of these NBSD derivatives is depicted in **Figure** **2**a. The NBSD series was synthesized via a straightforward three‐step process starting from 3‐fluorobenzene‐1,2‐diamine (see Supporting Information for details). Briefly, [Step 1] selenadiazole ring formation: is achieved by reducing SeO_2_ and reacting it with diamine, [Step 2] nitration reaction: is performed on the product from Step 1, and [Step 3] nucleophilic substitution reaction: introducing the alkylamine group into the aromatic substituent using triethylamine as a base. The synthesized NBSD series was characterized using NMR (^1^H, ^13^C) and MS (see Supporting Information).

### 2.2 Structural Characterization

For the structure characterization of the NBSD series, each compound was dissolved in either acetone or ethanol (EtOH). Deionized water (DI. H_2_O) was then added into the NBSD solution (100 µmol L^−1^) to achieve final concentrations of 10% (v/v) and 90% (v/v) DI.H2O. Transmission electron microscopy (TEM) imaging and energy‐dispersive X‐ray (EDX) spectroscopy were performed to analyze the aggregate shape of the NBSD series after negative staining with 2% (v/v) uranyl acetate in EtOH (Figure 2b, Figure S1, Supporting Information). The analysis revealed that all NBSD derivatives formed rod‐shaped aggregates, composed of Se, O, N, and C atoms. Next, we evaluated whether the alkyl group in the NBSD series affects the shape of aggregation. To verify this, we synthesized NBSD‐aniline as an aryl substitute instead of an alkyl group (Figure S2, Supporting Information). This compound demonstrated a rod‐shaped aggregate, indicating that the alkyl group does not significantly influence the aggregate shape. Recently, our group published findings on NBSD‐Chol, which consists of a carbon linker, cholesterol, and an NBSD core.^[^ ^32^ ^]^ This formulation exhibited a round‐shaped aggregate and a well‐defined polydispersity index (PDI). Therefore, it may be quite complex to control the aggregate shape. Next, we monitored the environment‐dependent average size of these rod‐shaped aggregates using dynamic light scattering (DLS) (Figure 2c, Figures S3 and S4, and Table S1, Supporting Information). The results demonstrated a shift in size distribution depending on the fraction of DI.H_2_O (from 10% to 90%). For NBSD‐NM in a binary solution containing acetone and DI. H_2_O, the size increased from 270.0 to 808.1 nm with a rise in the polydispersity index (PDI) value (0.25 to 0.82). For NBSD‐NP under the same conditions, the size was shifted to 375.6 nm, with a PDI value of 0.55. The size of NBSD‐NOc changed from 380.0 to 414.3 nm, with a corresponding PDI value change (0.15 to 0.46). In another binary solution containing DI. H_2_O and EtOH, the aggregated size of NBSD‐NM appeared unstable (Figure S4, Supporting Information). In contrast, NBSD‐NP exhibited size changes of 120.0, 198.1, and 505.4 nm in 10%, 50%, and 90% DI. H_2_O/EtOH solutions, respectively. For NBSD‐NOc, a size of 804.5 nm with a PDI of 0.636 was observed under 90% DI. H_2_O conditions. Based on TEM and DLS analyses, we confirmed the aggregation behavior of the NBSD series.

NBSD has a distinct push‐pull feature with electron‐donor (D) and electron‐acceptor (A) groups (Figure 2d).^[^ ^31^ ^]^ The NO2 group acts as an electron‐acceptor group, while the amine group pushes the electron, creating an intramolecular charge transfer (ICT) character in the NBSD backbone. ICT occurs under specific excitation, releasing energy through various pathways. NBSD has been reported to function as both a fluorophore and a PS when modified with a strong electron‐donating group, such as amine, at the electron‐donating site.^[^ ^33^ ^]^ These photophysical properties motivated us to evaluate the altered photophysical behavior of NBSD when packed into specific shapes, depending on the aggregate type influenced by the alkylamine group. Aggregate can broadly be divided into two types: J‐aggregates and H‐aggregates.^[^ ^34^ ^]^ The representative distinction between J‐aggregates and H‐aggregates lies in their altered configuration. J‐aggregates tend to adopt a head‐to‐tail configuration, while H‐aggregates show a face‐to‐face configuration. These different configurations caused altered charge‐transfer.

To confirm the aggregate packing pattern of the NBSD series, we conducted single crystal X‐ray diffraction (SC‐XRD) analysis (Figure 2e–h, Tables S2–S16, Supporting Information). The results confirmed that NBSD‐NM exhibited a cyclic Coulomb‐coupled J‐aggregate, which displayed a rotated configuration with a repeated D‐A (donor‐acceptor) type arrangement.^[^ ^35^ ^]^ As shown in Figure 2f, the NH group within the NBSD backbone formed an H‐bonding with the oxygen atom of the NO2 group from another NBSD molecule, while the CH_2_ group bonded with the same oxygen position. Another oxygen of NO_2_ is connected to the selenium atom (Se) of a neighboring NBSD molecule via a chalcogen bond (Se–O). The length of the π‐π conjugation is 3.401 Å (C2 to C3). NBSD‐NP demonstrated a charge‐transfer‐mediated J‐aggregate with a slip angle of ϴ = 58.4° (Figure 2g).^[^ ^35^ ^]^ The configuration was based on the π‐π conjugation (C3 to C6: 3.400 Å), and the selenium of the selenadiazole ring was bound to the oxygen of the NO_2_ group and the amine of the selenadiazole ring through a chalcogen bond. The packing pattern was dimer‐based. Last, NBSD‐NOc exhibited a slipped H‐aggregated shape with a slip angle of ϴ = 86.3° (Figure 2h).^[^ ^36^ ^]^ The packing was driven by π‐π conjugation (3.413 Å) between C1 and the C5. The association of the alkyl chain for π‐π stacking began with NBSD‐NP, indicating that a longer alkyl chain induced unstable π‐π conjugation in each group upon aggregation. To confirm the aggregation, we performed MM2/MD simulation (Step interval: 2 fs, frame interval: 10 fs, termination step: 40 000, heating/cooling rate: 1.0 Kcal/atom/ps, target temperature: 300 Kelvin) using the dimer form (Figure S5a, Supporting Information). We confirmed significant charge‐charge interactions between the NO_2_ group, where attractive interactions were observed between N+ and O‐ in each molecule. The distance between molecules decreased from NBSD‐NM to NBSD‐NOc (NBSD‐NM: 2.7 Å, NBSD‐NP: 2.55 Å, and NBSD‐NOc: 2.39 Å). This suggests the charge‐charge interaction affects the stability of the π‐π interaction and the packing pattern of the aggregate. Additionally, the alkylation length of NBSD was found to be related to lipophilicity (Figure S5b, Supporting Information). Overall, alkylation of NBSD significantly influenced the aggregation pattern by modulating key interactions such as π‐π stacking, charge‐charge interactions, and lipophilicity, highlighting its potential to control molecular packing and photophysical properties.

### 2.3 Photophysical Properties of the NBSD Series

**Figure 3:**
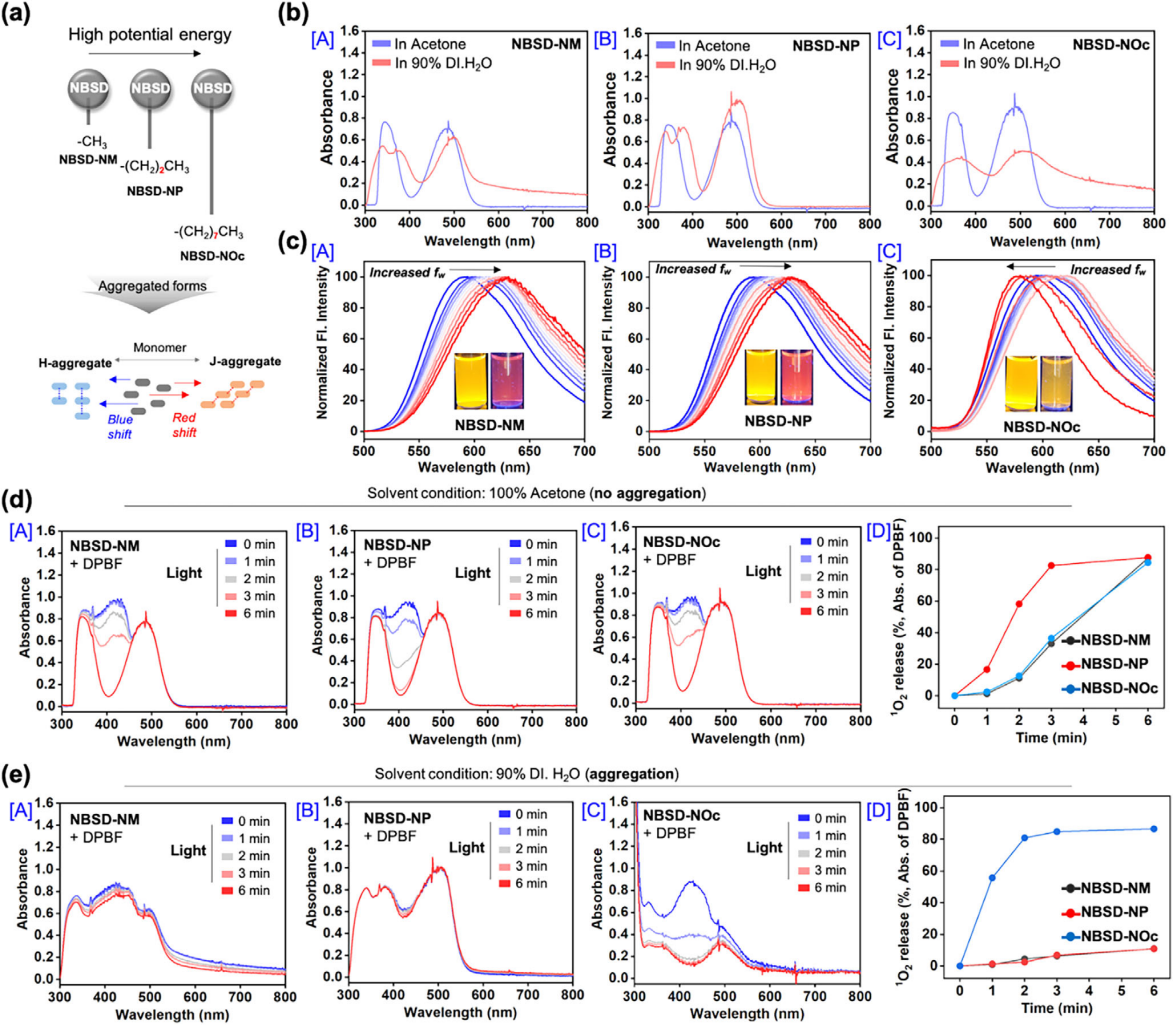
a) Schematic illustration indicating the different photophysical properties based on the aggregation pattern caused by tailoring the alkyl chain length. b) Absorption spectra of the NBSD series. c) Emission spectra of the NBSD series. Insert: photos of the solutions under UV light (365 nm). Left: in acetone; Right: in 10% acetone in DI. H_2_O. b,c) [A]: NBSD‐NM, [B]: NBSD‐NP, and [C]: NBSD‐NOc, each measured in EtOH/DI. H_2_O (0% EtOH to 90% EtOH). The concentration of the NBSD series was fixed at 100 µM. The emission spectra were recorded under excitation at 490 nm. d) Absorption spectra of DPBF (concentration: 80 µM) in the presence of each NBSD derivative (concentration: 100 µM). e) Emission spectra of DPBF (concentration: 80 µM) in the presence of each NBSD derivative (concentration: 100 µM). d,e) [A]: NBSD‐NM, [B]: NBSD‐NP, [C]: NBSD‐NOc, and [D] the release plot analysis for ^1^O_2_. Solvent: Acetone. Light: 530 nm, 75 mW/cm^2^. The release of ^1^O_2_ was quantitatively measured using the formula: (Absorption of DPBF at 80 µM – absorption of acetone) × 100 = 100% ^1^O_2_ release.

It is well‐known that alkylated chains induce structural potential energy due to several factors, including enhanced Van der Waals interactions, steric hindrance, and increased flexibility.^[^ ^37^, ^38^, ^39^ ^]^ To reduce this potential energy, the molecules were recruited and then aggregated (**Figure** **3**a). In photophysical science, H‐aggregated molecules adopt a face‐to‐face stacking conformation with strong intermolecular coupling, resulting in exciton energy level splitting and allowing transitions to occur at higher energy.^[^ ^34^ ^]^ In this case, the emission is quenched due to the forbidden transition. In contrast, J‐aggregates adopt a head‐to‐tail configuration, leading to a different exciton coupling that permits electronic transitions at lower energy, shifting both absorption and emission to longer wavelengths.^[^ ^34^ ^]^ Following this, we conducted a photophysical assay to confirm the spectral shift and intensity changes upon aggregation of the NBSD series. First, we performed an absorption analysis in a binary solution containing DI. H_2_O/acetone and DI. H_2_O/EtOH (Figure 3b, Figure S6, Supporting Information). The absorbance peak of NBSD‐NM at 490 nm was red‐shifted to 513 nm in 90% DI. H_2_O/10% acetone, accompanied by reduced absorptivity. Under the same conditions, the absorption at 350 nm split into two peaks at 340 nm and 372 nm, which could be attributed to strong charge‐transfer interactions. As presented in Figure S6, Supporting Information, in the DI.H_2_O/EtOH binary solution, the absorption spectrum of NBSD‐NM exhibited a decrease in intensity and a red shift from ≈490 to 498 nm. Meanwhile, NBSD‐NP in DI. H_2_O/EtOH demonstrated a redshift without a decrease in absorptivity. In contrast, the absorption spectrum of NBSD‐NOc in DI. H_2_O/EtOH displayed a reduction in fluorescence intensity, though the shift was not clearly observed. According to previous studies, excessively close charge‐transfer interactions can interfere with electronic transitions, leading to lower molar absorptivity. NBSD‐NP exhibited a similar redshift (from 491 to 515 nm) as NBSD‐NM, but with a slightly increased molar absorption coefficient, likely due to charge‐transfer interactions. Based on the SC‐XRD data of NBSD‐NP (Figure 2g), the distance between the electron‐donating and electron‐withdrawing groups in the packed structure was measured to be 3.4–3.7 Å, whereas the intramolecular distance between these groups within a single molecule was 5.7 Å. This suggests that charge‐transfer efficiency is enhanced upon the aggregation of NBSD‐NP due to their specific pattern. In contrast, the absorption spectrum of NBSD‐NOc remained unchanged, except for a decrease in intensity at 500 nm. Additionally, the absorption splitting observed at 350 nm was absent, indicating that NBSD‐NOc was not significantly influenced by charge‐transfer interactions upon structural aggregation. According to the SC‐XRD structure (Figure 2g), NBSD‐NOc exhibited a slipped H‐aggregate with a π‐π conjugation at a distance of 3.413 Å. The long alkyl chain of NBSD‐NOc may have induced instability in the π‐π conjugation distance under light exposure, leading to weakened excitonic interactions and minimal energy level fluctuations. Consequently, this instability likely prevented a pronounced blue shift, while also causing quenching of absorption at 500 nm. In terms of fluorescence, J‐aggregates exhibit a redshift, whereas H‐aggregates tend to show a blue shift. To further investigate these spectral changes, we performed fluorescence analysis to monitor wavelength shifts upon aggregation of the NBSD series in binary solutions, such as acetone/DI. H_2_O and EtOH/DI. H_2_O (Figure 3c, Figures S6b,S7 and S8, Supporting Information). As shown in Figure 3c [A] and Figure S8, Supporting Information, the fluorescence of NBSD‐NM displayed a red shift from 603 to 630 nm in EtOH/DI. H_2_O (range: 594 to 632 nm in acetone/DI. H_2_O, Figure S7a, Supporting Information). NBSD‐NP followed a similar trend, with its emission shifting from 606 nm to 633 nm in EtOH/DI. H_2_O (Figure 3c [B]) and from 594 to 629 nm in acetone/DI. H_2_O (Figure S7b, Supporting Information). In contrast, NBSD‐NOc demonstrated a distinct shift in both binary solutions. At 0 to 50% water, its fluorescence initially red‐shifted, but upon reaching 90% water, it blue‐shifted (EtOH/DI. H_2_O: 609 to 582 nm, Figure 3c [C]; acetone/DI. H_2_O: 592 to 580 nm, Figure S7c, Supporting Information). We hypothesized that as radiative emission decreases in aggregates, the ISC to the triplet state would increase, thereby enhancing the efficiency of ROS generation. To confirm the generation of ^1^O_2_, a DPBF assay was conducted under both non‐aggregate and aggregate conditions using binary solutions of EtOH/DI. H_2_O and acetone/DI. H_2_O.^[^ ^30^ ^]^ In the non‐aggregate state in acetone, the NBSD series demonstrated high ^1^O_2_ generation under light exposure (530 nm within 6 min) (Figure 3d). Among them, NBSD‐NP exhibited the fastest ^1^O_2_ generation, while NBSD‐NP and NBSD‐NOc displayed a similar trend. ^1^O_2_ generation was faster in EtOH than in acetone across all cases (Figure S9a, Supporting Information). As shown in Figure 1, 3_2_ generation was not observed in aggregated NBSD‐NM and NBSD‐NP (Figure 3e [A],[B],[D]). However, in the case of NBSD‐NOc, the ^1^O_2_ generation rate was faster in the aggregated state than in the non‐aggregated state (Figure 3e[C],[D]). In EtOH/DI. H_2_O aggregated NBSD‐NP exhibited ^1^O_2_ generation, although with reduced efficiency compared to its non‐aggregated form (Figure S9b, Supporting Information). NBSD‐NOc in EtOH/DI. H_2_O showed a similar ^1^O_2_ generation performance to that observed in acetone/DI. H_2_O. For the NBSD‐aniline as aryl substitute on NBSD, we confirmed that it exhibited a quenching of emission intensity under 90% DI water conditions when compared to NBSD‐NOc (Figure S10a, Supporting Information). As shown in Figure S10b, Supporting Information, this difference is quite evident. In terms of ROS generation, NBSD‐aniline demonstrated superior performance in 90% DI water compared to NBSD‐NM and NBSD‐NP (Figure S10c,d, Supporting Information). Therefore, we concluded that NBSD‐aniline is unsuitable as a candidate for our study because of quenching properties for emission. Generally, many photosensitizers experience quenched ^1^O_2_ generation in their aggregated form due to several reasons such as decreased charge transfer, π‐π stacking‐induced thermal relaxation, and hindered O_2_ diffusion caused by π‐π stacking. Taken together, the characteristic slipped H‐aggregate formed by the long alkyl chain resulted in the longest π‐π stacking distance (3.413 Å). Unlike other NBSD derivatives, whose aggregation was largely driven by charge‐transfer interactions, NBSD‐NOc maintained its ^1^O_2_ generation efficiency even in the aggregated state.

### 2.4 Photo‐Activable NBSD as a Pyroptosis Inducer

**Figure 4:**
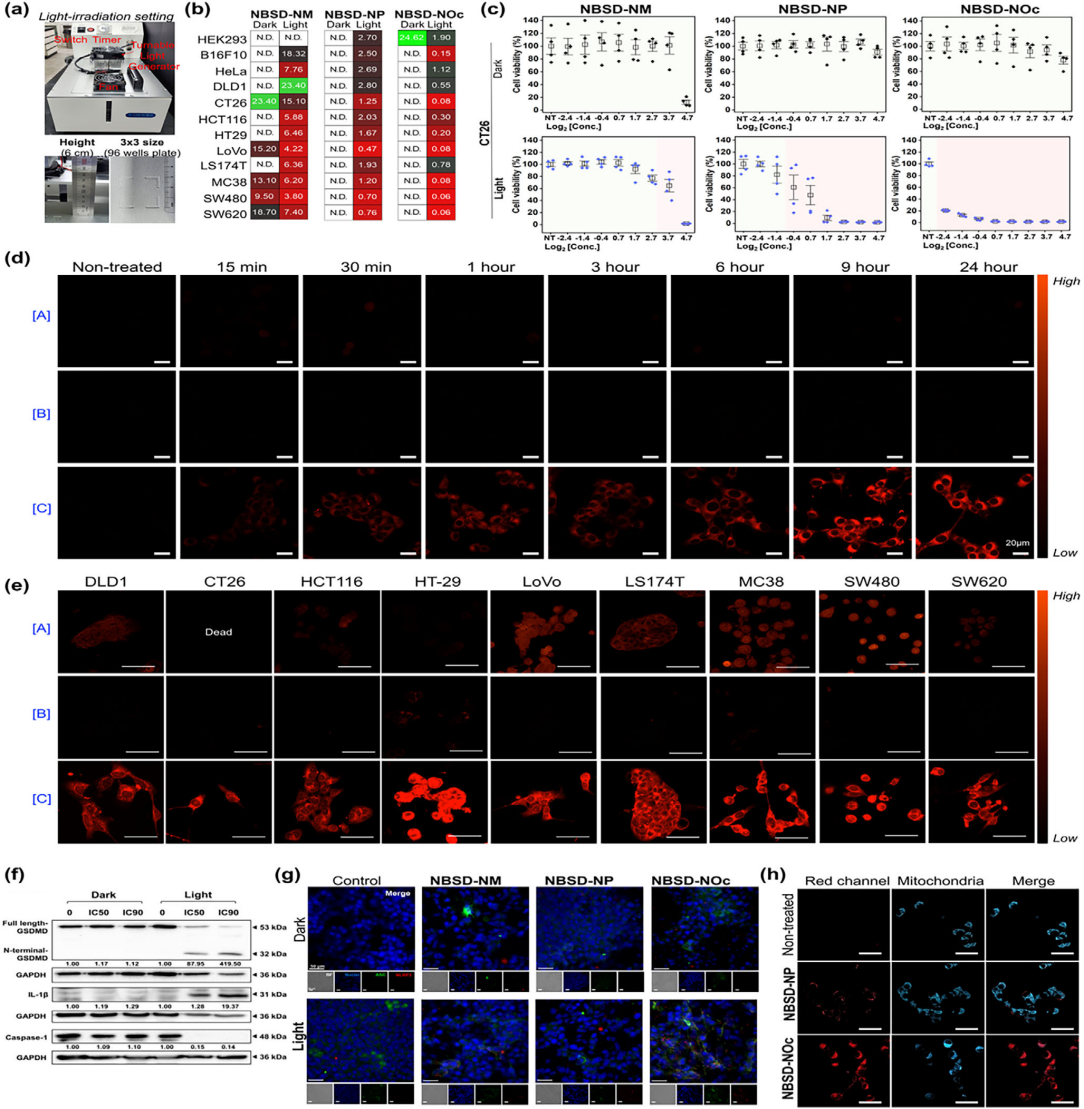
a) Instrument setting for light irradiation (530 nm, 75 mW cm^−2^). An irradiated space of 3 cm × 3 cm size was used for 96‐well plate cell tests. b) Cellular toxicity mapping under dark/light conditions. Unit: µM. Incubation time: 48 h (24 h after light irradiation, 530 nm, 75 mW cm^−2^, 3 min). Cell viability was evaluated by absorption intensity using the CCK‐8 assay. c) CT26 cell (colorectal cancer, BALB/c mice) viability graph under dark/light conditions. The red zone indicates significant areas of cell viability. Error bars represent mean ± S.D. (n = 4). Results were repeated from independent conditions (location, time, and researcher). d) Cellular uptake analysis over time (15 min to 24 h). [A]: NBSD‐NM, [B]: NBSD‐NP, and [C]: NBSD‐NOc. CT26 cells were used. Scale bar: 20 µM. Compound concentrations: 20 µM. e) Cellular uptake analysis in various cell lines (DLD1, CT26, HCT116, HT29, LoVo, LS174T, MC38, and SW620). [A]: NBSD‐NM, [B]: NBSD‐NP, and [C]: NBSD‐NOc. Compound concentrations: 20 µM. Incubation: 24 h. () Western blot analysis for the pyroptosis mechanism. Protein loading: 30 µg. CT26 cells were treated with NBSD‐NM, NBSD‐NP, or NBSD‐NOc at their respective IC_50_ and IC_90_ concentrations. Data for NBSD‐NM and NBSD‐NP are presented in Figure S20, Supporting Information. g) Immunofluorescence (IF) analysis of CT26 cells after treatment of the NBSD series under dark/light conditions (530 nm, 75 mW cm^−2^, 3 min). h) Mitochondrial co‐localization analysis of NBSD‐NP and NBSD‐NOc by confocal microscopy. Cells were treated with 50 µM of NBSD‐NP and NBSD‐NOc for 30 min, followed by a 15‐min treatment with a mitochondria tracker. Scale bar: 50 µm. Ex/Emi: 405 nm/415–504 nm (mitochondria), 488 nm/510–700 nm (NBSD‐NP and NBSD‐NOc). Laser power: 3% (mitochondria) and 1% (NBSD‐NP and NBSD‐NOc). Incubation: 4 h. The images are obtained without washing step.

Given that NBSD‐NOc exhibits promising photosensitizing properties even in its aggregate state, we evaluated the in vitro photo‐induced toxicity of NBSD‐NOc across twelve different cell lines. To minimize experimental error, we standardized the light irradiation setup (530 nm, 75 mW cm^−2^) (**Figure** **4**a). Using this setup, we performed photo‐induced toxicity assays for NBSD‐NM, NBSD‐NP, and NBSD‐NOc. To assess the in vitro effects, we measured cell viability in non‐colon cancer cell lines (HEK293, B16F10, and HeLa) and colon cancer cell lines, including seven human cell lines (HT29, HCT116, LoVo, SW480, SW620, LS174T, and DLD1) and two mouse cell lines (MC38 and CT26), with or without light exposure (530 nm, 75 mW cm^−2^, 3 min) (Figure 4b and Figures S11–S13, Supporting Information). NBSD‐NOc demonstrated the most potent photo‐induced toxicity effect, with sub‐micromolar IC50 values in all cell lines. In particular, the IC50 for light‐exposed NBSD‐NOc was 0.73 µM in LS174T, 0.56 µM in DLD1, 0.29 µM in HCT116, and 0.21 µM in HT29 cells. In other cell lines, the IC50 was below 0.19 µM. In contrast, NBSD‐NM showed IC50 values between 5 to 15 µM under light irradiation, while NBSD‐NP demonstrated IC50 values ≈2 µM, depending on the cell line. Specific IC50 values of each condition are presented in Figure 4b. Overall, the NBSD series exhibited outstanding performance in inducing photo‐induced cell death, with the CT26 cell line showing the most significant in vitro effect. In the CT26 cell line, the photo‐induced toxicity of NBSD‐NOc yielded consistent results with narrow error bars (mean ± S.D.), and the red zone representing the toxic region expanded from NBSD‐NM to NBSD‐NOc (Figure 4c). This result was further supported by the DPBF assay using NBSD‐NOc. Following this, we evaluated the cellular uptake of the NBSD series (A: NBSD‐NM, B: NBSD‐NP, C: NBSD‐NOc) in the CT26 cell line (Figure 4d and Figure S14, Supporting Information). Both NBSD‐NM and NBSD‐NP exhibited relatively low fluorescence efficiency compared to NBSD‐NOc at a concentration of 20 µM. NBSD‐NOc displayed strong emission intensities within the cell membrane, with intensity levels depending on the uptake time (15 min to 24 h). At this stage, we questioned whether all the tested cell lines would follow a similar pattern, with NBSD‐NM and NBSD‐NP exhibiting poor or low uptake efficiency, while NBSD‐NOc demonstrated strong uptake. To address this, we performed the uptake analysis using nine colon cancer cell lines (DLD1, CT26, HCT116, HT29, LoVo, LS174T, MC38, SW480, and SW620) (Figure 4e and Figure S15, Supporting Information). After incubating the NBSD series (A: NBSD‐NM, B: NBSD‐NP, C: NBSD‐NOc) for 24 h, the nine colon cancer cell lines were analyzed using a confocal microscope. To visualize uptake at the maximum point, a concentration of 50 µM for the NBSD series was used. Under these conditions, NBSD‐NM was observed in seven colon cancer cell lines (DLD1, HCT116, LoVo, LS174T, MC38, SW480, and SW620), but it did not appear to penetrate HT29 cells. NBSD‐NP showed no uptake in any of the tested cell lines. However, NBSD‐NOc was observed in all tested cell lines, with confocal images displaying a dot‐staining pattern in the cytosol. Cellular uptake of NBSD series was further confirmed with reading absorbance in the culture medium of NBSD‐treated CT26 cells, and the results indicated the higher cellular uptakes of NBSD‐NM and NBSD‐NOc compared with NBSD‐NP (Figure S16, Supporting Information). Assays to determine live and dead CT26 cells confirmed the cytotoxicity of NBSD series, particularly high in NBSD‐NOc group (Figure S17a, Supporting Information), with a different cell death mechanism pathway rather than apoptosis suggested (Figure S17b, Supporting Information). Therefore, we conducted experiments to further investigate the mechanisms of cell death (Figure 4h, Figures S18, and S19, Supporting Information). Pyroptosis, a recently discovered form of programmed cell death related to inflammatory responses, is strongly linked to ROS.^[^ ^40^ ^]^ ROS‐based PDTs have proven effective as a powerful strategy for pyroptosis induction in cancer cells.^[^ ^41^, ^42^ ^]^ First, to validate the observed cytotoxicity by NBSD series was specifically mediated by pyroptosis, and to exclude the possible involvements of other programmed cell death mechanisms, we performed inhibitor studies. The NBSD series‐induced CT26 cell death was significantly reduced upon inhibition of NLRP3 with MCC950, but not by caspase 3/7 inhibition (Z‐DEVD‐FMK) or MLKL inhibition (NEC‐1s), indicating the effect is pyroptosis‐dependent (Figure S18a, Supporting Information). Pyroptosis is characterized by continuous swelling, rupture of the cell membrane, and the release of cell contents,^[^ ^43^ ^]^ with gasdermin proteins, particularly GSDMD.^[^ ^44^ ^]^ Membrane rupture was notable in NBSD‐NOc, while NBSD‐NM and NBSD‐NP showed less effect (Figure S19, Supporting Information). The higher cellular uptake level and membrane rupture property of NBSD‐NOc than the other derivatives is caused by the carbon length, which is similar to the cholesterol size, which is a membrane‐binding and permeable molecule. All three NBSD variants induced GSDMD cleavage after CT26 cells were exposed to light, with a notable increase in GSDMD cleavage observed in NBSD‐NOc. The expressions of other factors related to pyroptotic cell death showed similar trends. Caspase‐1, an upstream caspase in the canonical pyroptosis pathway, is cleaved upon activation,^[^ ^45^ ^]^ which in turn cleaves pro‐IL‐1β to produce the mature form of IL‐1β, a cytokine.^[^ ^46^ ^]^ While NBSD‐NM and NBSD‐NP induced decreased pro‐caspase‐1 levels and increased IL‐1β under light exposure, NBSD‐NOc showed more significant changes in these two factors (Figure 4g and Figure S20, Supporting Information). Cytokine measurements also demonstrated that the NBSD series induced IL‐1β release, especially after light exposure (Figure S21, Supporting Information). We noted that NBSD‐NM altered IL‐1β expression in the culture medium even before light exposure. A similar trend was observed in IL‐18 levels, another cytokine frequently associated with pyroptosis.^[^ ^47^ ^]^ The NLRP3‐dependent pathway is currently the most studied ROS‐induced pyroptosis pathway. NLRP3 acts as a sensor that responds to various PAMPs and DAMPs,^[^ ^48^ ^]^ including intracellular ROS.^[^ ^49^ ^]^ Upon activation, NLRP3 assembled an inflammasome with ASC and pro‐caspase‐1.^[^ ^50^ ^]^ To investigate this, we stained NBSD‐treated CT26 cells for NLRP3 and ASC (Figure 4h). NBSD‐NOc significantly increased the expressions and co‐localization of NLRP3 and ASC, indicating inflammasome assembly. While NBSD‐NM and NBSD‐NP also induced an increase in NLRP3 and ASC expression, these were less pronounced compared to NBSD‐NOc, though still higher than in control CT26 cells. NBSD‐NM showed 2.0 ± 2.0 puncta/field, while NBSD‐NP showed 18.3 ± 5.1 and NBSD‐NOc showed 63.0 ± 6.1 puncta/field after light exposure. When NLRP3 was inhibited by MCC950 pre‐treatment, NBSD series were not able to alter these pyroptosis‐related markers. However, apoptosis inhibition or necroptosis inhibition did not affect this (Figure S18b, Supporting Information). Pyroptosis is closely linked to immune responses. In particular, pyroptotic cancer cell death can stimulate the immune system to immunologically transform “cold” tumors into “hot” tumors.^[^ ^51^ ^]^ Therefore, we investigated how NBSD series‐induced CT26 death affects macrophages by treating conditioned medium (CM) to Raw264.7 murine macrophages. CM from NBSD‐NOc notably increased the protein levels of CD86 and TNFα, suggesting M1 polarization of macrophages.^[^ ^51^ ^]^ NO production and IFNγ level in these macrophages further support the immune activation by NBSD series (Figure S22, Supporting Information). Mitochondrial ROS damage plays a crucial role in the formation of the NLRP inflammasome and the activation of caspase‐1. Upon activation, caspase‐1 cleaves GSDMD, initiating pyroptosis‐mediated cell death. To study this further, a colocalization assay using MitoTracker was performed (Figure 4i). The MitoTracker staining revealed that NBSD‐NOc accumulates near the mitochondria, correlating with its highest pyroptotic effect.^[^ ^52^, ^53^ ^]^ In contrast, NBSD‐NP was observed at the cell membrane, which may be due to the presence of the propyl group, a short chain. Previous studies have confirmed that lipidated molecules with chains ranging from propyl to hexyl tend to localize on the cellular membrane.^[^ ^54^ ^]^ Type I reaction by PDT produces diverse ROS such as superoxide anion (O_2_^‐^) and hydroxyl radical (OH) via electron transfer, and type II reaction generates ^1^O_2_ by energy transfer.^[^ ^55^ ^]^ Therefore, we measured ROS generation after treating CT26 cells with NBSD PDT (Figure S23a–c, Supporting Information). An increase in both total ROS and O_2_^‐^ was seen by NBSD‐NP, whereas NBSD‐NM showed no change. NBSD‐NOc resulted in significantly higher total ROS and O_2_^‐^. Notably, OH levels were only increased by NBSD‐NOc, suggesting its beneficial action regarding hypoxia in the tumor microenvironment.^[^ ^56^ ^]^ Additionally, we also observed a significant increase in ^1^O_2_ by NBSD‐NOc in the CT26 cells (Figure S23d, Supporting Information). Taken together, these results suggest that the NBSD series triggers pyroptotic cell death in colon cancer cells, with NBSD‐NOc exhibiting the highest potency.

### 2.5 Implantable NBSD‐NOc

**Figure 5:**
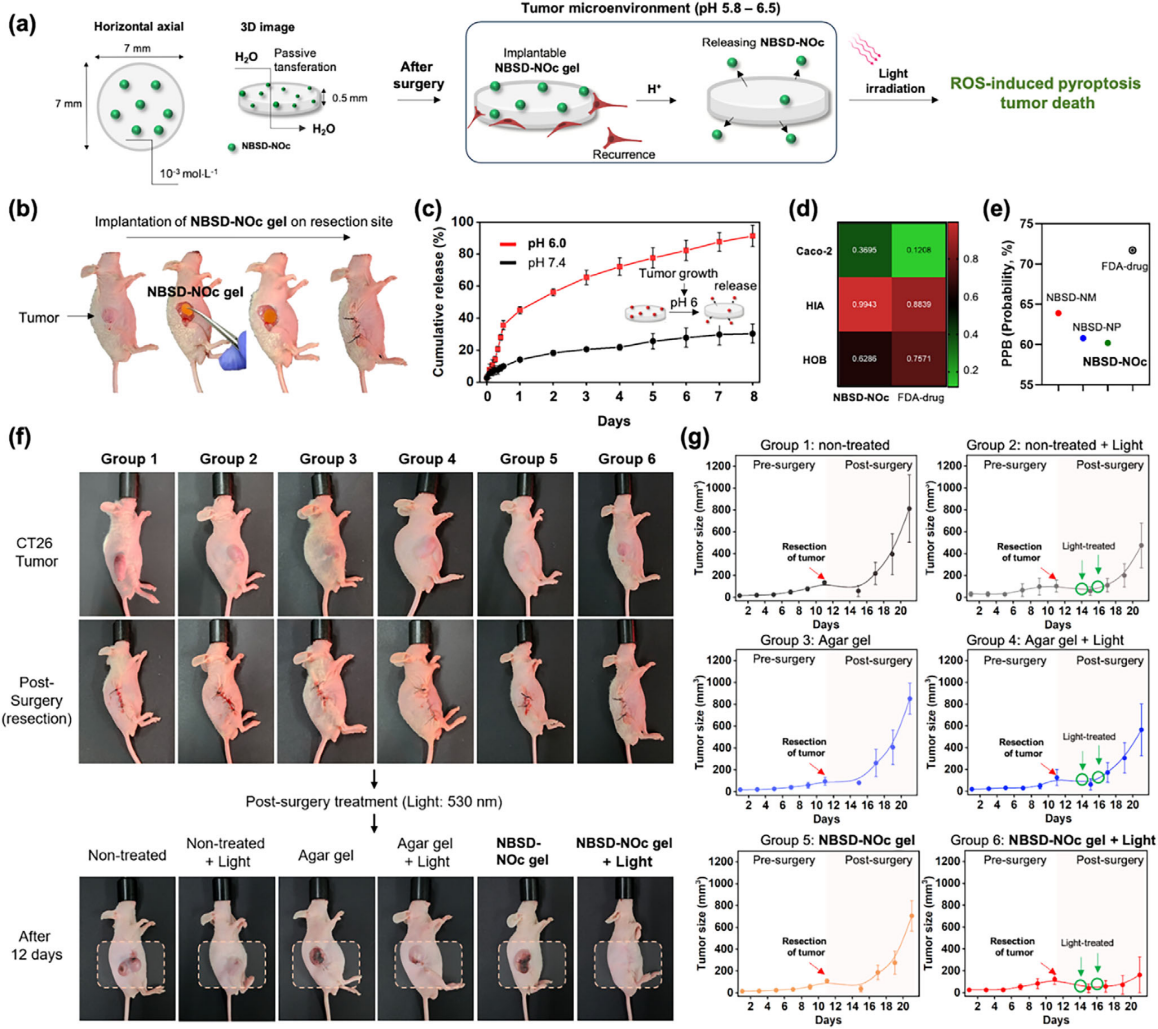
a) Schematic illustration of the NBSD‐NOc gel strategy, showing pH‐induced release in the tumor microenvironment (pH 5.8–6.5) upon light irradiation, followed by generation of reactive oxygen species (ROS)‐induced pyroptosis. b) Images demonstrating the use of implantable NBSD‐NOc gel after tumor resection. c) pH‐induced release profile of NBSD‐NOc gel. The error bars represent mean ± S.D. (n = 3). Computational pharmacokinetic profile for d) drug administration and e) plasma protein binding (%) using Swiss‐ADME prediction (accuracy: 72 – 94%, (58)). Fluorouracil, an FDA‐approved drug for human colorectal cancer, was used as a reference. f) Images showing pre‐surgery, post‐surgery with NBSD‐NOc gel implantation, and 12 days after surgery. Light irradiation (530 nm, 3 min, 75 mW cm^−2^) was performed using the same instrument as in Figure 4a. Group 1: non‐treated. Group 2: non‐treated + light. Group 3: 1% agar gel. Group 4: 1% agar gel + light. Group 5: NBSD‐NOc gel. Group 6: NBSD‐NOc gel + light. g) Tumor size monitoring (mm^3^). Group 1: non‐treated. Group 2: non‐treated + light. Group 3: 1% agar gel. Group 4: 1% agar gel + light. Group 5: NBSD‐NOc gel. Group 6: NBSD‐NOc gel + light. A tumor resection was performed on the 11th day. Green circles indicate the days of light treatment.

The standard treatment for non‐metastatic colorectal cancer (TNM Stages I‐III, UICC) is surgery.^[^ ^23^ ^]^ However, tumor recurrence, particularly in colorectal cancer, remains a major challenge that requires close monitoring after surgical resection. Various adjuvant therapies have been developed,^[^ ^57^ ^]^ but their overall efficacy is often limited due to issues related to molecular aggregation and targeted delivery.^[^ ^58^ ^]^ Direct access to the tumor microenvironment (TME) presents a valuable opportunity for effective therapy.^[^ ^59^ ^]^ In this context, a novel post‐surgical strategy utilizing hydrogels for controlled drug release has garnered growing interest. Given the global clinical interest in this approach, we hypothesized that NBSD‐NOc and its hydrogel formulation could serve as a promising therapeutic option due to its enhanced PDT effect and its role as a pyroptosis inducer. In aspect of limitation point, NBSD series can be excited below 600 nm, which has light penetration issue in deep tissue. Colorectal cancer, however, is a favorable model in this regard, as surgical access is often achieved using an endoscope equipped with a light source. In addition, colorectal cancer primarily develops on the epithelial layer of colon. The normal colon wall thickness was considered by less than and equal to 3 mm, while the pathological feature such as inflammation and cancer can increase the thickness more than 5 mm.^[^ ^60^, ^61^ ^]^ In this flow, the penetration depth of light in the 500–600 nm range has been reported to reach ≈5 mm, which we consider sufficient for initial optical access using endoscopic surgery and endoscopic post‐management. The hydrogel containing NBSD‐NOc was prepared using agarose (**Figure** **5**a). This hydrogel, with an NBSD‐NOc concentration of 10^−3^ mol·L^−1^, has a round shape with a diameter of 7 mm and a thickness of 0.5 mm. Additionally, the agarose‐based hydrogel allows for water permeability. The TME is closely associated with tumor progression, including proliferation, invasion, and metastasis.^[^ ^62^ ^]^ The extracellular matrix (ECM) within the TME provides spatial and structural support for tumor cells, making it an essential component of tumor growth. To mimic the ECM, various hydrogels, such as agarose, collagen, and Matrigel, have been developed and utilized.^[^ ^63^ ^]^ Based on this concept, we hypothesized that tumor cells initiating recurrence might preferentially attach to the NBSD‐NOc hydrogel for growth, recognizing it as an ECM‐like structure. In addition, as the recurrent tumor proliferates on the NBSD‐NOc gel, the pH may change to a weakly acidic condition (pH 5.8–6.5), a representative feature of the TME. Using the TME characterization, we hypothesized that the solubility and diffusion rate of NBSD‐NOc would increase at pH 6 due to changes in its charge state and alterations in hydrogen bonding within the agarose gel, whereas its release rate would slow down at pH 7.4. After release, NBSD‐NOc was expected to induce ROS‐mediated pyroptosis in the attached tumor cells under light irradiation. Next, we demonstrated that the round‐shaped NBSD‐NOc gel can be applied at the resected site following tumor removal (Figure 5b). To verify the release rate of NBSD‐NOc under weakly acidic conditions, we conducted a systematic analysis to monitor its release (Figure 5c). Fluorescent‐based monitoring was performed under different conditions, using pH 6 and pH 7.4 buffers for 9 days. The results showed a gradual increase in NBSD‐NOc release in the pH 6 buffer, whereas a more sustained release pattern was observed in the pH 7.4 buffer. These findings suggest that NBSD‐NOc is preferentially released when the TME shifts to a weakly acid state, indicating tumor recurrence.

We further performed an ADMET analysis (Swiss ADMET) to confirm the pharmacokinetic properties of NBSD‐NOc, including absorption, distribution, metabolism, excretion, and toxicity (Figure 5d).^[^ ^64^ ^]^ As a positive control, we assessed Fluorouracil, an FDA‐approved drug for colorectal cancer, alongside the NBSD series. The analysis confirmed that NBSD‐NOc demonstrated superior properties compared to Fluorouracil in terms of high Caco‐2 cell permeability and human intestinal absorption. Additionally, the presence of a long alkyl chain reduced permeability to Caco‐2 cells while slightly increasing HIA (Figure S24a, Supporting Information). Regarding unexpected protein binding, NBSD‐NM and NBSD‐NP showed a higher likelihood of binding to hormone‐related proteins compared to Fluorouracil (Figure S24b, Supporting Information). In contrast, NBSD‐NOc demonstrated a lower binding affinity for hormone‐related proteins than Fluorouracil. For Ames mutagenesis, carcinogenicity, and micronuclear toxicity assessments (Figure S24c, Supporting Information), NBSD‐NOc displayed either lower or similar toxicity levels relative to Fluorouracil. In terms of plasma protein binding (PPB), the NBSD series exhibited an improved performance over Fluorouracil (Figure 5e). Taken together, the combination of high HIA and low PPB suggests that NBSD‐NOc could be utilized as an oral or intravenous (i.v.) administration.

### 2.6 In Vivo Studies in CT26 Xenograft Model

To evaluate the inhibition of tumor recurrence using NBSD‐NOc gel, we developed a CT26 xenograft model with Matrigel (1 × 10^7^ cells/50 µL + 50 µL Matrigel; Corning Matrigel Matrix, Cat. No.: 356 234) (Figure 5f). The CT26 tumor grew to 108.74 mm^3^ by day 11. Afterward, we resected the tumor site and implanted the NBSD‐NOc gel at the site of resection. For post‐surgery treatment, the NBSD‐NOc gel was exposed to light (530 nm, 75 mW cm^−2^, 3 min). To evaluate its therapeutic ability, we established six groups (n = 5): non‐treated (group 1), non‐treated + light (group 2), agar gel (group 3), agar gel + light (group 4), NBSD‐NOc gel (group 5), and NBSD‐NOc gel + light (group 6). Light irradiation was performed twice (on days 3 and 5) post‐surgery. As shown in Figure 5f,g, the number of recurrent tumors was significantly lower in the NBSD‐NOc gel‐treated group compared to other conditions, indicating that the NBSD‐NOc gel effectively inhibited recurrence without causing light toxicity. Histological assessments further supported the tumor‐reducing effect of NBSD‐NOc gel. NBSD‐NOc with light treatment resulted in marked tumor cell disintegration and widespread necrosis, whereas minimal histological changes were observed in the control and gel‐only groups (Figure S25, Supporting Information). Tables S17–S22, Supporting Information provides a record of tumor growth over 21 days. To assess in vivo toxicity, we conducted experiments to determine whether NBSD‐NOc exhibited toxicity when released into the bloodstream. Additionally, we performed a series of comprehensive toxicity evaluations to rule out any potential toxicity from other NBSD derivatives, including NBSD‐NM and NBSD‐NP. None of the three compounds induced hemolysis (Figure S26a, Supporting Information). We then tested the in vivo toxicity of NBSD‐NM, NBSD‐NP, and NBSD‐NOc after a 7‐day tail vein injection. There were no changes in body weight (Figure S26b, Supporting Information) or the weight of organs, including the heart, liver, kidneys, spleen, thymus and lymph nodes (Figure S27, Supporting Information). The blood cell compartment was measured using a hematology analyzer, showing no differences in the numbers of red blood cells, white blood cells, neutrophils, lymphocytes, monocytes, eosinophils, or basophils (Figure S28a, Supporting Information). Additionally, immune cells in the spleen were evaluated via flow cytometry, revealing no changes in F40/80‐positive macrophages, Ly6G‐positive neutrophils, CD220‐positive B cells, CD4‐positive T cells, or CD8‐positive T cells (Figure S28b, Supporting Information). Inflammatory cytokines such as IL‐1β, IFN‐γ, IL‐6, and TNF‐α were not increased, indicating that no inflammatory responses were induced by NBSD‐NM, NBSD‐NP, or NBSD‐NOc (Figure S28c, Supporting Information). Last, we examined histological changes in the heart, liver, and kidney tissues via H&E staining (Figure S29a, Supporting Information). No pathological signs were observed in the heart tissue. Similarly, the liver tissues showed no histological abnormalities, and serum levels of AST and ALT, hepatoxicity markers, remained unchanged (Figure S29b, Supporting Information). No pathological signs or alterations in serum markers of kidney function (creatinine and blood urea nitrogen) were observed (Figure S29c, Supporting Information). Overall, these results indicate that none of the NBSD series pose specific toxicity concerns. In addition, NBSD‐NOc shows potential as a promising post‐surgical implant gel to inhibit tumor recurrence.

## 3 Conclusion

In this study, we successfully developed and characterized the NBSD series (NBSD‐NM, NBSD‐NP, and NBSD‐NOc) with varying alkyl chain lengths (C1, C3, and C8), focusing on their potential as photosensitizers for photodynamic therapy (PDT), particularly in the treatment of colorectal cancer. This work highlights five key points. First, we demonstrated that alkylation of NBSD significantly influences the aggregation behavior, modulating interactions such as π‐π stacking and charge‐charge interactions. Lipidation‐induced aggregation provides a novel approach to control molecular packing and photophysical properties. Second, within the NBSD series, NBSD‐NOc, with the long alkyl chain, exhibited superior photophysical properties, maintaining efficient reactive oxygen species (ROS) generation even in an aggregated state. This overcomes a significant limitation often seen in many conventional photosensitizers. Third, we identified that the NBSD series, particularly NBSD‐NOc, can induce pyroptosis in cancer cells under light irradiation (530 nm, 75 mW cm^2^, 3 min). NBSD‐NOc localized near mitochondria and significantly induced pyroptosis, offering a potential alternative strategy for combating drug‐resistant cancers. Fourth, we developed an implantable hydrogel formulation using an agarose‐based hydrogel incorporating NBSD‐NOc, indicating its potential as a post‐surgical implant to prevent tumor recurrence. The hydrogel exhibited pH‐responsive drug release in the tumor microenvironment, offering a preventive approach against cancer relapse. Notably, the acidic tumor microenvironment (pH 6) effectively triggered the release NBSD‐NOc. Finally, our in vivo studies using a CT26‐xenograft model demonstrated the hydrogel's efficacy in inhibiting tumor recurrence without significant biological toxicity, supporting its potential for clinical translation. These findings highlight the potential of the NBSD series, particularly NBSD‐NOc, as next‐generation photosensitizers. The combination of aggregation‐enhanced PDT effect, high cellular uptake, pyroptosis induction, and implantable properties offers a multifaceted approach to overcoming the limitations of conventional cancer treatments. This work also underscores the importance of molecular design in optimizing photosensitizer performance, demonstrating how tailoring alkyl chain length can balance aggregation patterns and photophysical properties for enhanced therapeutic efficacy. In conclusion, our study presents a promising strategy for post‐surgical management of colorectal cancer by addressing the critical issue of tumor recurrence. The principles and approaches developed in this work may also find broader applications in the treatment of other cancers and the field of nanomedicine. Future studies should focus on further optimizing the NBSD‐NOc hydrogel formulation and conducting extensive preclinical evaluations to pave the way for clinical trials.

## 4 Experimental Section

### General Information

Supporting Information is available on the chemical reagents, instruments, and analytical methods used in this study.

### Solution Test for ^1^O_2_ Detection

UV–vis absorption spectra were recorded using a spectrophotometer (Agilent Technologies Cary 8454, USA). Emission spectra were obtained using a spectrofluorophotometer (SHIMADZU CORP. RF‐6000, Japan) with a 1 cm standard quartz cuvette (internal volume: 0.1 mL, Hellma Analytics, Jena, Germany). The ^1^O_2_ generation ability of the NBSD series (NBSD‐NM, NBSD‐NP, and NBSD‐NOc) was evaluated by monitoring the UV–vis absorption of DPBF (80 µM, ^1^O_2_ capture agent) in EtOH containing 1% DMSO. The solution was irradiated with an LED lamp (530 nm, 75 mW cm^−2^) for 6 min. The absorption spectra were recorded immediately after each irradiation.

### Cell Culture

The human colon cancer cell lines (DLD1, HCT116, HT‐29, LoVo, Ls174t, SW480 and SW620) and mouse colon cancer cell lines (CT26 and MC38) were obtained from the Korean Cell Line Bank. Cells were cultured in Dulbecco's modified Eagle's medium (Hyclone, USA) supplemented with 10% fetal bovine serum (Hyclone, USA) and 1% penicillin‐streptomycin (Gibco, USA). Cultures were maintained at 37 °C in a humidified incubator with 5% CO_2_.

### Photo‐Induced Cytotoxicity Analysis

Cell viability was assessed using a CCK‐8 assay kit (Dojindo Bio, Japan). Briefly, cells were seeded at a density of 4000 cells well^−1^ in a 96‐well plate and incubated for 24 h. They were then treated with NBSD‐NM, NBSD‐NP, or NBSD‐NOc at concentrations of 0, 0.19, 0.39, 0.78, 1.56, 3.125, 6.25, 12.5, and 25 µM. After another 24 h, the treated cells were exposed to light irradiation (530 nm, 75 mW cm^−2^) for 3 min, while the control group was kept in the dark for the same duration. After 24 h, cells were washed with phosphate‐buffered saline (PBS), and CCK‐8 reagent was added to each well. Following a 50 min incubation at 37 °C in 5% CO_2_, absorbance was measured at 450 nm using a microplate reader.

### Western Blot Analysis

CT26 cells were seeded at 300 000 cells per well in a 6‐well plate. The cells were treated with NBSD‐NM, NBSD‐NP, or NBSD‐NOc at IC50 and IC90 concentrations, respectively. MCC950 (10 µM), Z‐DEVD‐FMK (20 µM), or Necrostatin‐1s (10 µM) was pre‐treated for 1 h prior to NBSD exposure (see Supporting Information for detail steps of western blot analysis).

### Immunofluorescence Staining

CT26 cells were treated with NBSD‐NM, NBSD‐NP, or NBSD‐NOc at their respective IC50 concentrations. After 24 h, cells were exposed to light irradiation and fixed 6 h later. Cells were incubated for 24 h overnight at 4 °C with primary antibodies targeting nod‐like receptor protein 3 (NLRP3) (A21906, ABclonal, USA) and apoptosis‐associated speck‐like protein containing CARD (ASC) (ab175449, abcam, USA). Appropriate secondary antibodies (A11008 and ab150131) were used to visualize primary antibodies. Nuclei were counterstained with DAPI (D9542, Sigma, USA) for fluorescence imaging.

### MitoTracker Assay

The co‐localization of NBSD‐NM, NBSD‐NP, and NBSD‐NOc with mitochondria was assessed using BioTracker 405 Blue Mitochondria dye (SCT135, Sigma, USA). CT26 cells were treated with 50 µM of NBSD‐NM, NBSD‐NP, and NBSD‐NOc for 30 min. Then, the cells were stained with BioTracker 405 Blue according to the manufacturer's instructions. Fluorescent signals were visualized using confocal microscopy.

### Animal

BALB/c nude mice (male, 5 weeks old) were obtained from DBL Inc. (Gyeonggi‐do, Rep. of Korea). The mice were randomly divided and housed in cages (20  ×  26  ×  13 cm) with free access to food and water under a 12‐h light/dark cycle (ambient temperature: 23 ± 1 °C, relative humidity: 60 ± 10%). All animal experiments were performed following the National Institute of Health Guide for the Care and Use of Laboratory Animals (NIH publication No. 80‐23, revised 1996) and protocols approved by the Institutional Animal Care and Use Committee of Kyung Hee University (KHUASP‐(SE)‐23‐354) and institutional guidelines (assigned No. 2015–020). The animal experiment involving the tumor mouse model was conducted in accordance with internationally recognized IACUC guidelines and relevant animal experiment regulations.

### CT26‐Xenograft Animal Model

CT26 cells were implanted into BALB/c nude mice (5–6 weeks old) using a Matrigel membrane matrix (No. 354 234, Corning, USA). Each mouse was anesthetized with isoflurane, and a mixture of 1 × 10^7^ cells in fresh DMEM solution and Matrigel (1:1 ratio) was subcutaneously injected into the back of the mouse (100 µL). After implantation, the mice were placed in a temperature‐controlled chamber (30 °C) for recovery and then housed individually in separate cages.

### Evaluation of Tumor Size and Histological Analysis

Tumor size was measured using a caliper, and tumor volume was calculated using the standard formula: Tumor volume = 0.5 × W (width)  ×  D (depth)  ×  H (height). After being harvested, tumor tissues were embedded in OCT compound, and rapidly frozen in isopentane cooled with liquid nitrogen. Frozen tissues were sectioned at 6 µm thickness. Sections were air‐dried and stained with hematoxylin and eosin (H&E) following standard protocols. Images were acquired using a bright‐field microscope.

### Statistical Analysis

All statistical analyses were performed using Prism 8.0 software (GraphPad, La Jolla, CA, USA). Specific statistical methods used are described in the figure captions.

## Conflict of Interest

The authors declare the following competing financial interest(s): They are listed as inventors on a pending patent application related to the technology described in this work.

## Supporting information



Supporting Information

## Data Availability

The data that support the findings of this study are available from the corresponding author upon reasonable request.;
