# Supplementary material for: Programmed Aggregation of Lipidated Nitrobenzoselenadiazole as a Photo‐Activatable Pyroptosis Inducer
Source: Adv Healthc Mater. 2025 Aug 5;14(24):e01567. doi: 10.1002/adhm.202501567 (PMC12447023; doi:10.1002/adhm.202501567)
Supplement: Supplementary file 1 — Supporting Information [file ADHM-14-0-s001.docx]

Supporting Information

Programmed Aggregation of Lipidated Nitrobenzoselenadiazole as a Photo-activatable Pyroptosis Inducer

Jong Min An,^A,1^ Hyunyoung Choi,^B,1^ Hyo In Kim,*^C^ Do-Yeon Kim,^D^ Jahyun Kim,^D^ Jinbong Park,^B^ Junyang Jung,*^A,C,E,F^ Na Young Jeong,*^G^ and Dokyoung Kim*^A,C,D,E,F,H,I^

Corresponding Author:

*Dokyoung Kim, E-mail: dkim@khu.ac.kr; *Na Young Jeong, E-mail: hyukpoka@gmail.com; *Junyang Jung, E-mail: jjung@khu.ac.kr; *Hyo In Kim: hyoin0428@gmail.com

**[Figures]**

Figure S1-2: Transmission electron microscopy (TEM) images

Figure S3-S4: Dynamic Light Scattering (DLS) analysis

Figure S5: Calculation analysis (MM2/MD and Log*P*)

Figure S6: Photophysical properties of the NBSD series in 90% DI water/acetone

Figure S7: Monitoring of the emission wavelength at maximum intensity

Figure S8: Photophysical properties of the NBSD series in 90% DI water/EtOH

Figure S9: Monitoring of the emission wavelength at maximum intensity
Figure S10: 1,3-diphenylisobenzofuran (DPBF) analysis of the NBSD series

Figure S11: Photophysical properties of the NBSD-aniline and 1,3-diphenylisobenzofuran (DPBF) analysis of the NBSD-aniline

Figure S12-S13: Photo-induced cytotoxicity assay of the NBSD series

Figure S14: Time-course analysis for the uptake rate of the NBSD series

Figure S15: Cellular uptake rate of the NBSD series in various cell lines

Figure S16: Cellular uptake measurement by absorbance

Figure S17: Live/dead cell assays in CT26 cells

Figure S18: Western blot analysis with pyroptosis, apoptosis, and necroptosis inhibitors

Figure S19: Membrane budding and rupture analysis

Figure S20: Western blot analysis for the pyroptosis mechanism

Figure S21: Analysis of key markers (IL-1β and IL-18) for pyroptosis

Figure S22: M1 macrophage polaryzation assessment

Figure S23: Measurement of total ROS, superoxide anion, hydroxyl radical, and singlet oxygen

Figure S24: Computational pharmacokinetic profiles

Figure S25: Histological analysis of tumor tissues

Figure S26: Hemolysis assay

Figure S27-29: In vivo toxicty assay

**[Tables]**
Table S1: DLS analysis

Table S2-S16: Crystal data

Table S17-S22: Raw data for monitoring tumor size

1. Experimental section

- 1. General information

Chemical reagents were purchased from Sigma-Aldrich (St. Louis, MO, USA), Alfa Aesar (Haverhill, MA, USA), and TCI (Tokyo, Japan). Commercially available reagents and anhydrous solvents were used without further purification. 3-fluorobenzene-1,2-diamine was obtained from Apollo Scientific Ltd. (UK). Selenium dioxide (SeO_2_) and 1,3-Diphenylisobenzofuran (DPBF) were sourced from Sigma-Aldrich (USA). Nitric acid (HNO_3_) and sulfuric acid (H_2_SO_4_) were acquired from Alfa Aesar (USA). Anhydrous alkyl amines were obtained from Sigma-Aldrich (USA). Dimethyl sulfoxide (DMSO, anhydrous) was purchased from MilliporeSigma Inc. (USA). Chloroform-d and Dimethyl sulfoxide-d_6_ (D, 99.9%) were obtained from Cambridge Isotope Laboratories Inc. (USA). The pH buffers (pH 6 and pH 7.4) were sourced from Daejung Chemicals & Metals Co., Ltd. (Rep. of Korea). For cell culture experiments, cell culture dishes (SPL Life Science, #20060, Rep. of Korea) were used. Thin-layer chromatography (TLC) was performed on pre-coated silica gel 60F-254 glass plates (Merck KGaA, Germany). Single-crystal X-ray crystallography was performed at the Korea Basic Science Institute (KBSI) Western Seoul Center (Rep. of Korea) using an Agilent SuperNova X-ray diffractometer (USA). ^1^H-NMR and ^13^C-NMR spectra were recorded using Bruker AVANCE III 500 MHz (USA). In the NMR spectra, chemical shifts (δ) are reported in ppm, with multiplicities indicated as s (singlet), d (doublet), t (triplet), dd (double of doublets), and m (multiplet). ^1^H-NMR Spectra were referenced to residual CDCl_3_ (7.26 ppm). ^13^C-NMR Spectra were referenced to residual DMSO-*d*_6_ (39.51 ppm) and CDCl_3_ (77.5 ppm). Transmission electron microscopy (TEM) images were acquired using a Tecnai G2 F30ST (FEI Company, OR, USA) at the Korea Basic Science Center (KBSI, Korea University, Seoul, Rep. of Korea).

**1.2. Synthesis of NBSD series**

**[Synthesis of 4-fluoro-nitrobenzoselenadiazole]** The reaction was performed according to our previous report. Briefly, 3-fluorobenzene-1,2-diamine (0.3964 mmol) and selenium dioxide (SeO_2_, 0.4757 mmol) were dissolved in ethanol. The mixture was slowly heated to 80 °C under reflux conditions for 1 h. The reaction was monitored using TLC. After the reaction, the mixture was cooled to 25 °C. The cooled mixture was then separated into organic and aqueous layers: solvent ethyl acetate (EA) for the organic layer and deionized water (DI. H_2_O) for the aqueous layer. The organic layer was rinsed with DI. H_2_O three times. The drying process was achieved by adding anhydrous sodium sulfate (Na_2_SO_4_) at 25 °C for 30 min. The solvent was evaporated under reduced pressure. The mixture was then purified by flash silica gel column chromatography (eluent: EA/*n*-hex= 1/9, *v*/*v*), yielding the expected product (90% yield, white powder). ^1^H-NMR (500 MHz, CDCl_3_); δ 7.66 (d, *J* = 9.1 Hz, 1H), 7.44 (ddd, *J* = 9.1, 7.3, 5.2 Hz, 1H), 7.08 (ddd, *J* = 10.1, 7.3, 0.8 Hz, 1H). To synthesize 4-fluoro-nitrobenzoselenadiazole, 4-fluoro-benzoselenadiazole was dissolved in concentrated sulfuric acid (conc. H_2_SO_4_, 0.003 mmol), followed by the dropwise addition of concentrated nitric acid (conc. HNO_3_, 3.730 mmol). The reaction was initially carried out in a round-bottom flask for 30 min, with the temperature rising from 0 °C to 25 °C. Afterwards, cold DI. H_2_O (10 mL) was added to quench the reaction, and the mixture was left for 10 min at 25 °C. Upon the appearance of a yellowish precipitate, the mixture was filtered through Whatman® Grade 1 filter paper (USA), yielding a yellow powder. The powder was then concentrated under a vacuum. The expected product was obtained with an isolated yield of 89%. ^1^H-NMR (500 MHz, DMSO-*d*_6_) δ 8.60 (dd, *J* = 8.3, 4.6 Hz, 1H), 7.28 (t, *J* = 8.4 Hz, 1H).

**[Synthesis of NBSD-aniline]** 4-fluoro-nitrobenzoselenidiazole (1.2 mmol) was dissolved in 7 mL of *N*,*N*-dimethylformamide (DMF), followed by the addition of the aniline (4.8 mmol, 3.97 eq) and triethylamine (2.4 mmol, 1.97 eq). After 40 min, starting molecule as a 4-fluoro-nitrobenzoselenidiazole was disappeared on TLC while the expected product was observed in an rf value of 0.15 in a developing solution of 20% EA/n-Hex with three time development. After the reaction was complete, the precipitate was filtered and washed with water three times to remove any unreacted aniline. The desired product was analyzed by ^1^H-NMR (500 MHz, DMSO-*d*_6_) δ 8.57 (d, J = 8.9 Hz, 1H), 7.53 – 7.46 (m, 4H), 7.27 (tt, J = 6.9, 1.8 Hz, 1H), 6.76 (d, J = 8.9 Hz, 1H). Found mass: [M+H]^+^ 320.86

**[Synthesis of NBSD-NM, NBSD-NP, and NBSD-NOc]** 4-fluoro-nitrobenzoselenidiazole (0.2 mmol) was dissolved in 2.66 mL of MeCN, followed by the addition of the corresponding alkyl amines (0.8 mmol, 4 eq). After 15 min, precipitation (orange in color) occurred. The reaction was monitored using a TLC plate, with an rf value of 0.15 in a developing solution of 50% EA/HEX. After the reaction was complete, the precipitate was filtered and washed with water three times to remove any unreacted alkyl amines. The desired product was obtained with an isolated yield of 68.9% (NBSD-NM), 81.2% (NBSD-NP), and 82.6% (NBSD-NOc).

[*NBSD-NM*]: ^1^H-NMR (500 MHz, CDCl_3_) δ 8.72 (d, *J* = 8.7 Hz, 1H), 6.22 (dd, *J* = 8.8 Hz, 1H), 3.9 (s, 3H). ^13^C NMR (101 MHz, DMSO) δ 151.92 (d, *J* = 11.8 Hz), 149.28, 135.33, 97.09, 29.89.

[*NBSD-NP*]: ^1^H-NMR (500 MHz, CDCl_3_) δ 8.70 (d, *J* = 8.8 Hz, 1H), 6.22 (dd, *J* = 8.8 Hz, 1H), 3.43 (t, *J*= 6.9 Hz, 2H), 1.88 – 1.83 (m, 2H), 1.13 – 1.09 (m, 3H). ^13^C NMR (126 MHz, CHLOROFORM-*D*) δ 153.06, 152.85, 147.91, 136.10, 130.29, 97.66, 45.67, 22.50, 11.99.

[*NBSD-NOc*]: ^1^H-NMR (500 MHz, CDCl_3_) δ 8.69 (d, *J* = 8.8 Hz, 1H), 6.21 (dd, *J* = 8.8 Hz, 1H), 3.45 (t, *J*= 7.2 Hz, 2H), 1.80 (dq, *J*= 12.1, 5.9, 4.5 Hz, 2H), 1.40 – 1.26 (m, 10H), 0.89 (t, *J*= 6.9 Hz, 3H). ^13^C NMR (126 MHz, CHLOROFORM-*D*) δ 152.98, 152.80, 147.91, 136.11, 130.14, 97.61, 44.02, 32.18, 29.66, 29.59, 29.12, 27.46, 23.06, 14.53.

**1.3. Western blot analysis**

CT26 cells were seeded at 300,000 cells per well in a 6-well plate. The cells were treated with NBSD-NM, NBSD-NP, or NBSD-NOc at IC50 and IC90 concentrations, respectively. MCC950 (10 μM), Z-DEVD-FMK (20 μM), or Necrostatin-1s (10 μM) was pre-treated for 1 h prior to NBSD exposure. After 3 h of treatment, the cells were exposed to laser treatment, and proteins were extracted 3 h later using a lysis buffer containing protease and phosphatase inhibitors. A total of 30 µg of protein was loaded onto a gel for electrophoresis, followed by protein transfer onto a nitrocellulose membrane using the wet transfer method. The membrane was blocked with 5% non-fat dry milk in tris-buffered saline containing 0.1% Tween-20 (TBST) for 1 h and then incubated overnight at 4 °C with primary antibodies against gasdermin D (GSDMD), interleukin-1 beta (IL-1β), pro-caspase-1 (CST-24232T), and glyceraldehyde-3-phosphate dehydrogenase (GAPDH) (CST-2118s). After incubation with the appropriate secondary antibody for 1 h, protein detection was performed using an enhanced chemiluminescence substrate. The band intensities of GAPDH for GSDMD were 100.00, 102.04, 114.33, 129.06, 92.30, 67.72 in lane 1-6, respectively. The band intensities of GAPDH for IL-1β were 100.00, 91.79, 86.28, 74.45, 65.09, 60.85 in lane 1-6, respectively. The band intensities of GAPDH for Caspase-1 100.00, 69.63, 71.27, 59.52, 80.70, 76.09 in lane 1-6, respectively.

**1.4. Measurement of cellular uptake of NBSD series**

To evaluate time-based cellular uptake of NBSD derivatives, CT26 cells were treated with 20 μM of NBSD-NM, NBSD-NP, or NBSD-NOc. Time-dependent fluorescence signals were observed at 15 min, 30 min, 1 h, 3 h, 6 h, 9 h, and 24 h after treatment. To determine cellular uptake of NBSD derivatives in various colon cancer cell lines, DLD1, CT26, HCT116, HT29, LoVo, LS174T, MC38, and SW620 cells were treated with 20 μM of NBSD-NM, NBSD-NP, or NBSD-NOc for 24 h. To quantify cellular uptake of NBSD derivatives, CT26 cells were treated with NBSD-NM, NBSD-NP, or NBSD-NOc at their respective IC_50_ concentrations. Then, at 4, 8, and 24 h post-treatment, culture supernatants were collected and their absorbance spectra were recorded over the defined wavelength range using a spectrophotometer. The area under the absorbance curve (AUC) was calculated for each time point. Drug remaining in the supernatant was expressed as the percentage of the initial AUC. The decrease in AUC was interpreted as the fraction of drug taken up by the cells.

**1.5. Live-cell imaging of NBSD series-induced morphological changes**

CT26 cells were plated onto glass-bottom confocal dishes and treated with NBSD, NBSD-NM, NBSD-NP, or NBSD-NOc at their respective IC_50_ concentrations at 24 h, followed by 3 min of light irradiation. Live-cell imaging was performed at 2 h and 4 h post-treatment using a confocal microscope equipped with a phase-contrast module and a 37 °C, 5% CO_2_ incubation chamber. Membrane budding and rupture (pyroptotic body formation) were indicated with red and blue arrows.

**1.6. Live/dead cell imaging and flow cytometry**

CT26 cells were treated with NBSD, NBSD-NM, NBSD-NP, or NBSD-NOc at their respective IC_50_ concentrations for 24 h, followed by 3 min of light irradiation. Cell viability was assessed using the LIVE/DEAD Cell Imaging Kit (Thermo Fisher, Cat# R37601, USA) according to the manufacturer's instructions. Stained cells were imaged using a confocal microscope equipped with GFP (Ex/Em: 488/515 nm) and Texas Red (Ex/Em: 570/602 nm) filters. Green fluorescence indicates live cells and red fluorescence indicates dead cells. For the flow cytometry, CT26 cells were harvested after the same treatment, washed with cold PBS, and stained using an Annexin V-FITC / 7-AAD Apoptosis Detection Kit (e.g., BioLegend, Cat# 640922, USA) according to the manufacturer’s protocol. Samples were analyzed on a Beckman Coulter CytoFLEX flow cytometer using FITC and 7-AAD channels. Data were processed and quantified using FlowJo v10 software with standard quadrant gating.

**1.7. Macrophage activation assay and cytokine quantification**

CT26 murine colon carcinoma cells were treated with NBSD-NM, NBSD-NP, or NBSD-NOc at their respective IC₅₀ concentrations for 24 h, followed by 3 min of light irradiation to activate PDT. Supernatants were collected and centrifuged to remove cell debris, and then transferred to naïve RAW264.7 macrophages for an additional 24 h. M1 polarization was assessed by western blotting for CD86 and TNF-α, normalized to GAPDH. IFN-γ levels in RAW264.7 supernatants were measured by ELISA (BD OptEIA™, Cat# 555138), and nitric oxide production was quantified by Griess assay (Invitrogen, Cat# G7921, USA) via absorbance at 540 nm.

**1.8. ROS species quantification**

CT26 cells were treated with NBSD-NM, NBSD-NP, or NBSD-NOc at their IC₅₀ concentrations for 24 h, followed by 3 min of light irradiation. Total ROS and superoxide in the medium were quantified using the ROS/Superoxide Detection Kit (Abcam, Cat# ab139476, USA), and fluorescence was measured at 488/520 nm (total ROS) and 550/620 nm (superoxide) using a microplate reader. Hydroxyl radical production was assessed from cell lysates using the Hydroxyl Radical Assay Kit (ELK Biotechnology, China) according to the manufacturer’s instructions. Lysates were incubated at 37 °C for 1 min, and absorbance was measured at 510 nm. Hydroxyl radical capacity was calculated using the kit-provided formula.

**1.9. Singlet Oxygen Detection**

CT26 cells were incubated with NBSD, NBSD-NM, NBSD-NP, or NBSD-NOc at their respective IC₅₀ concentrations for 24 h, followed by the addition of Singlet Oxygen Sensor Green (SOSG; Invitrogen, cat# S36002). After probe loading, cells were exposed to visible light for 3 min to activate the compounds. Live-cell fluorescence imaging was performed immediately after irradiation using a confocal microscope with FITC channel settings (Ex/Em: ~504/525 nm). Green fluorescence indicates intracellular singlet oxygen production.

**1.10. In vivo toxicity analysis**

Fifteen Balb/c mice were randomly divided into three groups as follows: a) PBS-injected control group (n = 4), b) NBSD-NM-injected group (n = 4), c) NBSD-NP-injected group (n = 4), and d) NBSD-NOc-injected group (n = 4). NBSD-NM, NBSD-NP, or NBSD-NOc (7.4 mg/kg of body weight, dissolved in 100 μL PBS) were injected into the tail vein for 10 consecutive days. An equal volume of PBS was administered to the control group. 2 h after the last injection, blood was collected via cardiac puncture, and each organ (heart, liver, kidney, spleen, thymus, and lymph node) was harvested. Changes in body weight, food intake, and water intake of the mice were observed daily.

**1.11. Hematology analysis**

A hemolysis assay was performed on NBSD-NM, NBSD-NP, and NBSD-NOc. Fresh blood, anticoagulated with 2 µL heparin per 1 mL, was diluted tenfold in PBS. A 90 µL aliquot of the diluted blood was mixed with 10 µL of each test sample or control (blank or 0.2% Triton X-100) in microfuge tubes. After 10 min at 37 °C, the samples were centrifuged at 10,000 × g for 5 min. The supernatants were transferred to a 96-well plate, and absorbance was measured at 350 nm. Percent hemolysis was calculated as follows:

$$\%Hemolysis=\frac{OD of sample}{OD of Triton}\times100$$

**1.12. Hematology analysis**

Freshly isolated blood from the mice was analyzed using the Drew Scientific Hemavet 950 Hematology Analyzer System (Erba Diagnostics, Oxford, CT, USA) to determine the blood cell composition.

**1.13. Flow cytometry analysis**

Flow cytometry analysis was performed on splenocytes to determine immune cell compositions. Briefly, the harvested spleens were meshed using a 70 μm cell strainer and collected in 10 ml of 1× PBS. After removing red blood cells (RBCs) with ACK Lysing Buffer (Thermo-Fisher, USA) for 5 min, the cells were collected by centrifugation (200 g, 10 min). The cells were then suspended in 1× PBS and stained with anti-mouse antibodies as follows: a) APC-F4/80 and PE-CD11b for macrophages, b) APC-Ly6G and PE-CD11b for neutrophils, c) PE-CD220 for B cells, d) FITC-CD4 for CD4^+^ T cells, or e) PC7-CD8 for CD8^+^ T cells, for 15 min at room temperature (1:200-400 in PBS). All antibodies were purchased from BioLegend (San Diego, CA, USA). Flow cytometry was then performed using a CytoFLEX (Beckman Coulter, USA).

**1.14. Enzyme-linked immunosorbent assay (ELISA)**

Blood was collected via cardiac puncture, allowed to clot for 30 min at room temperature, and then centrifuged at 2,000g for 10 min at 4°C to obtain serum. The serum was stored at −80 °C until use. Plasma levels of aspartate aminotransferase (AST), alanine aminotransferase (ALT), creatinine, and blood urea nitrogen (BUN) were measured using commercial ELISA kits (MyBioSource, San Diego, CA, USA) according to the manufacturer’s instructions. Levels of IL-1β and IL-18 were measured using ELISA kits purchased from R&D Systems (Minneapolis, MN, USA).

**1.15. Histology of the heart, liver, and kidney**

Hematoxylin and eosin (H&E) staining was performed on heart, liver, and kidney tissues. The tissues were fixed in 10% formalin for 48 h at room temperature and then transferred to 70% ethanol for overnight storage at 4°C. After fixation, the tissues underwent dehydration, clearing, and infiltration. The samples were then embedded in paraffin and sectioned into 5 μm-thick slices using a microtome (Leica, Nussloch, Germany) to prepare tissue slides. For H&E staining, the slides were first deparaffinized, followed by staining with hematoxylin and eosin. The slides were then dehydrated, cleared, and mounted before being examined under a light microscope.

**[Supporting Figures]**

**
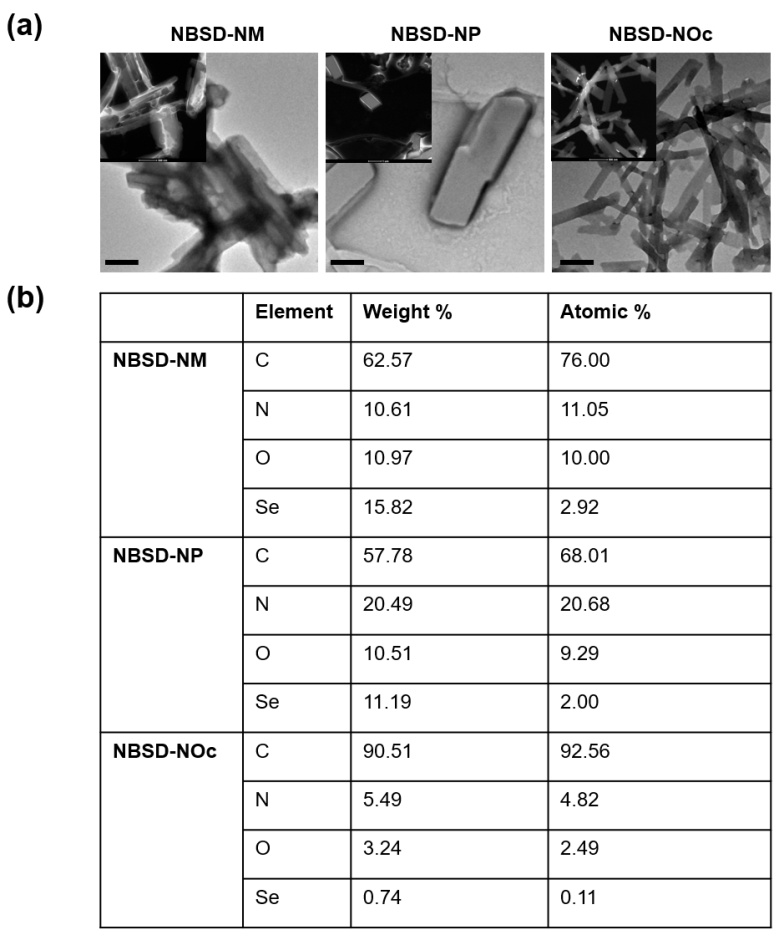
**

**Figure S1.** (a) Transmission electron microscopy (TEM) images of the NBSD series. (b) Elemental analysis of the NBSD series. NBSD-NM (Atomic, %): C, 76.00; N, 11.05; O, 10.00; Se, 2.92. NBSD-NP (Atomic, %): C, 68.01; N, 20.68; O, 9.29; Se, 2.00. NBSD-NOc (Atomic, %): C, 92.56; N, 4.82; O, 2.49; Se, 0.11.


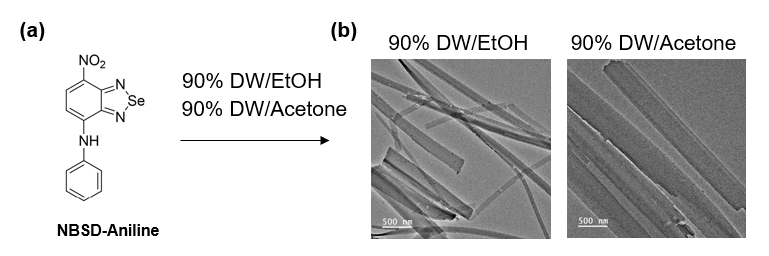


**Figure S2.** (a) Chemical structure of NBSD-aniline. (b) Transmission electron microscopy (TEM) images of the NBSD-aniline (100 µM) in 90% DI water (10% EtOH or 10% acetone).

**
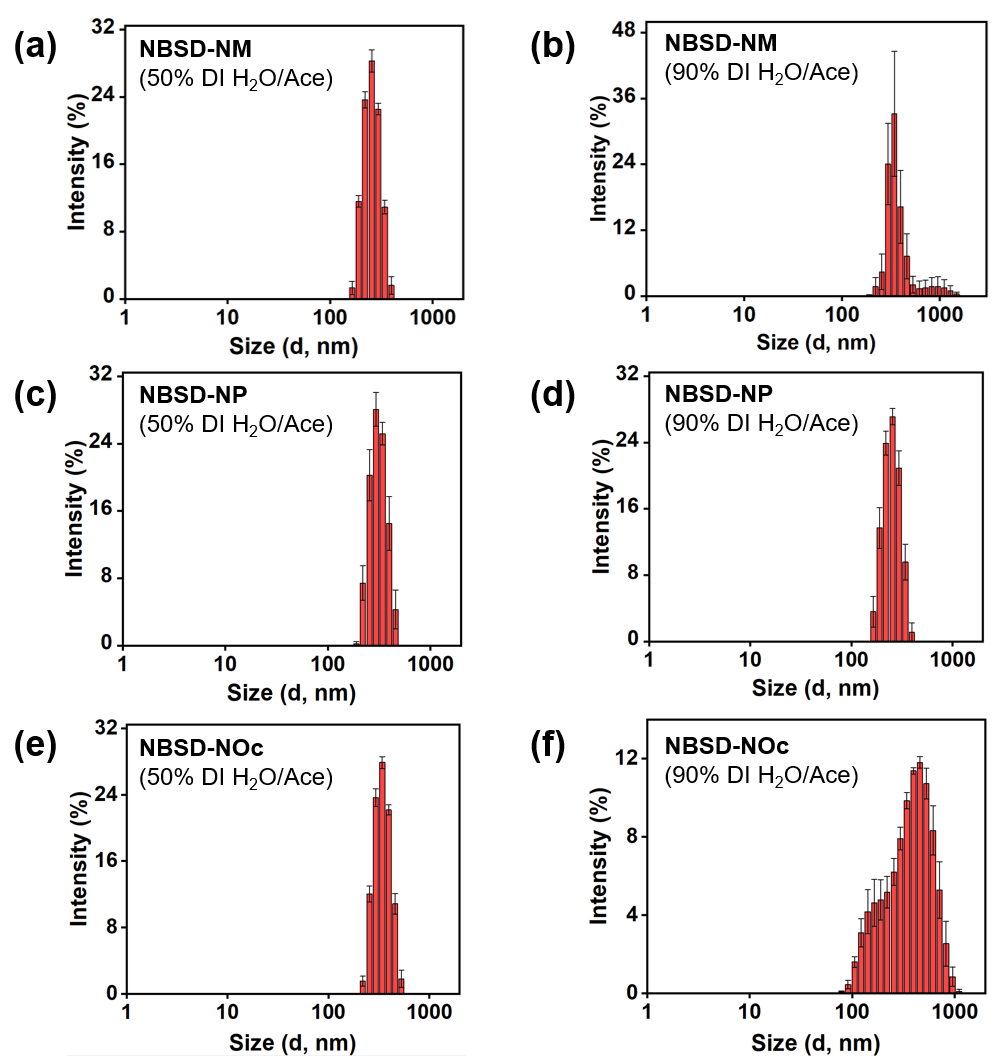
**

**Figure S3.** Dynamic Light Scattering (DLS) analysis of the NBSD series in 50% and 90% DI. H_2_O/acetone mixtures. (a) NBSD-NM in 50% DI. H_2_O/acetone. (b) NBSD-NM in 90% DI. H_2_O/acetone. (c) NBSD-NP in 50% DI. H_2_O/acetone. (d) NBSD-NP in 90% DI. H_2_O/acetone. (e) NBSD-NOc in 50% DI.H_2_O/acetone. (f) NBSD-NOc in 90% DI.H_2_O/acetone. The aggregate size and Polydisperity Index (PDI) values are presented in Table S1.


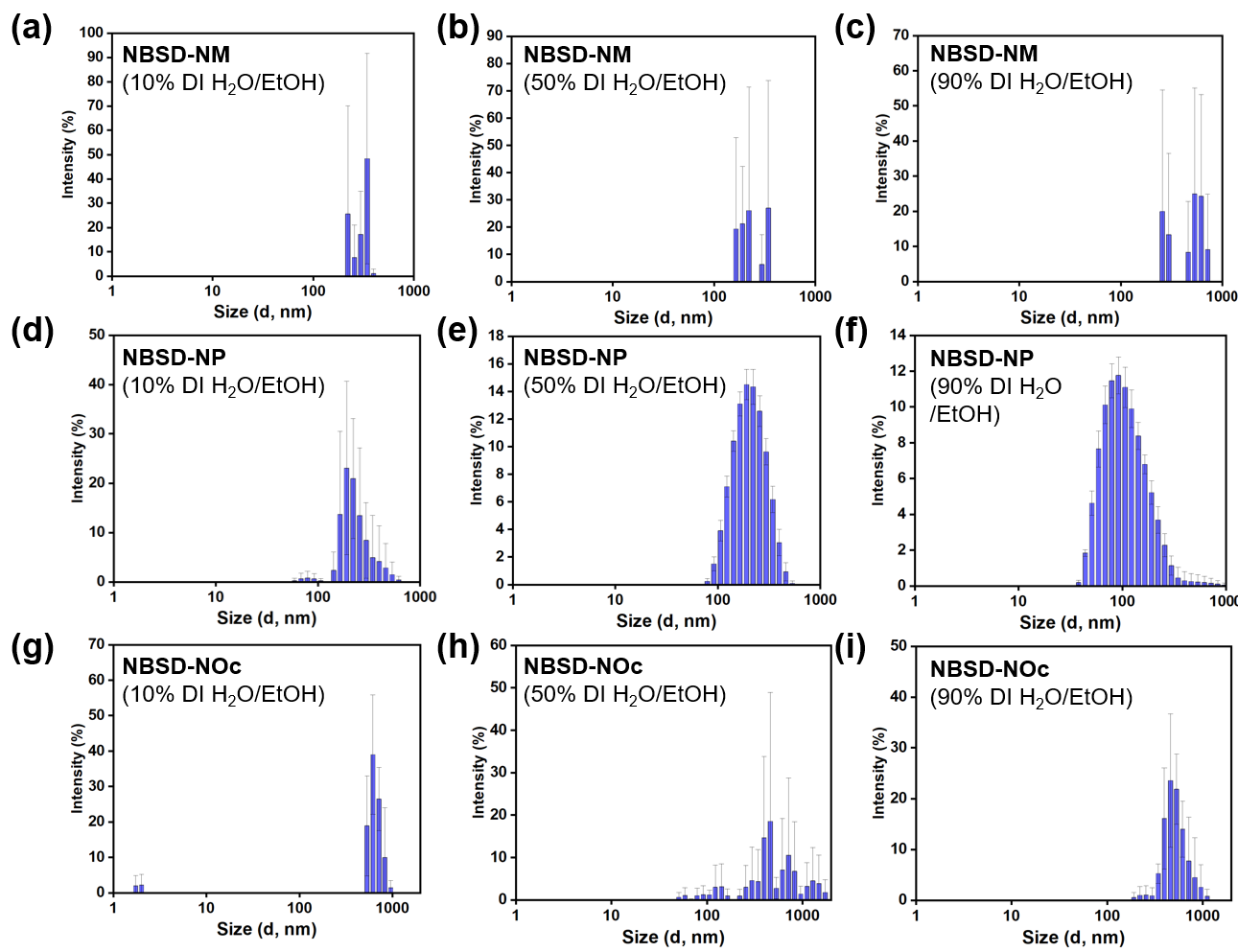


**Figure S4.** DLS analysis of the NBSD series in 10%, 50%, and 90% DI. H_2_O/EtOH mixtures. (a) NBSD-NM in 10% DI. H_2_O/acetone. (b) NBSD-NM in 50% DI. H_2_O/acetone. (c) NBSD-NM in 90% DI. H_2_O/acetone. (d) NBSD-NP in 10% DI.H_2_O/acetone. (e) NBSD-NP in 50% DI. H_2_O/acetone. (f) NBSD-NP in 90% DI. H_2_O/acetone. (g) NBSD-NOc in 10% DI. H_2_O/acetone. (h) NBSD-NOc in 50% DI.H_2_O/acetone. (i) NBSD-NOc in 90% DI. H_2_O/acetone. The aggregate size and PDI values are presented in Table S1.

**
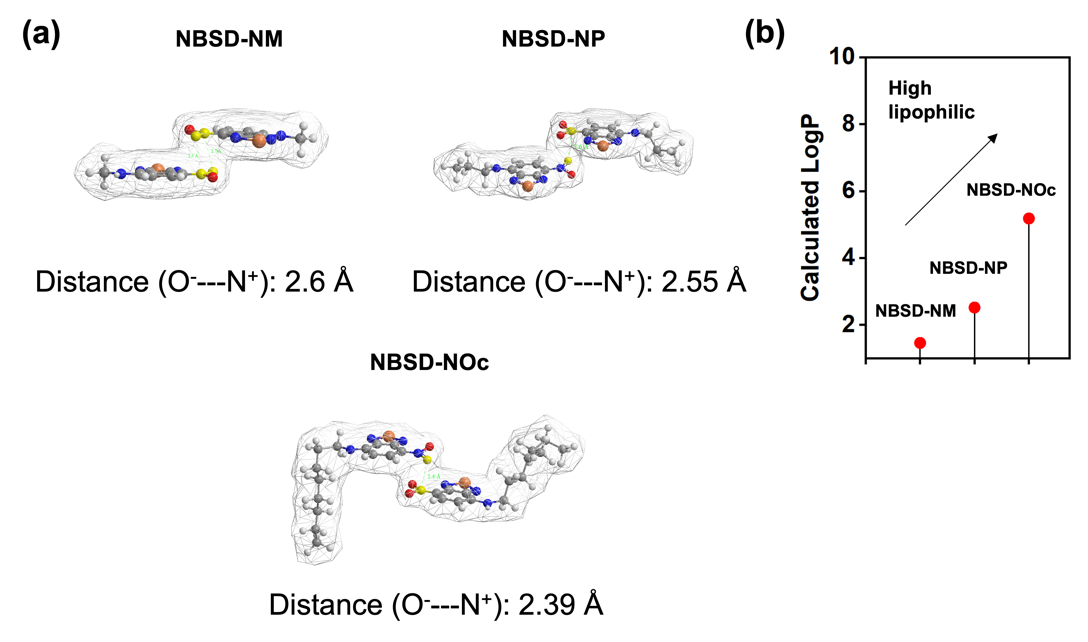
**

**Figure S5.** (a) MM2/MD calculations for the NBSD series. Step interval: 2 fs, frame interval: 10 fs, termination step: 40,000, heating/cooling rate: 1.0 Kcal/atom/ps, temperature: 300 Kelvin. (b) Log*P* calculations for the NBSD series using ChemSkech (ACDLabs Freeware).

**
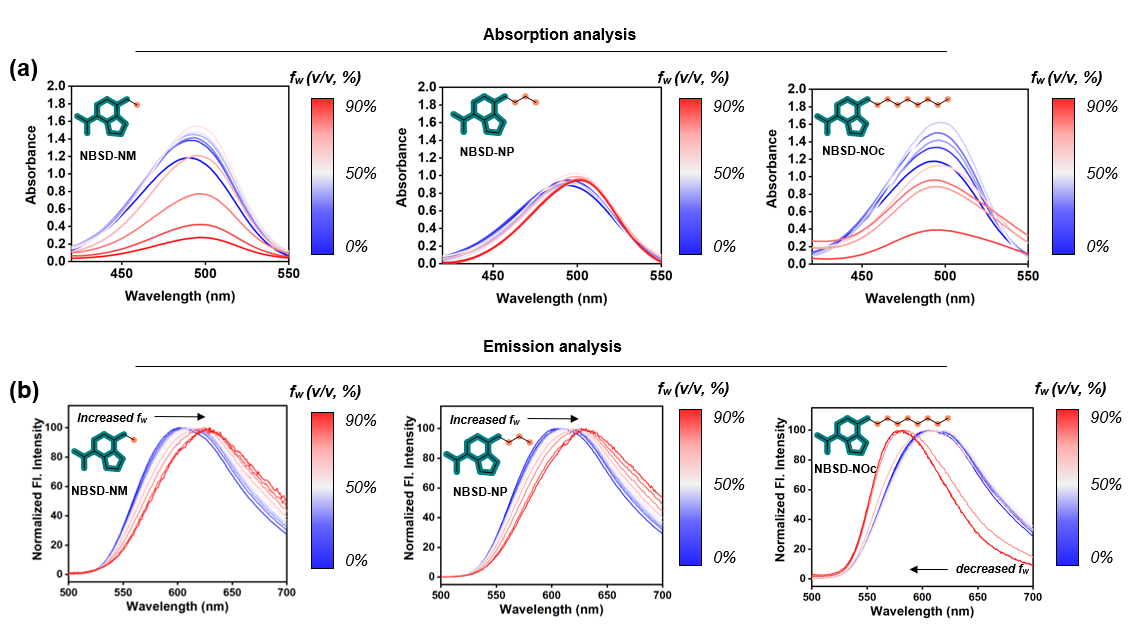
**

**Figure S6.** Photophysical properties of the NBSD series. (a) Absorption spectra of NBSD-NM, NBSD-NP, and NBSD-NOc in EtOH/DI. H_2_O (0% EtOH to 90% EtOH). (b) Emission spectra of NBSD-NM, NBSD-NP, and NBSD-NOc in EtOH/DI. H_2_O (0% EtOH to 90% EtOH). The concentration was fixed at 100 µM. Emission spectra were obtained after excitation at 490 nm.


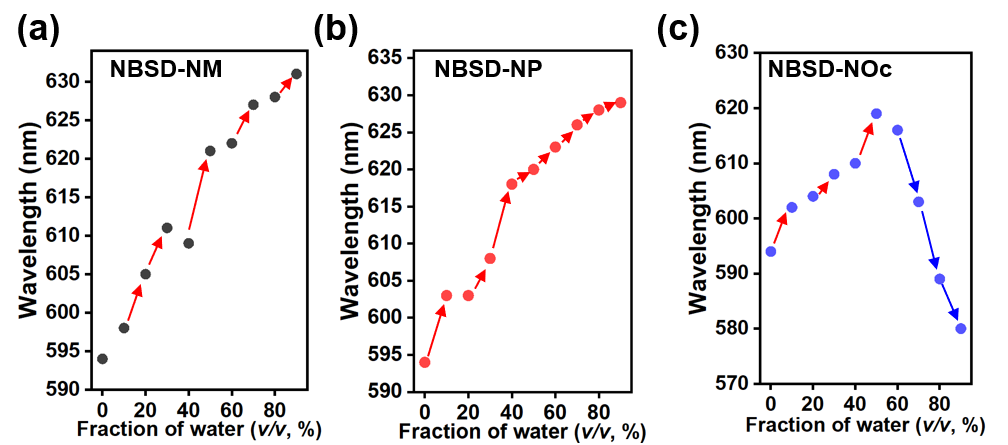


**Figure S7.** Monitoring of the emission wavelength at maximum intensity. (a) Shift in the emission wavelength of NBSD-NM in acetone/DI. H_2_O (0% acetone to 90% acetone). (b) Shift in the emission wavelength of NBSD-NP in acetone/DI. H_2_O (0% acetone to 90% acetone). (c) Shift in the emission wavelength of NBSD-NOc in acetone/DI. H_2_O (0% acetone to 90% acetone). The concentration was fixed at 100 µM. Emission spectra were obtained after excitation at 490 nm.


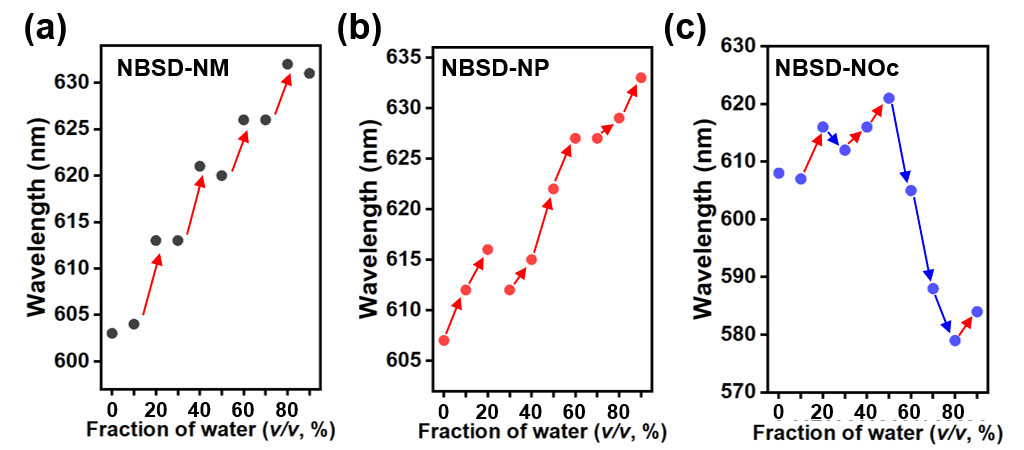


**Figure S8.** Monitoring of the emission wavelength at maximum intensity. (a) Shift in the emission wavelength of NBSD-NM in EtOH/DI. H_2_O (0% EtOH to 90% EtOH). (b) Shift in the emission wavelength of NBSD-NP in EtOH/DI. H_2_O (0% EtOH to 90% EtOH). (c) Shift in the emission wavelength of NBSD-NOc in acetone/DI. H_2_O (0% EtOH to 90% EtOH). The concentration was fixed at 100 µM. Emission spectra were obtained after excitation at 490 nm.


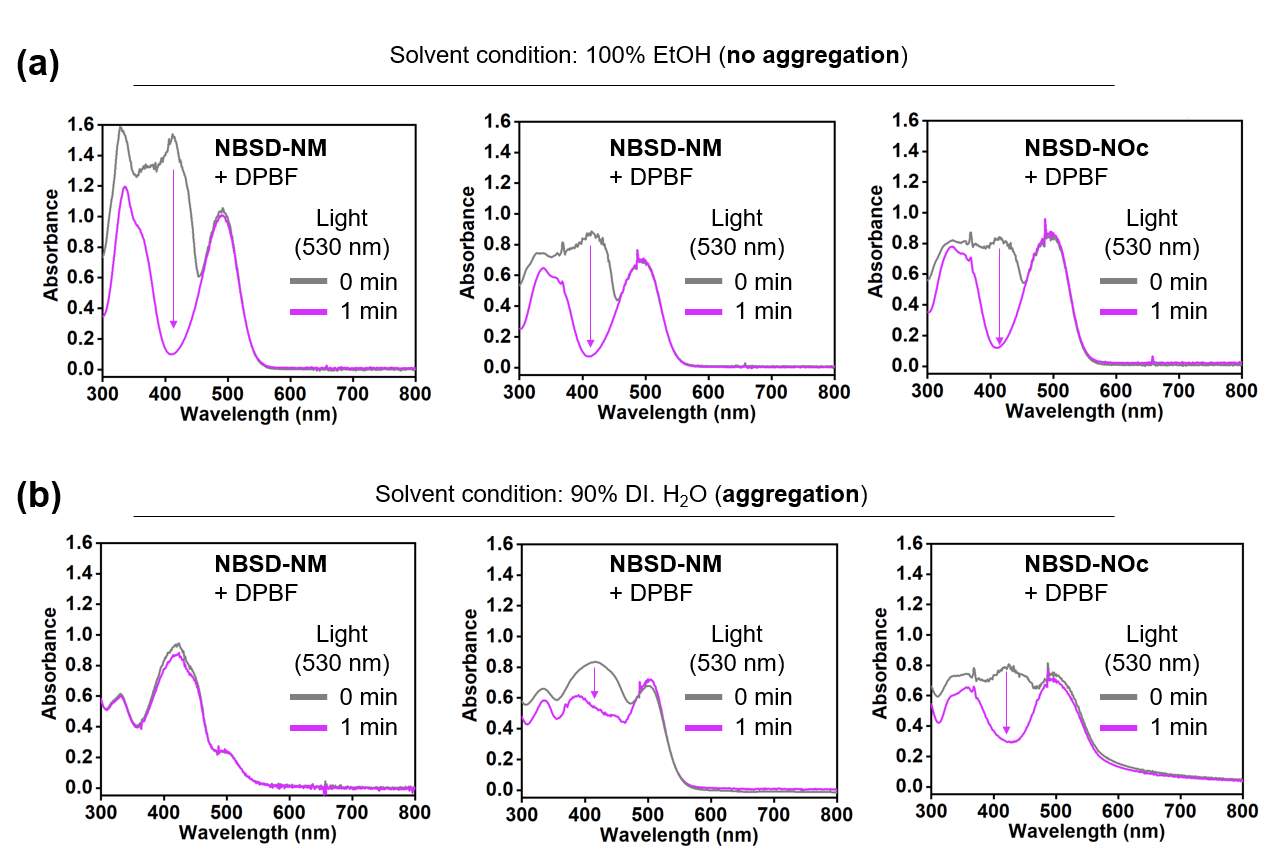


**Figure S9.** 1,3-diphenylisobenzofuran (DPBF) analysis of the NBSD series in (a) EtOH and (e) 90% DI. H_2_O in EtOH under light irradiation (530 nm, 75 mW/cm^2^). Purple arrow: decreased DPBF signal. The concentration of the NBSD series was fixed at 100 µM.


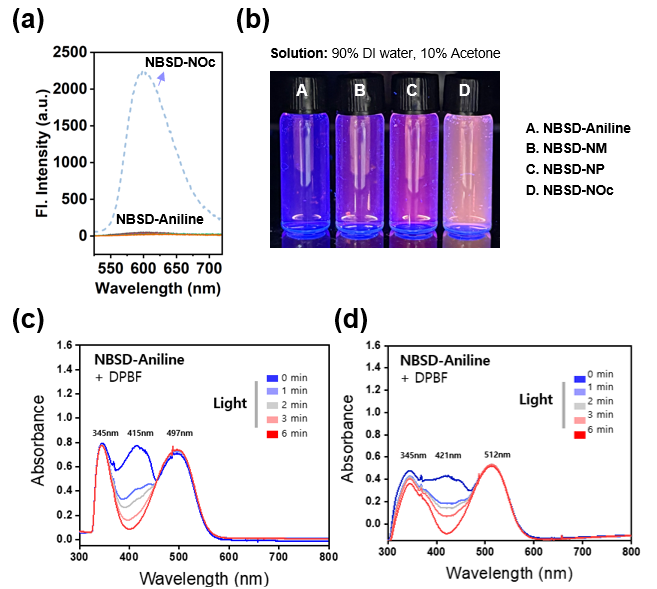


**Figure S10.** (a) Emission spectra of NBSD-aniline and NBSD-NOc. The NBSD-aniline (100 µM) was analyzed in different solution condition (0 to 90% DI water in acetone). The NBSD-NOc (100 µM, 90% DI water/acetone) was control group to compare the fluorescence intensity between NBSD-NOc and NBSD-aniline. (b) Images of NBSD-aniline, NBSD-NM, NBSD-NP, and NBSD-NOc in UV irradiation box (365 nm). The concentration for each compounds was fixed at 50 µM in 90% DI water/acetone. 1,3-diphenylisobenzofuran (DPBF) analysis of the NBSD-aniline in (c) acetone and (e) 90% DI. H_2_O in acetone under light irradiation (530 nm, 75 mW/cm^2^). The concentration of the NBSD series was fixed at 100 µM.


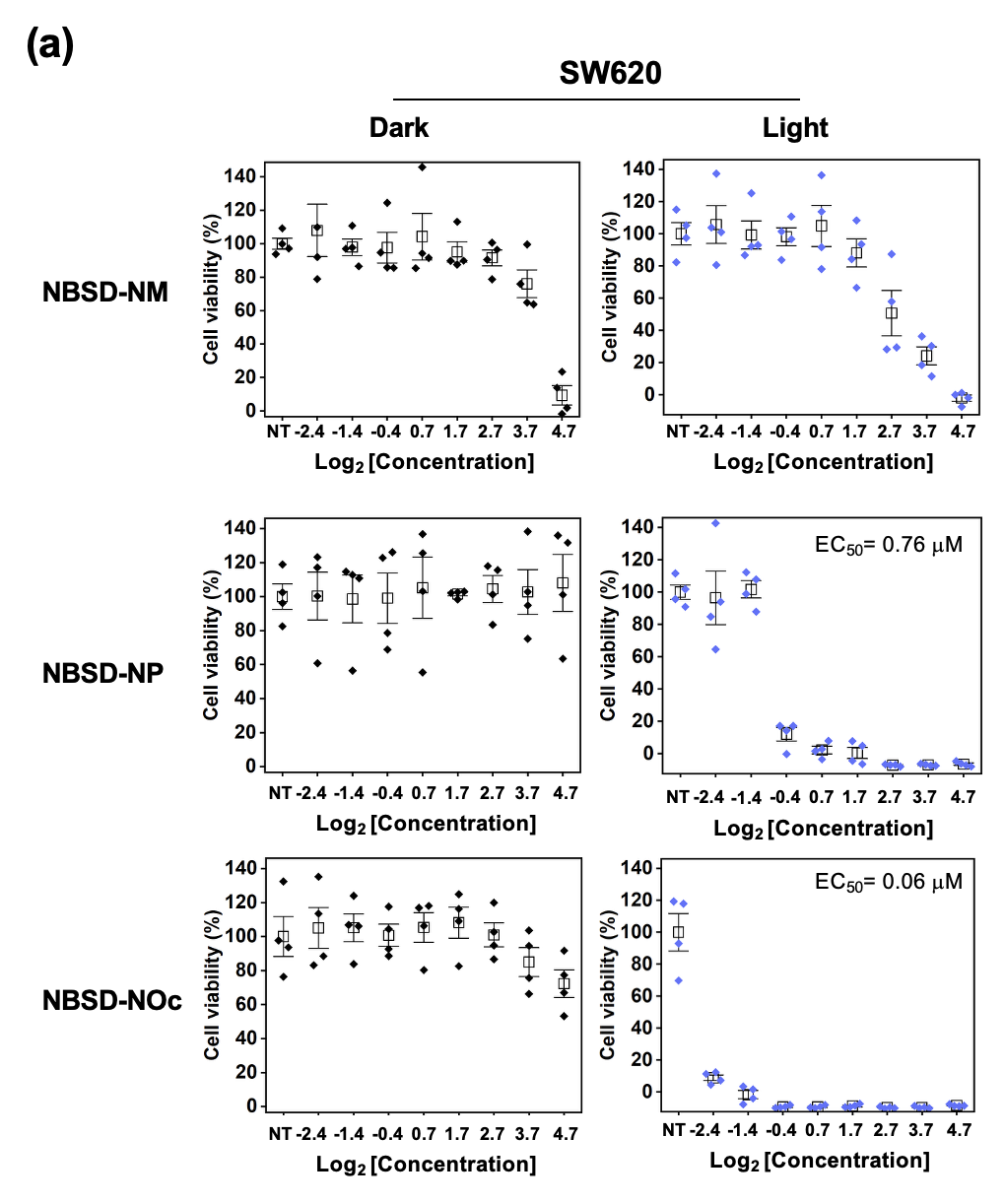

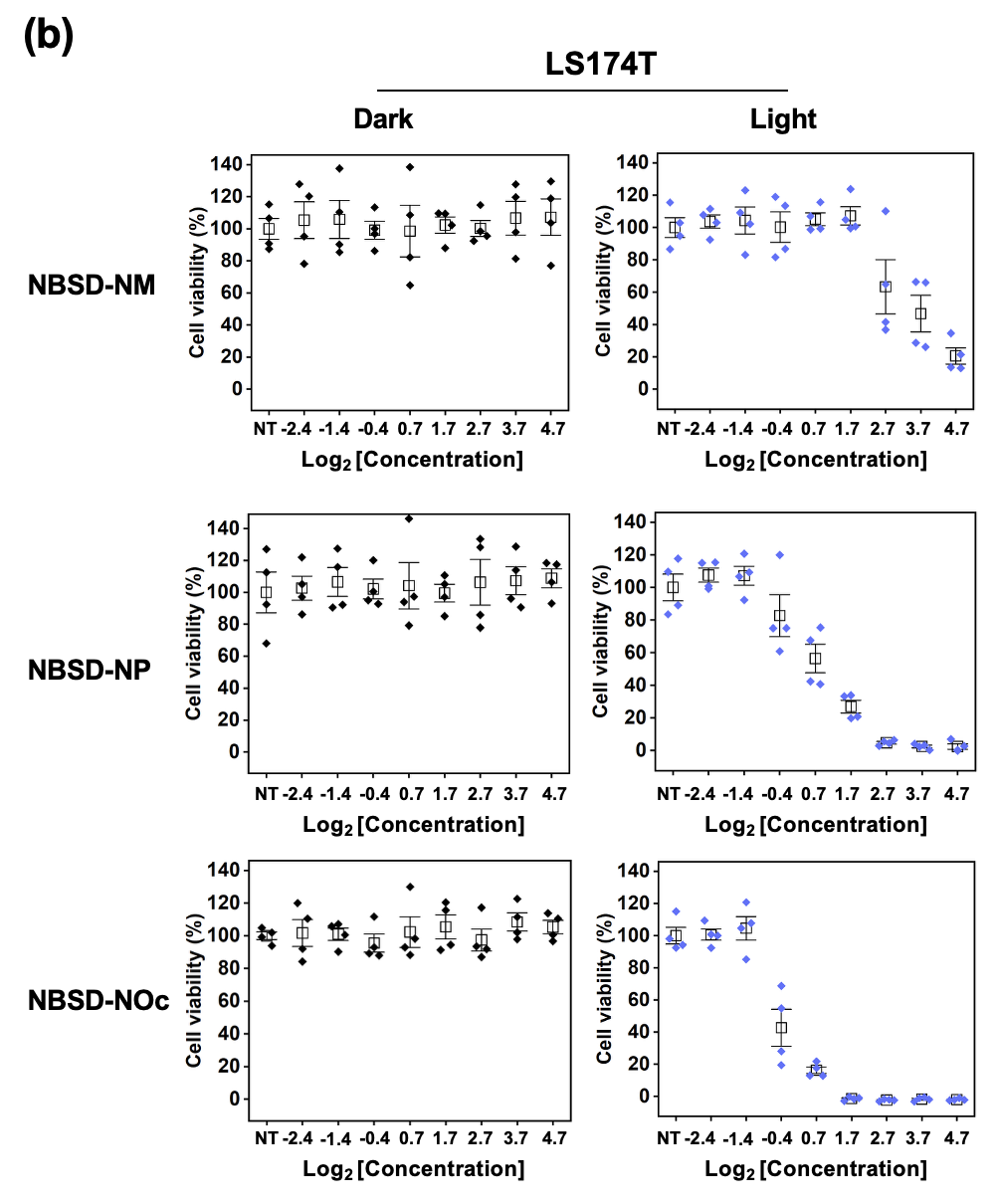

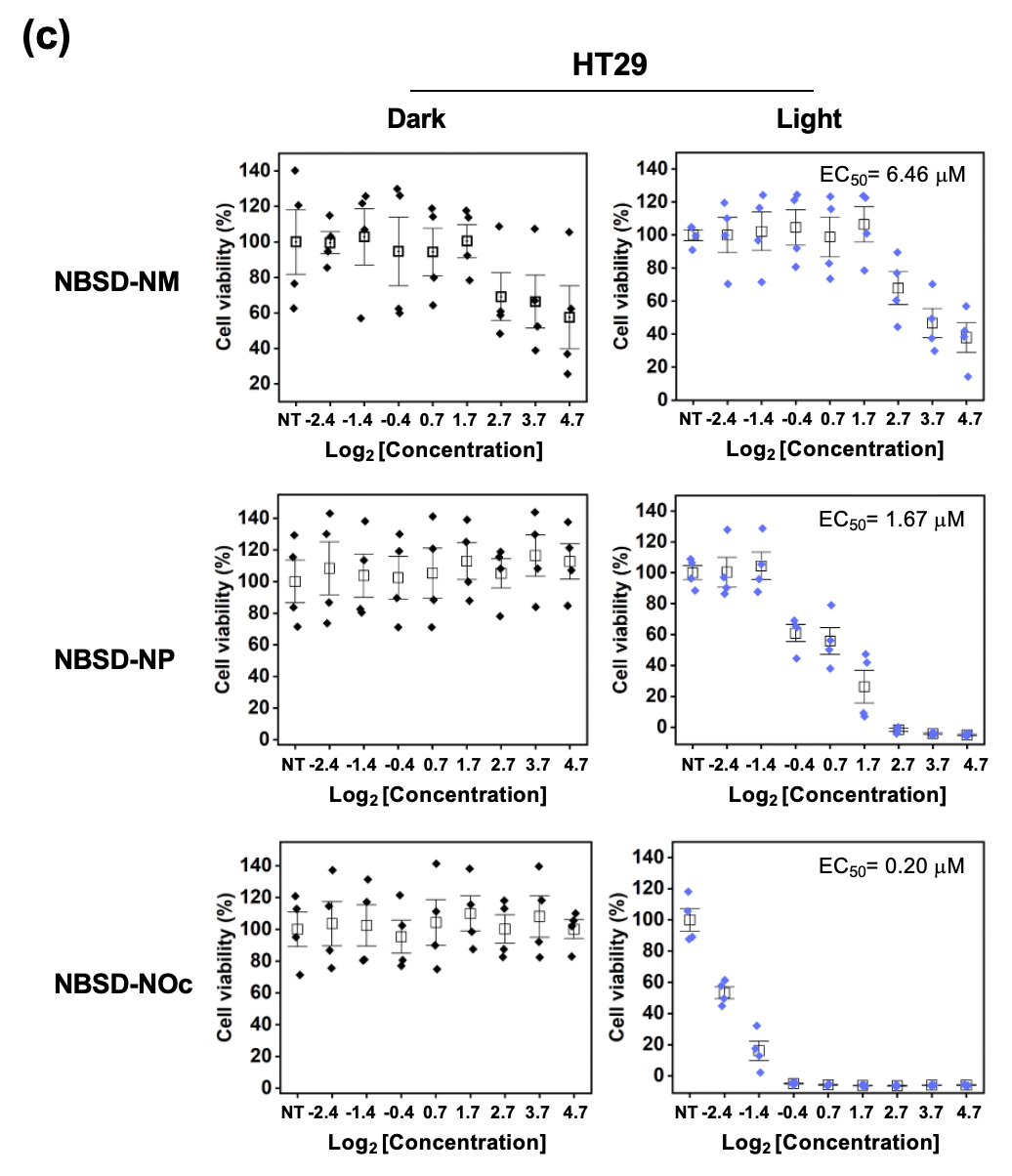


**Figure S11.** Photo-induced cytotoxicity assay of the NBSD series. Cell lines: (a) SW620, (b) LS174T, and (c) HT29. Light irradiation: 530 nm (75 mW/cm^2^, 3 min). The concentration of NBSD: 25, 12.5, 6.25, 3.12, 1.56, 0.78, 0.39, and 0.195 µM. Total treatment incubation time: 48 h (incubation time after irradiation: 24 h). Error bars represent mean ± S.D. (n= 4).


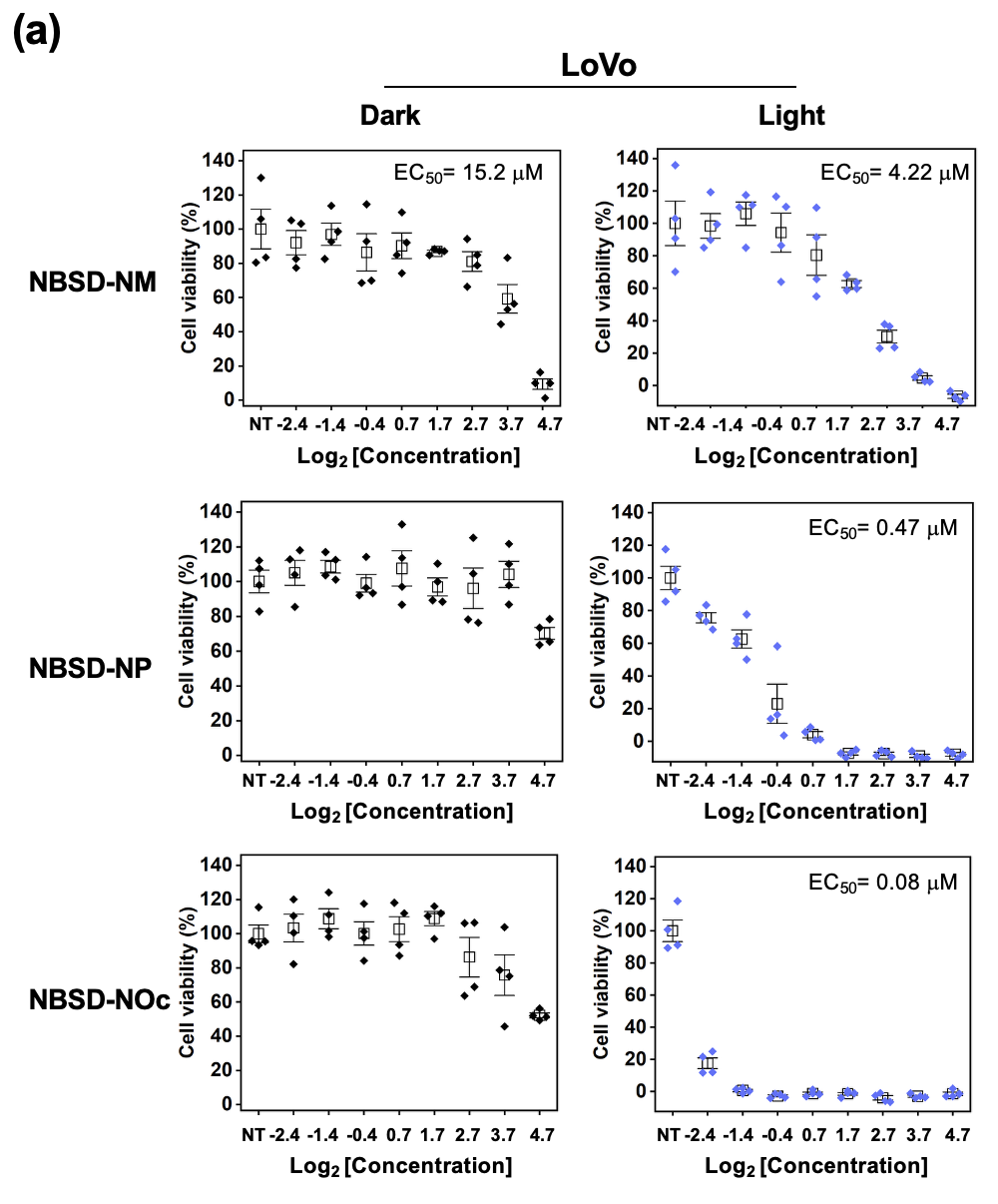

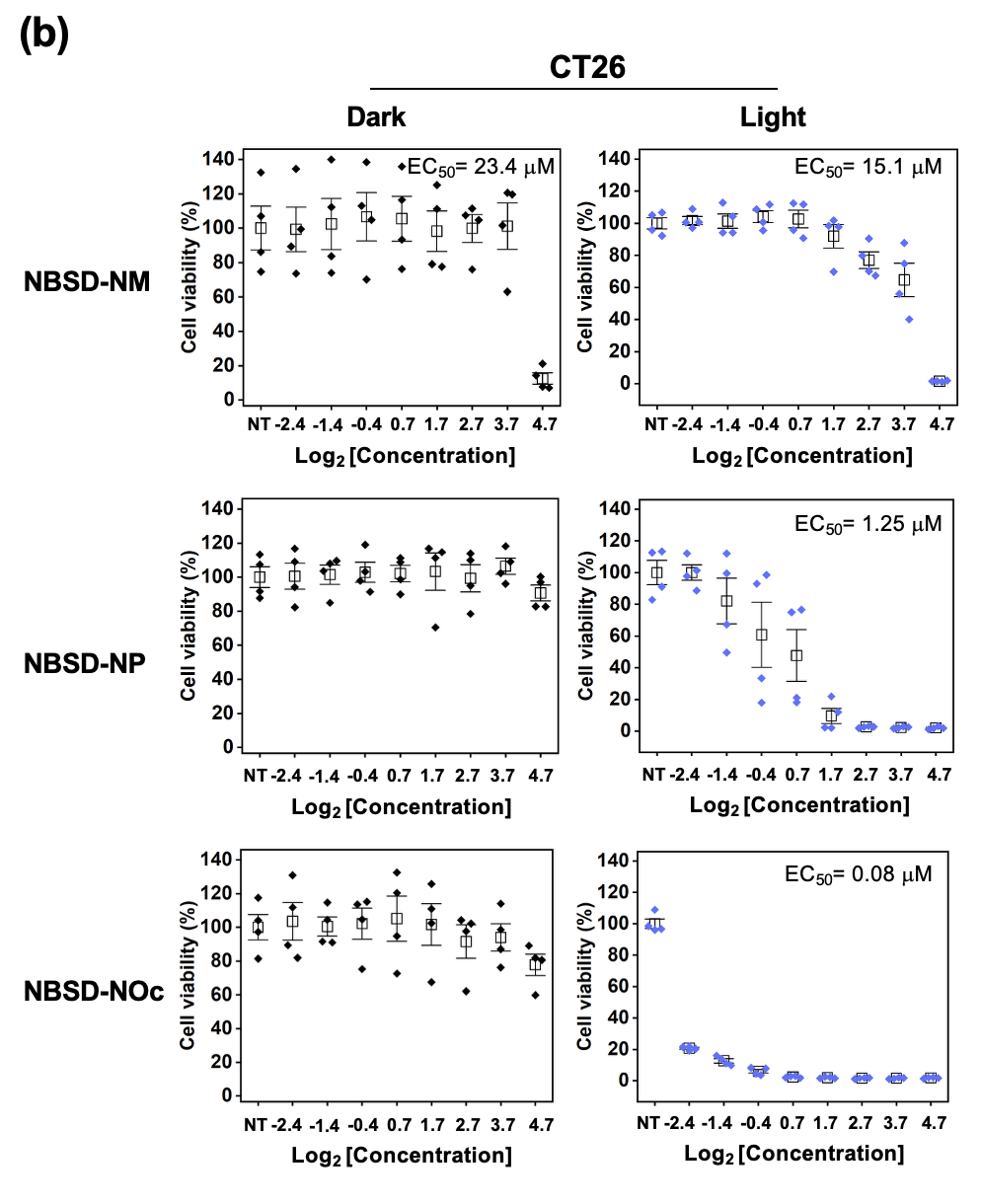


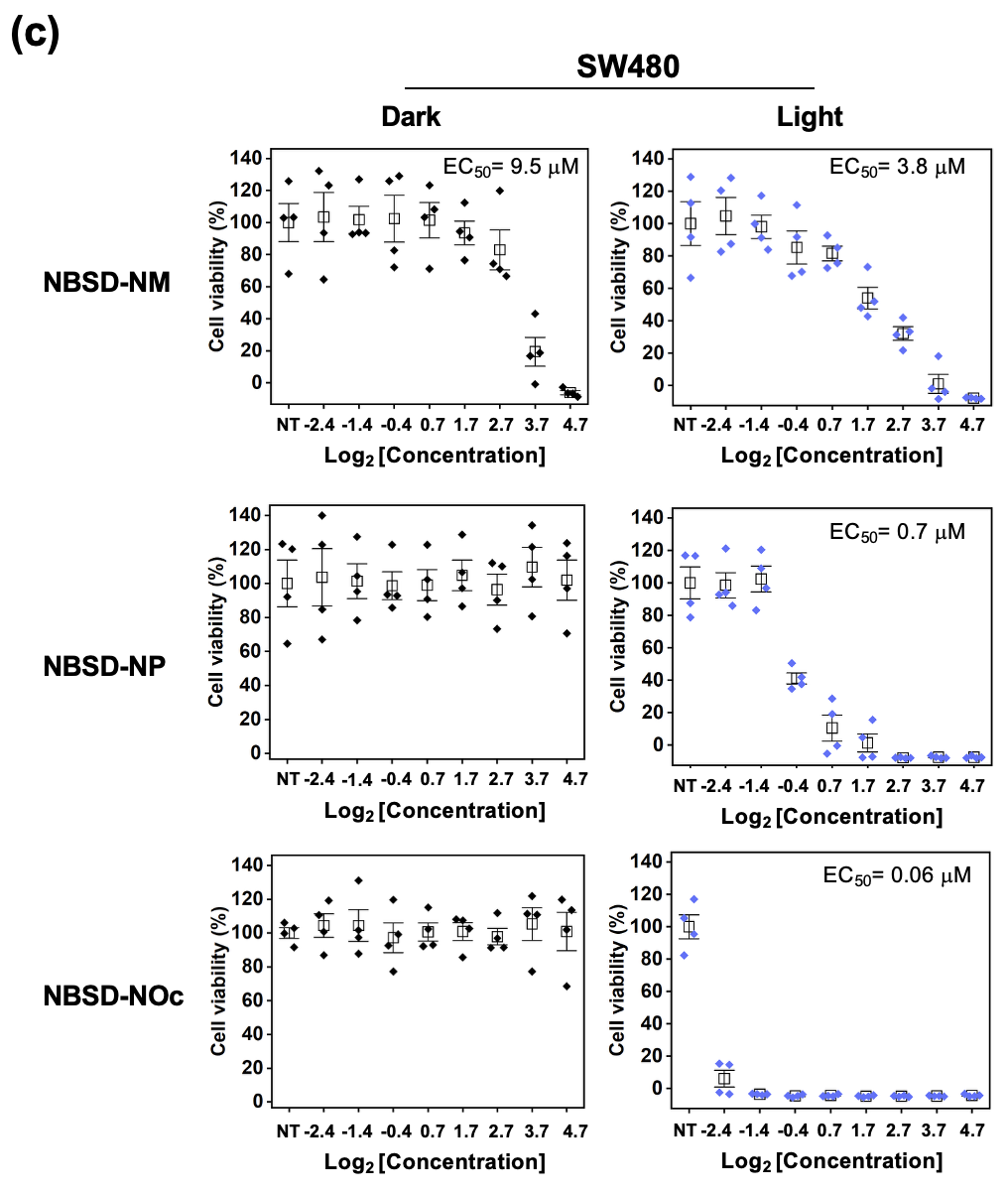


**Figure S12.** Photo-induced cytotoxicity assay of the NBSD series. Cell lines: (a) LoVo, (b) CT26, and (c) SW480. Light irradiation: 530 nm (75 mW/cm^2^, 3 min). The concentration of NBSD: 25, 12.5, 6.25, 3.12, 1.56, 0.78, 0.39, and 0.195 µM. Total incubation time: 48 h (incubation time after irradiation: 24 h). Error bars represent mean ± S.D. (n= 4).


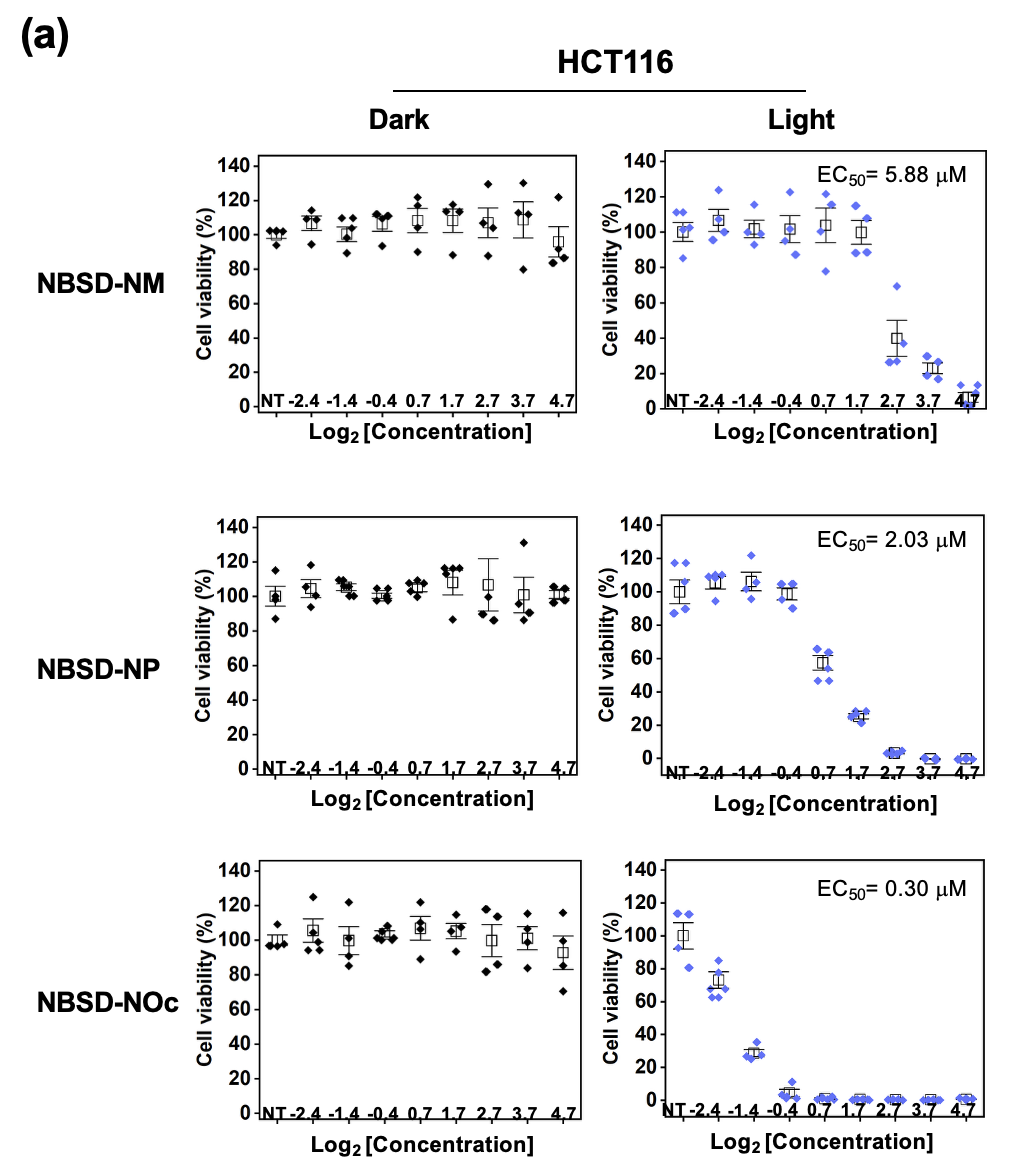

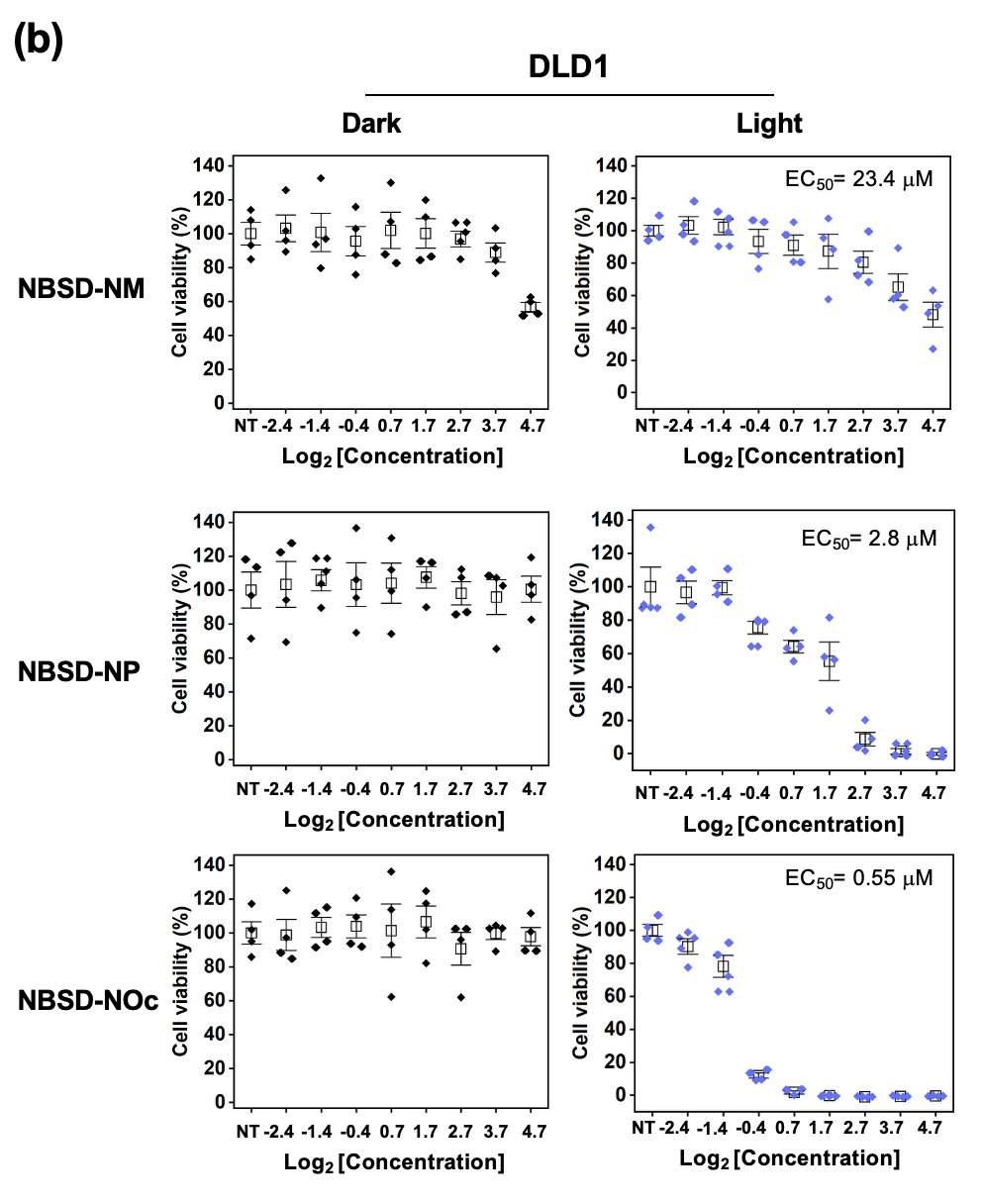


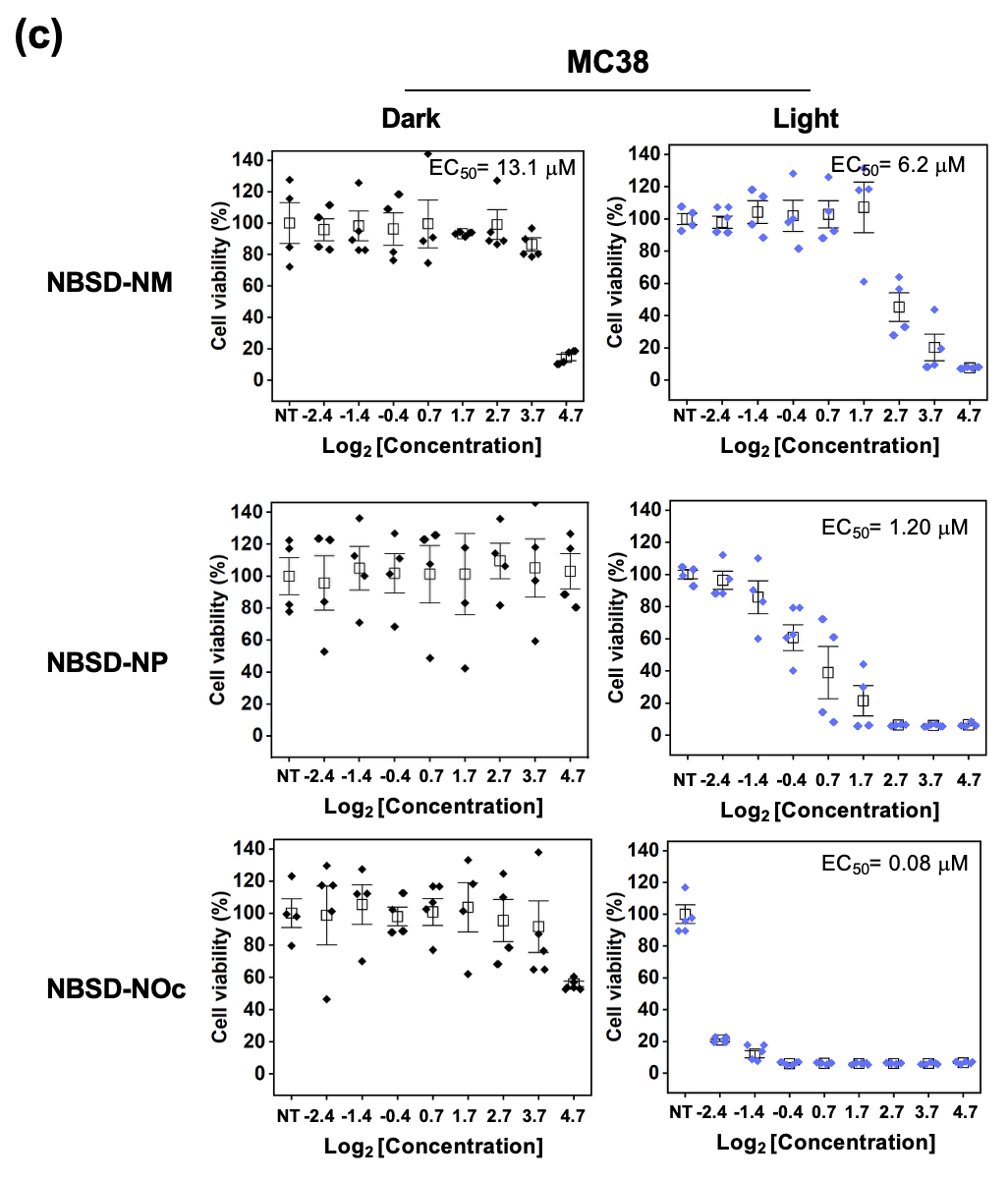


**Figure S13.** Photo-induced cytotoxicity assay of the NBSD series. Cell lines: (a) HCT116, (b) DLD1, and (c) MC38. Light irradiation: 530 nm (75 mW/cm^2^, 3 min). The concentration of NBSD: 25, 12.5, 6.25, 3.12, 1.56, 0.78, 0.39, and 0.195 µM. Total incubation time: 48 h (incubation time after irradiation: 24 h). Error bars represent mean ± S.D. (n= 4).


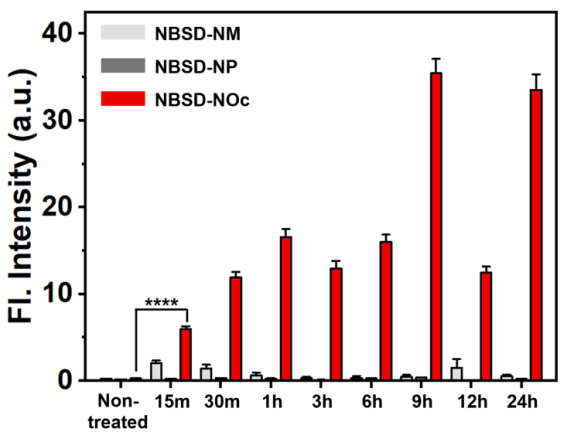


**Figure S14.** Time-course analysis (15 min to 24 h at 37 °C) for the uptake rate of the NBSD series in the CT26 cell line. Data were recorded using a confocal microscope. The concentration of NBSD was fixed at 20 µM. One-way ANOVA with Tukey’s post hoc test was used to determine statistical significance. *****P* < 0.0001


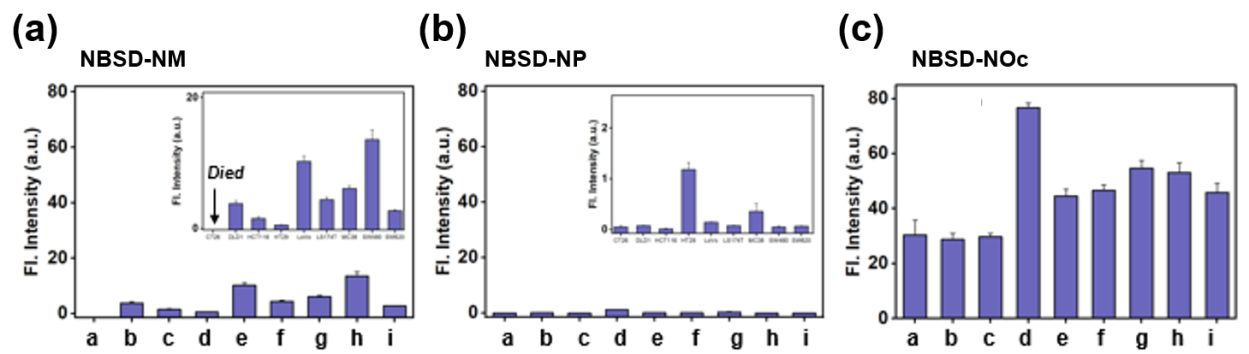


**Figure S15.** Cellular uptake rate of the NBSD series in various cell lines: (a) NBSD-NM, (b) NBSD-NP, and (c) NBSD-NOc. The concentration was fixed at 50 µM. Incubation time was 24 h at 37°C with 5% CO_2_. a: DLD1, b: CT26, c: HCT116, d: HT29, e: LoVo, f: LS174T, g: MC38, h: SW480, and i: SW620.

**
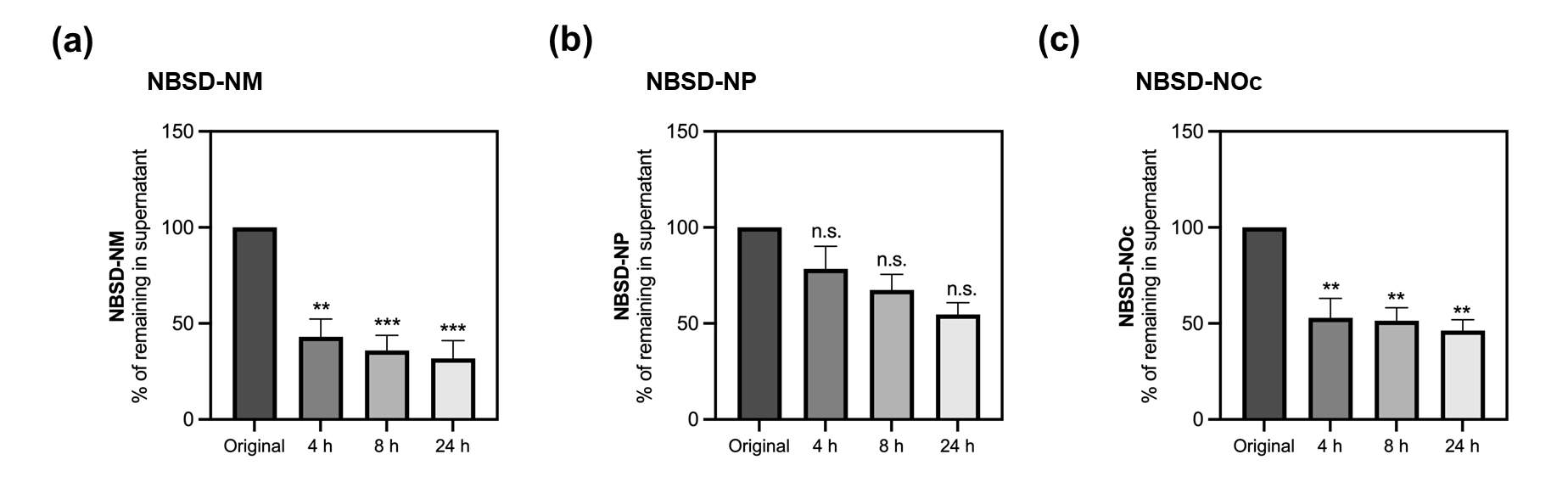
Figure S16.** Remaining NBSD series in culture medium of CT26 cells: (a) NBSD-NM, (b) NBSD-NP, and (c) NBSD-NOc. IC50 concentration was treated to CT26 cells for 4, 8, and 24 h at 37°C with 5% CO_2_. The remaining NBSD series was measured by absorbance. NBSD-NM was remaining by 43%, 35%, and 31% at 4, 8, and 24 h, respectively. NBSD-NP was remaining by 78%, 67%, and 54% at 4, 8, and 24h, respectively (n.s). NBSD-NOc was remaining by 52%, 51%, and 46% at 4, 8, and 24 h, respectively. One-way ANOVA with Tukey’s post hoc test was used to determine statistical significance. ***P* < 0.01, and ****P* < 0.001

**
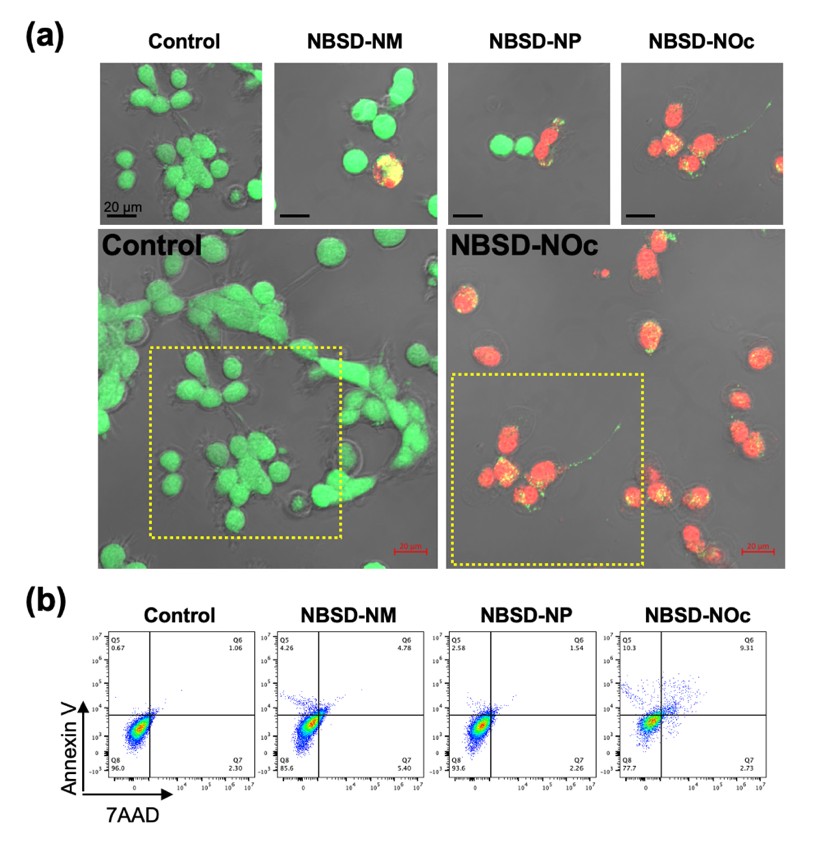
**

**Figure S17.** Live/dead cell assays in CT26 cells treated with NBSD series: (a) Live (green) and dead (red) cells were stained. Magnified view of the area indicated by the yellow box in the lower panel. (b) Flow cytometry was performed after staining cells with Annexin V and 7-AAD.


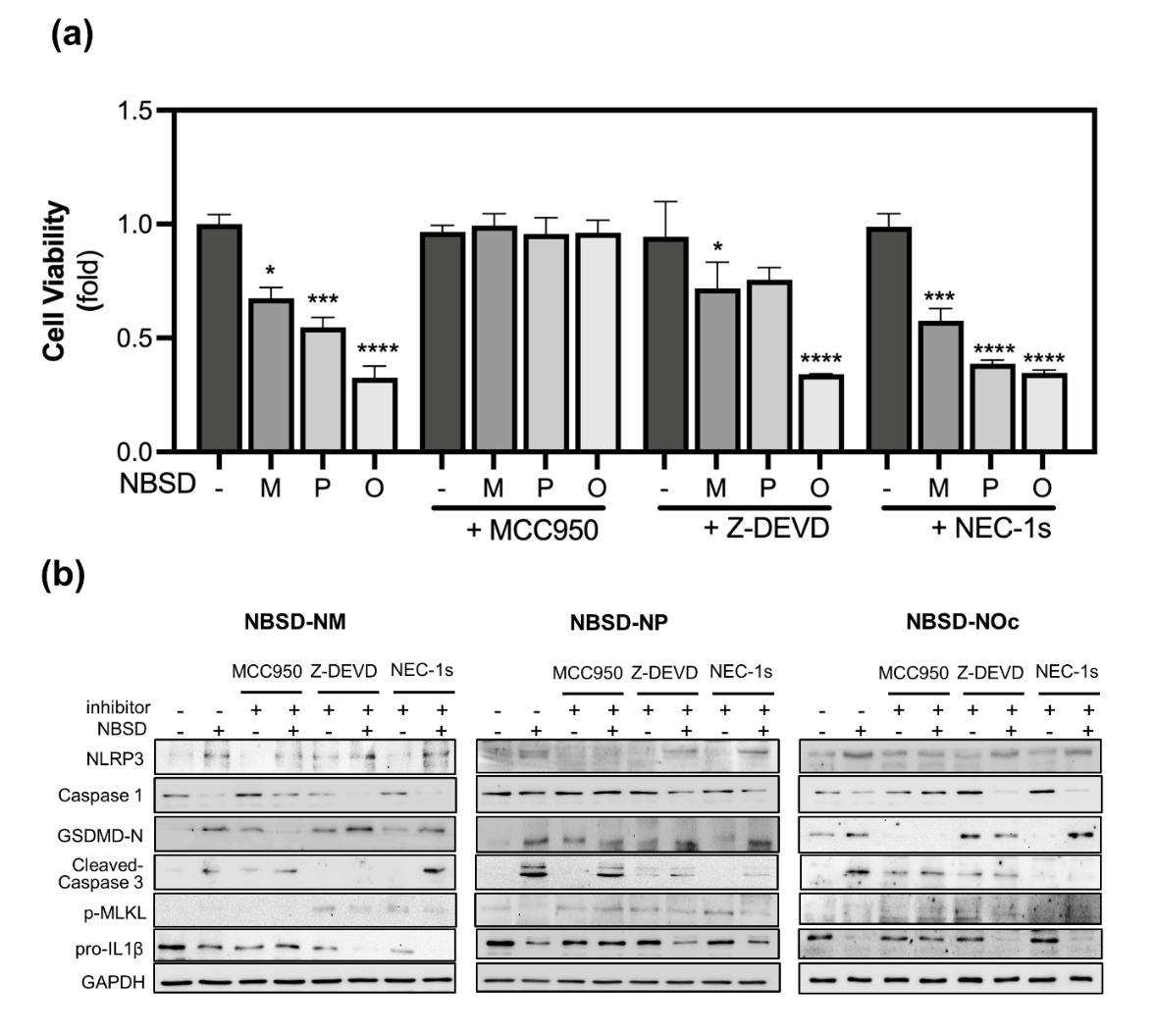


**Figure S18.** (a) CT26 cell viability by NBSD series under inhibitor pre-treatment conditions. (b) Western blot analysis for pyroptosis mechanism under inhibitor pre-treatment conditions. CT26 cells were treated with NBSD-NM, NBSD-NP, or NBSD-NOc at their respective IC_50_ concentrations. MCC950 (NLRP3 inhibitor) was used for pyroptosis inhibition, Z-VED-MFK (caspase 3/7 inhibitor) was used for apoptosis inhibition, and NEC-1s (MLKL inhibitor) was used for necroptosis inhibition. One-way ANOVA with Tukey’s post hoc test was used to determine statistical significance. **P* < 0.05, ****P* < 0.001, and *****P* < 0.0001


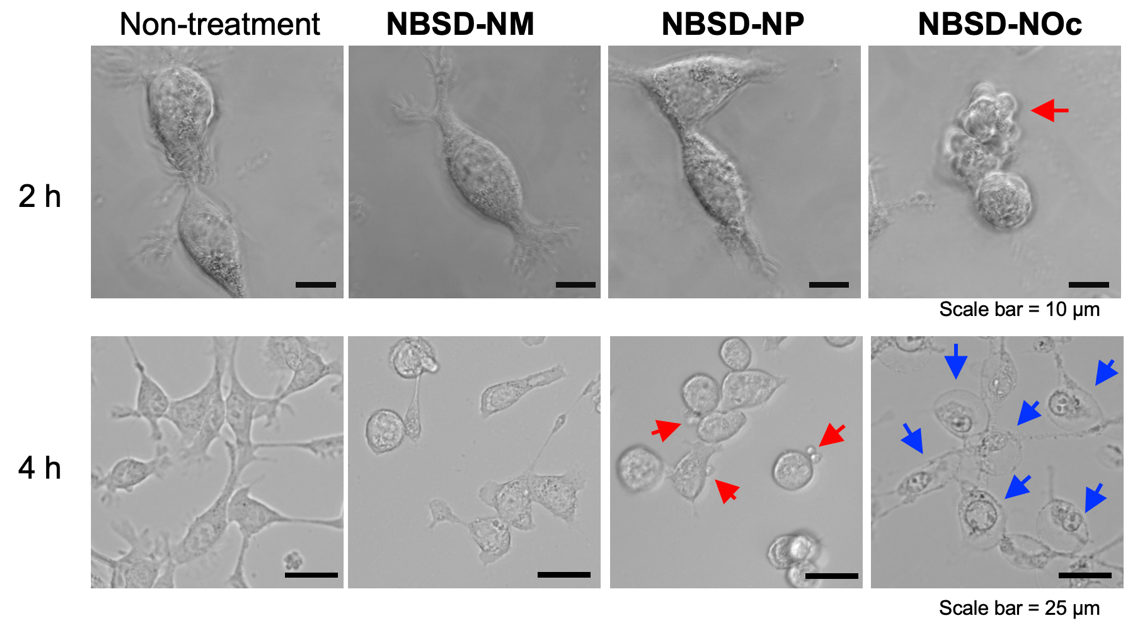


**Figure S19.** Time-lapse DIC images showing cell elongation and protrusion formation. Red arrowheads indicate budding, and blue arrowheads indicate membrane rupture.


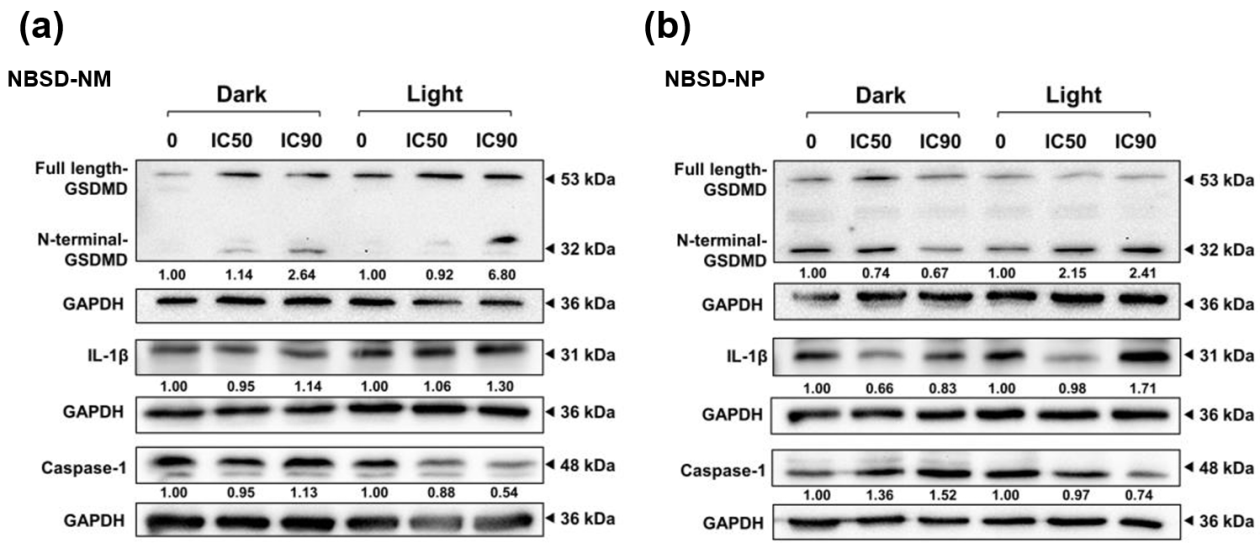


**Figure S20.** Western blot analysis for the pyroptosis mechanism. Loading protein: 30 µg. CT26 cells were treated with NBSD-NM, NBSD-NP, or NBSD-NOc at their respective IC50 and IC90 concentrations. Data for NBSD-NOc are presented in Figure 4f.


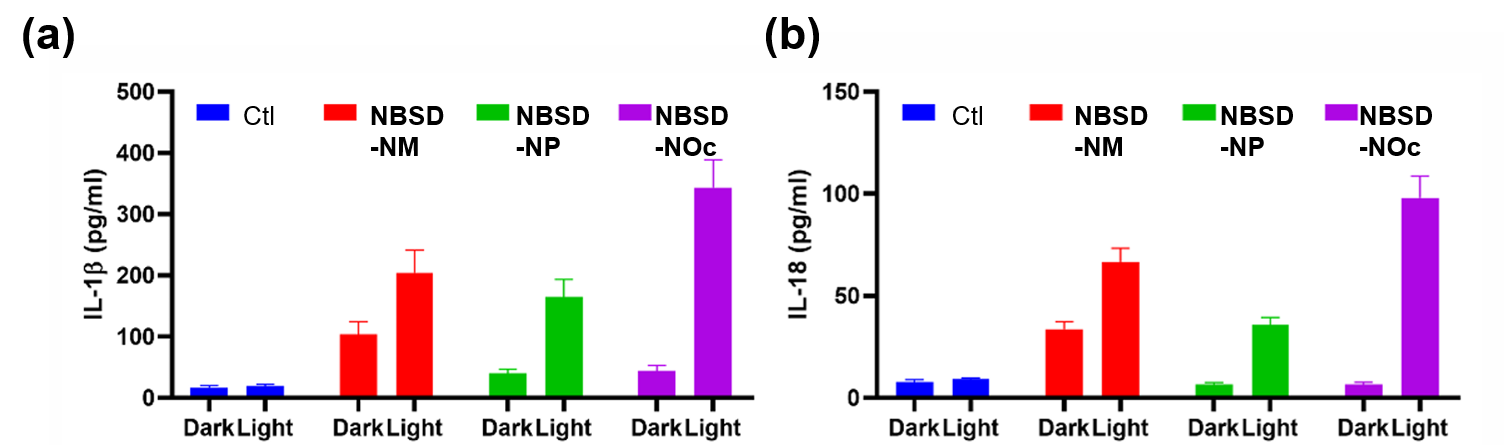


**Figure S21.** (a) IL-1β and (b) IL-18 levels (pg/mL) were measured as key markers of pyroptotic cell death under different experimental conditions. CT26 cells were treated with NBSD-NM, NBSD-NP, or NBSD-NOc at their respective IC50 concentrations. Data are presented as mean ± standard error (SE).


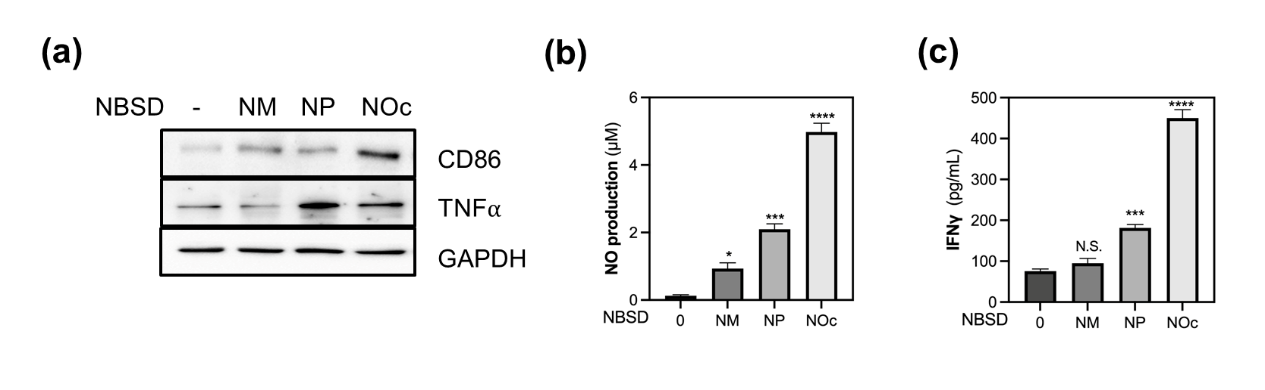


**Figure S22.** (a) Western blot of M1 markers, (b) NO production, and (c) IFNγ level in Raw264.7 murine macrophages incubated in conditioned medium (CM) from NBSD-NOc-treated CT26 colon cancer cells. One-way ANOVA with Tukey’s post hoc test was used to determine statistical significance. **P* < 0.05, ****P* < 0.001, and *****P* < 0.0001


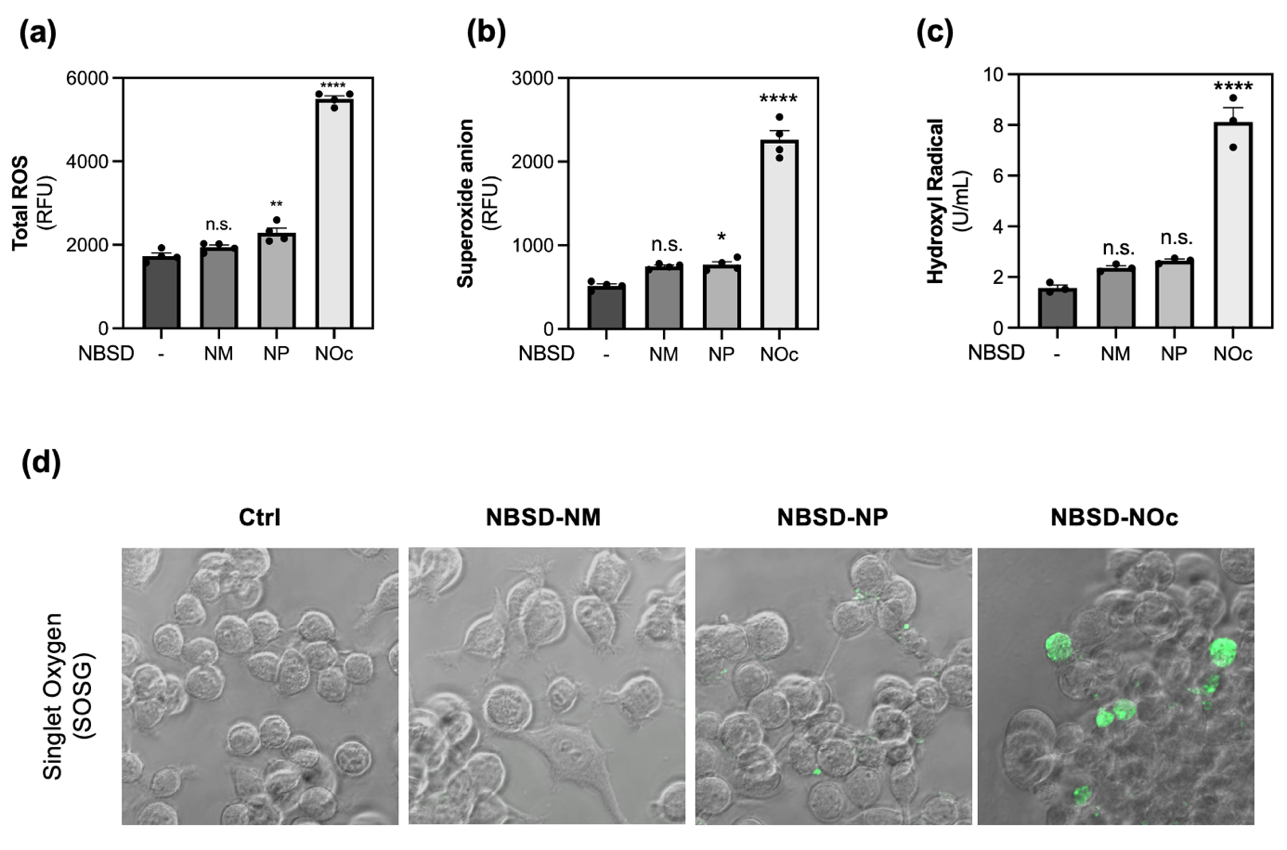


**Figure S23.** (a) Total ROS, (b) superoxide anion, (c) hydroxyl radical, and (d) singlet oxygen levels were measured in CT26 cells treated with NBSD-NM, NBSD-NP, or NBSD-NOc at their respective IC50 concentrations. Data are presented as mean ± standard error (SE). One-way ANOVA with Tukey’s post hoc test was used to determine statistical significance. **P* < 0.05, ***P* < 0.01, and *****P* < 0.0001


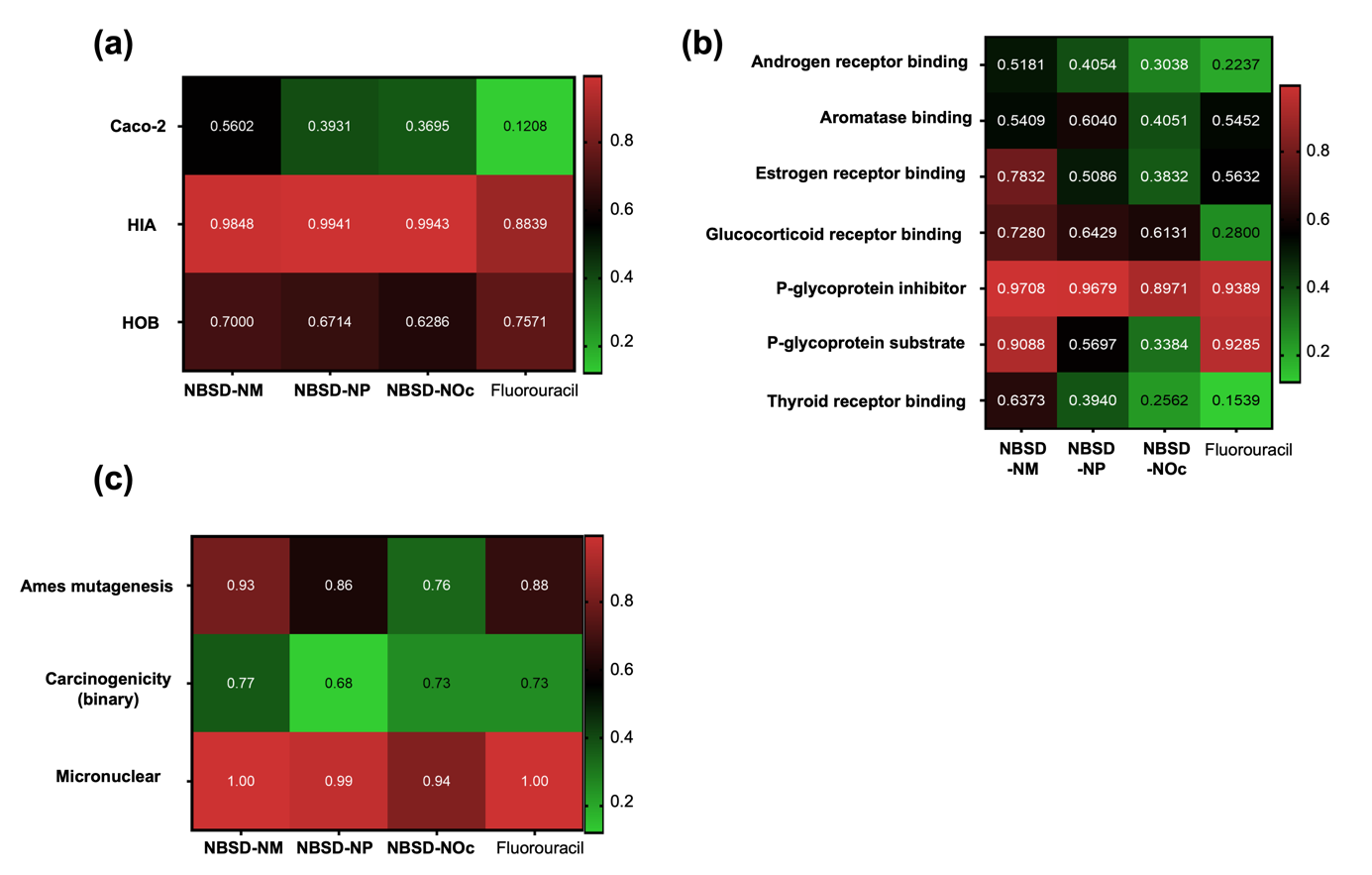


**Figure S24.** Computational pharmacokinetic profiles for (a) administration, (b) binding potential to hormone-related proteins, and (c) genetic toxicity. Fluorouracil, an FDA-approved drug for human colorectal cancer, was used as a reference. The analysis was obtained using SwissADME prediction (accuracy: 72–94%,(2)).


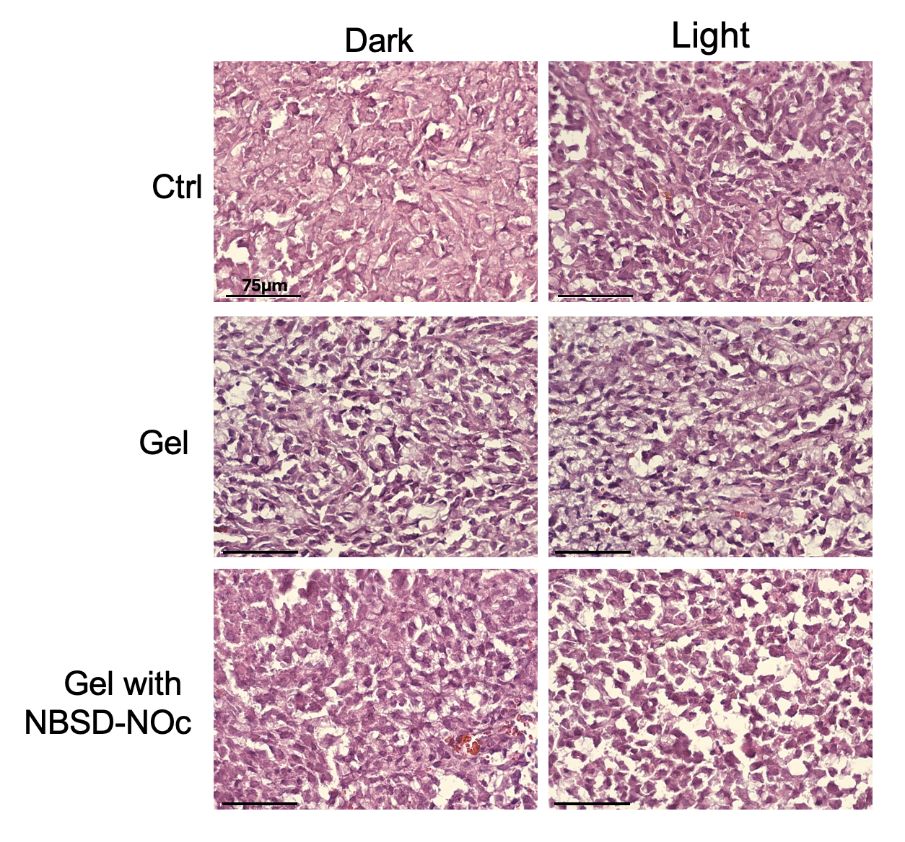


**Figure S25.** Representative H&E staining results of the CT26 tumor in each group. Scale bar: 75 μm.


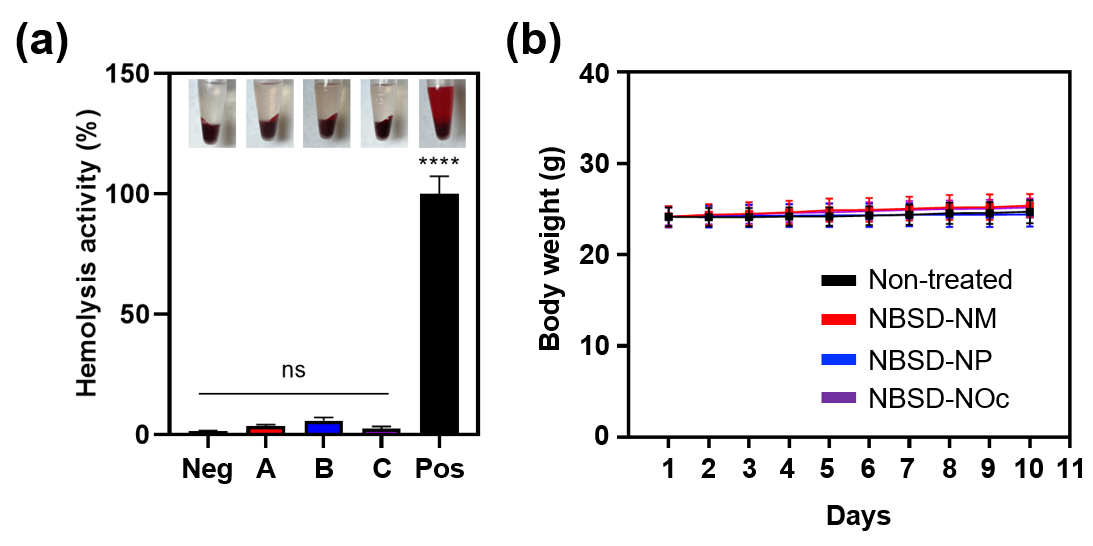


**Figure S26.** (a) Hemolysis assay of the NBSD series: A, NBSD-NM; B, NBSD-NP; and C, NBSD-NOc. The concentration of the NBSD series was 0.272 µg/mL. Neg: negative control. Pos: positive control, triton-X100. (b) Body weight change of C57BL/6 mice after NBSD series administration (mpk: 272 µg/kg, i.v.) for 10 consecutive days.


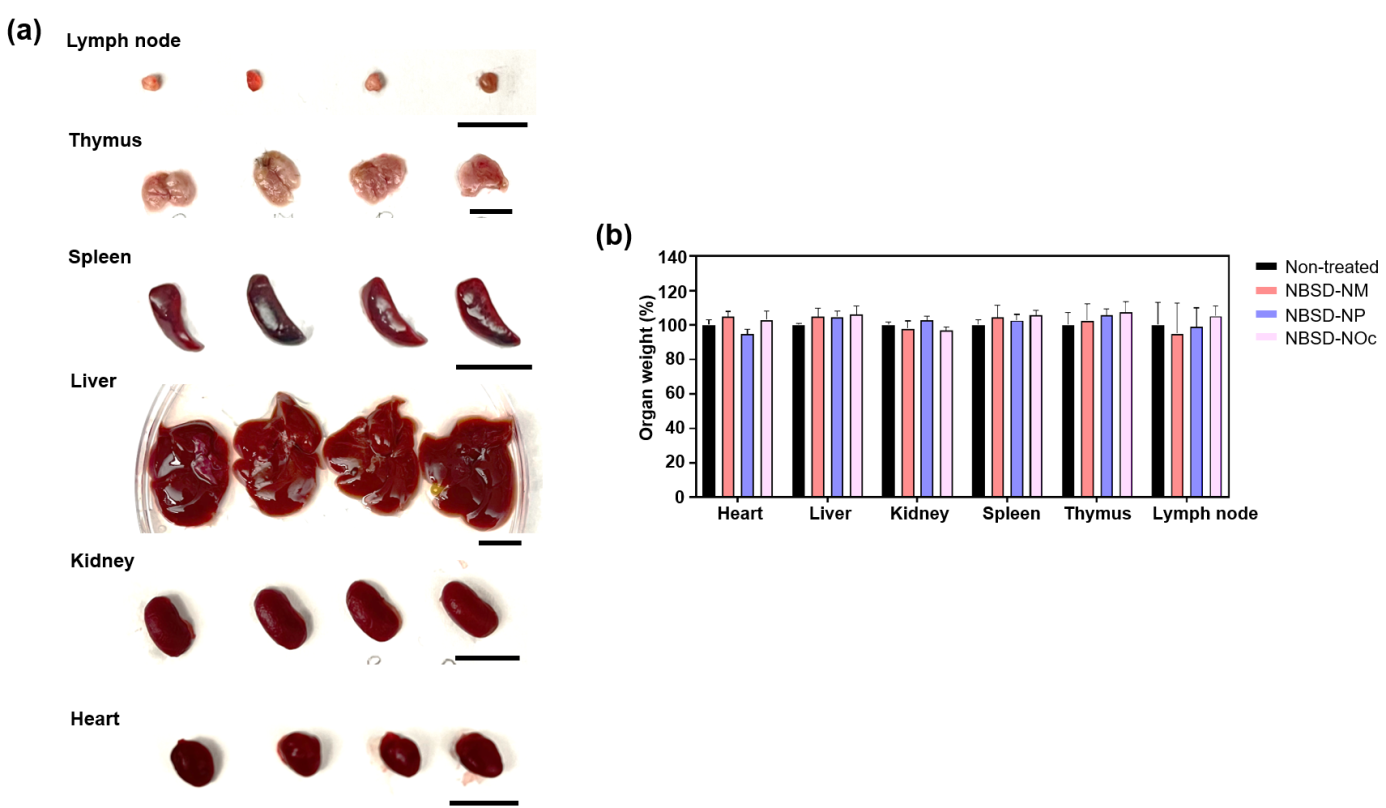


**Figure S27.** (a) Representative images and (b) weights (% to control) of lymph node, thymus, spleen, liver, kidney, and heart from C57BL6 mice treated with the NBSD series (mpk: 0.272 mg/kg, i.v.) for 10 consecutive days. Data are presented as mean ± SE.

**^
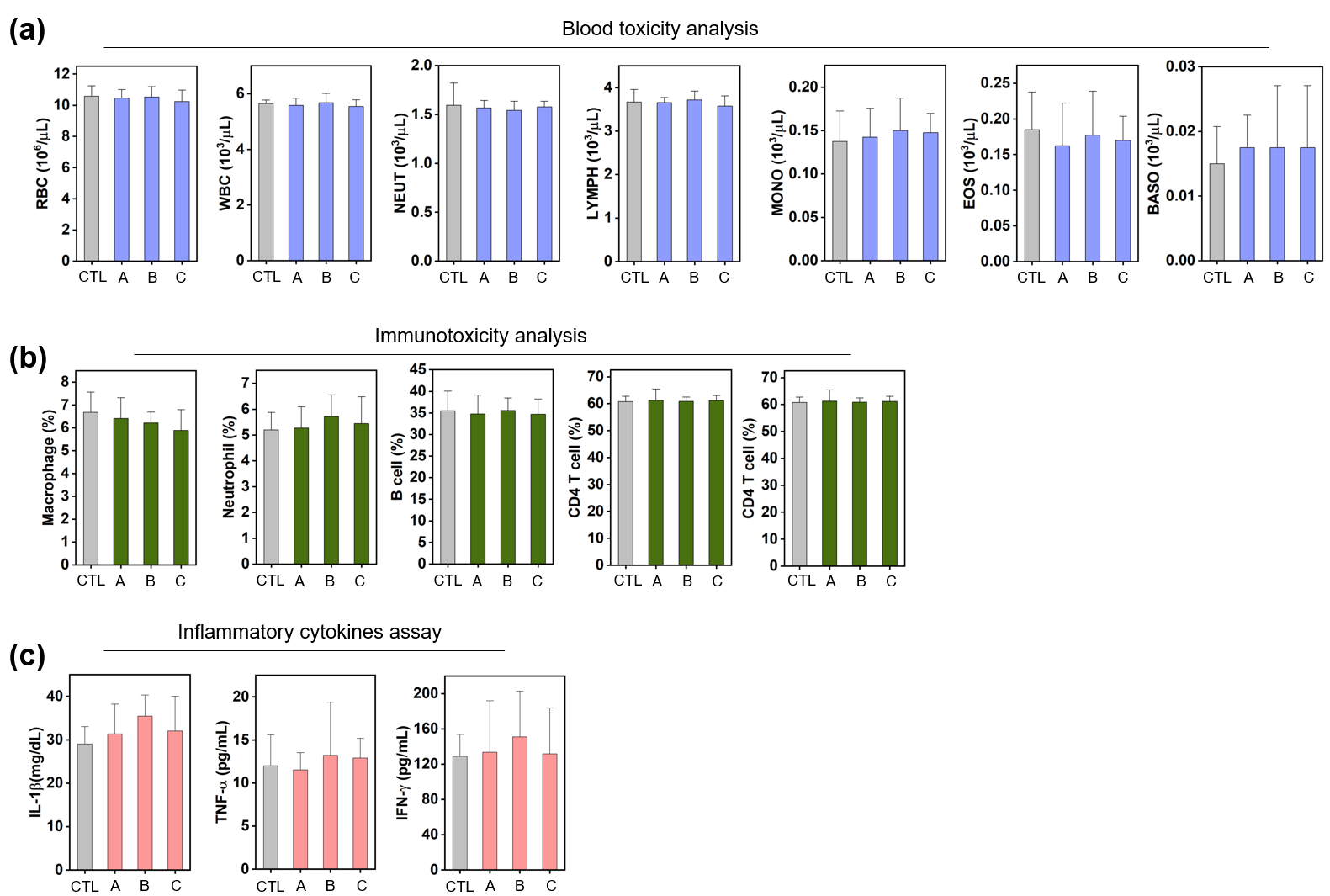
^**

**Figure S28.** (a) Blood cell counts, including red blood cells, white blood cells, neutrophils, lymphocytes, monocytes, eosinophils, and basophils, were measured using a blood analyzer to assess blood toxicity. (b) Percentages of immune cell populations, including macrophages, neutrophils, B cells, CD4^+^ T cells, and CD8^+^ T cells, were analyzed using flow cytometry to assess immunotoxicity. (c) The levels of IL-1β, TNF-α, and IFN-γ were measured to evaluate systemic inflammatory responses following NBSD series administration. C557BL6 mice were administered the NBSD series (mpk: 0.272 mg/kg, i.v.) for 10 consecutive days prior to measurements. Data are presented as mean ± SE. CTL: control. A: NBSD-NM. B: NBSD-NP. C: NBSD-NOc.

**^
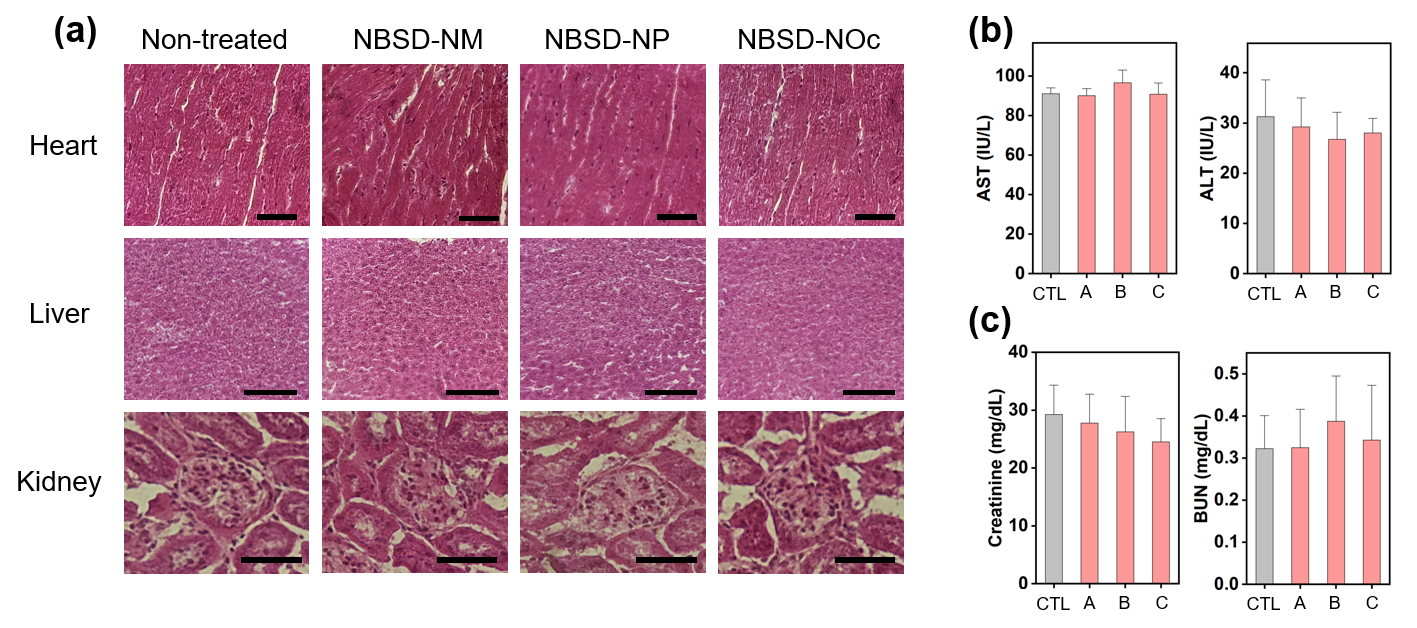
^**

**Figure S29.** (a) Representative H&E-stained tissue sections of the heart, liver, and kidney were observed to assess organ toxicity. (b) Serum levels of aspartate aminotransferase (AST) and alanine aminotransferase (ALT) were measured to assess hepatic toxicity. (c) Serum levels of creatinine and blood urea nitrogen (BUN) were measured to evaluate renal toxicity following NBSD series administration. C57BL6 mice were administered the NBSD series (mpk: 0.272 mg/kg, i.v.) for 10 consecutive days prior to measurements. Data are presented as mean ± SE. CTL: control. A: NBSD-NM. B: NBSD-NP. C: NBSD-NOc.

**[^1^H/^13^C-NMR, Mass Data]**

^1^H-NMR of 4-fluoro-benzoselenadiazole


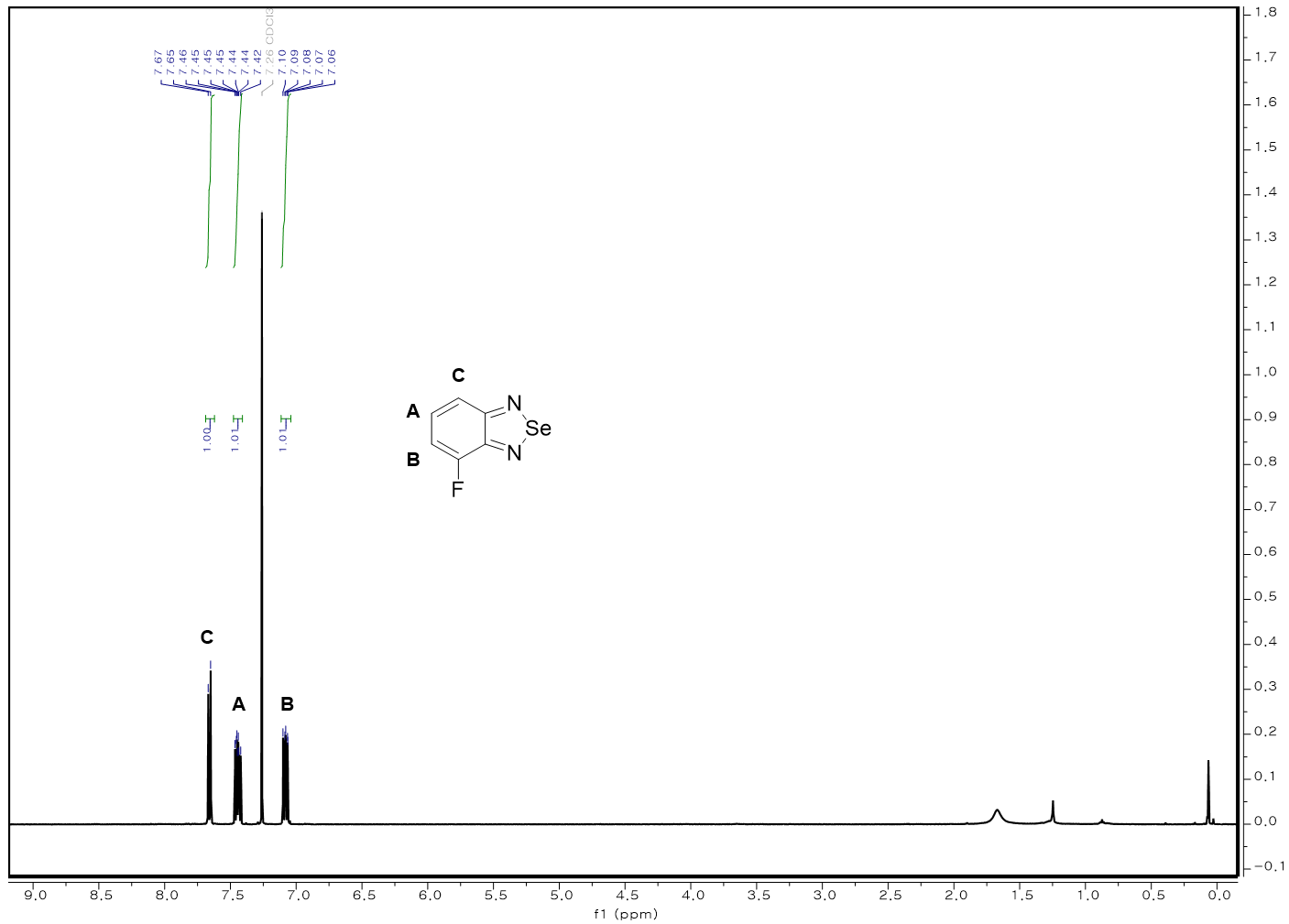


^1^H-NMR of 4-fluoro-nitrobenzoselenadiazole


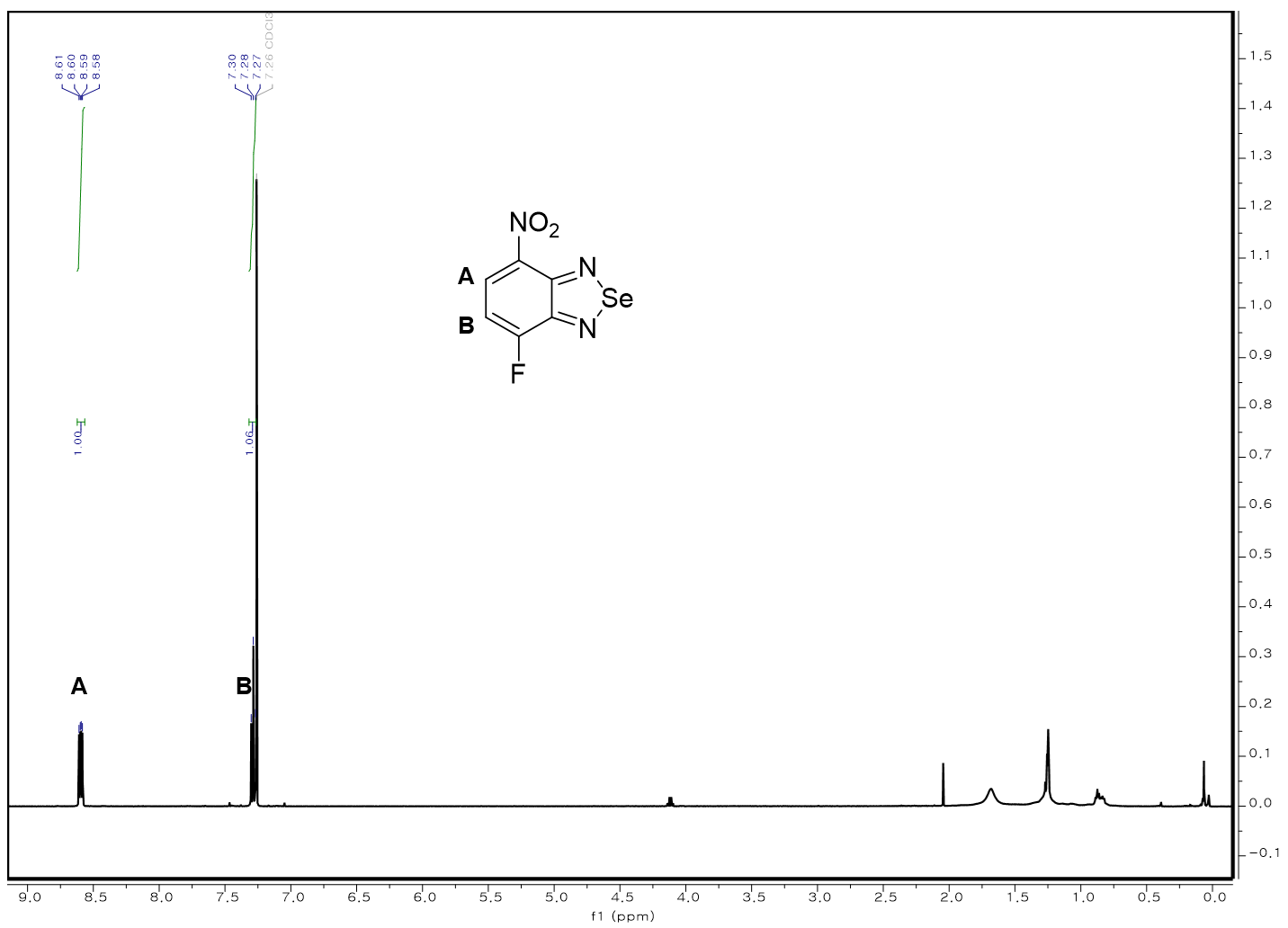


^1^H-NMR of **NBSD-NM**


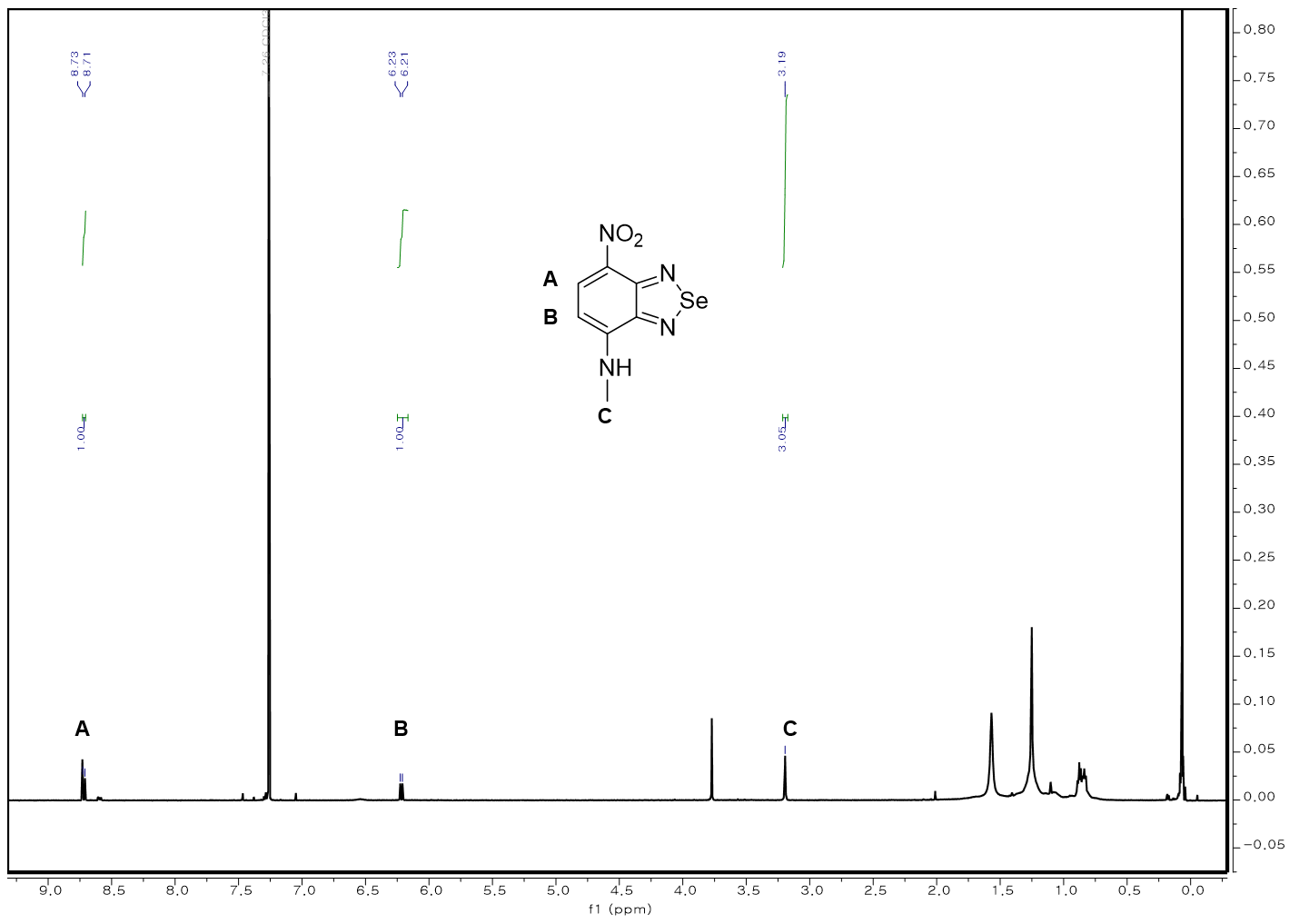


^13^C-NMR of **NBSD-NM**


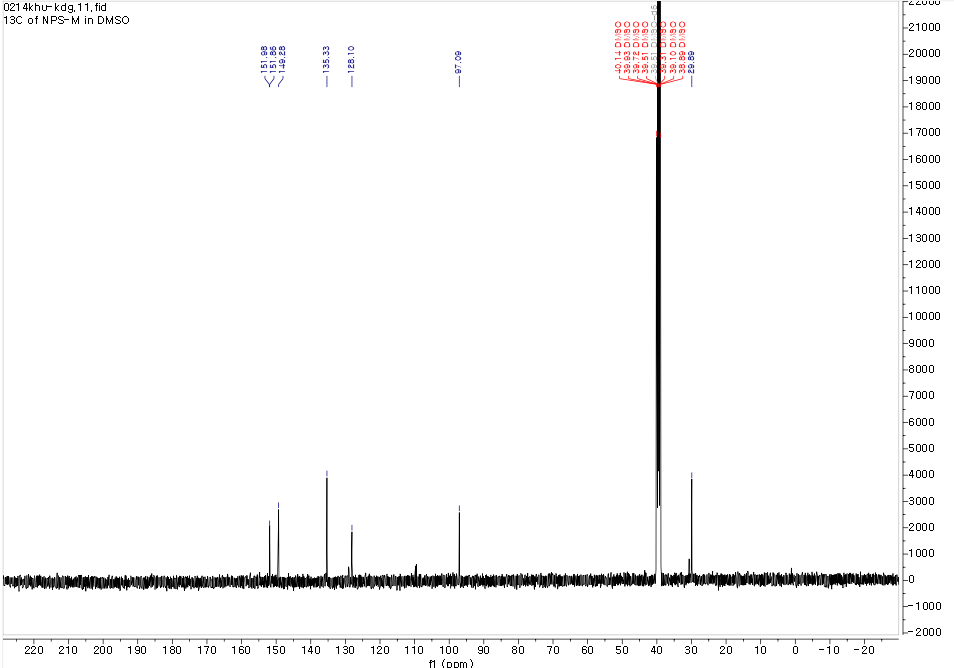


Mass of **NBSD-NM**


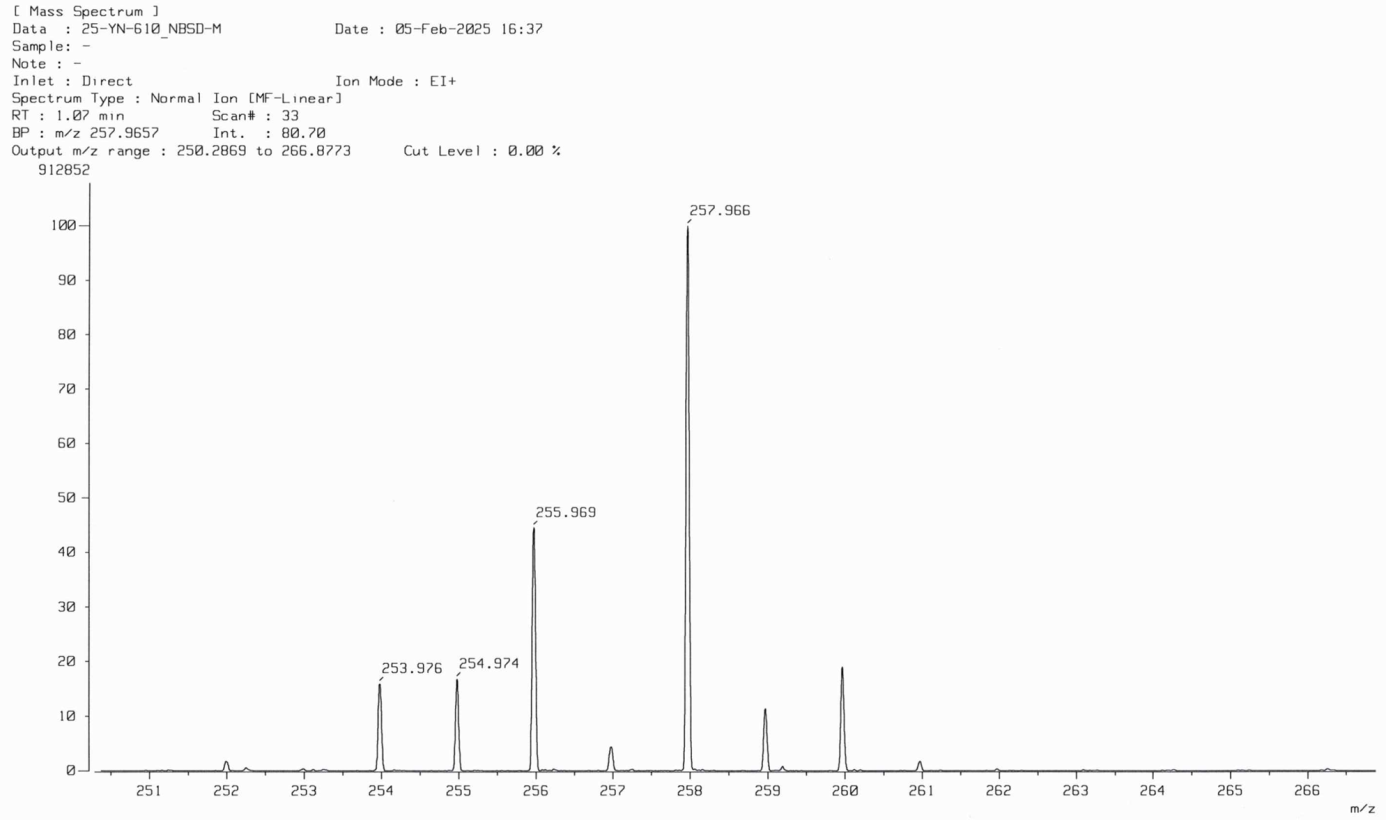


^1^H-NMR of **NBSD-NP**


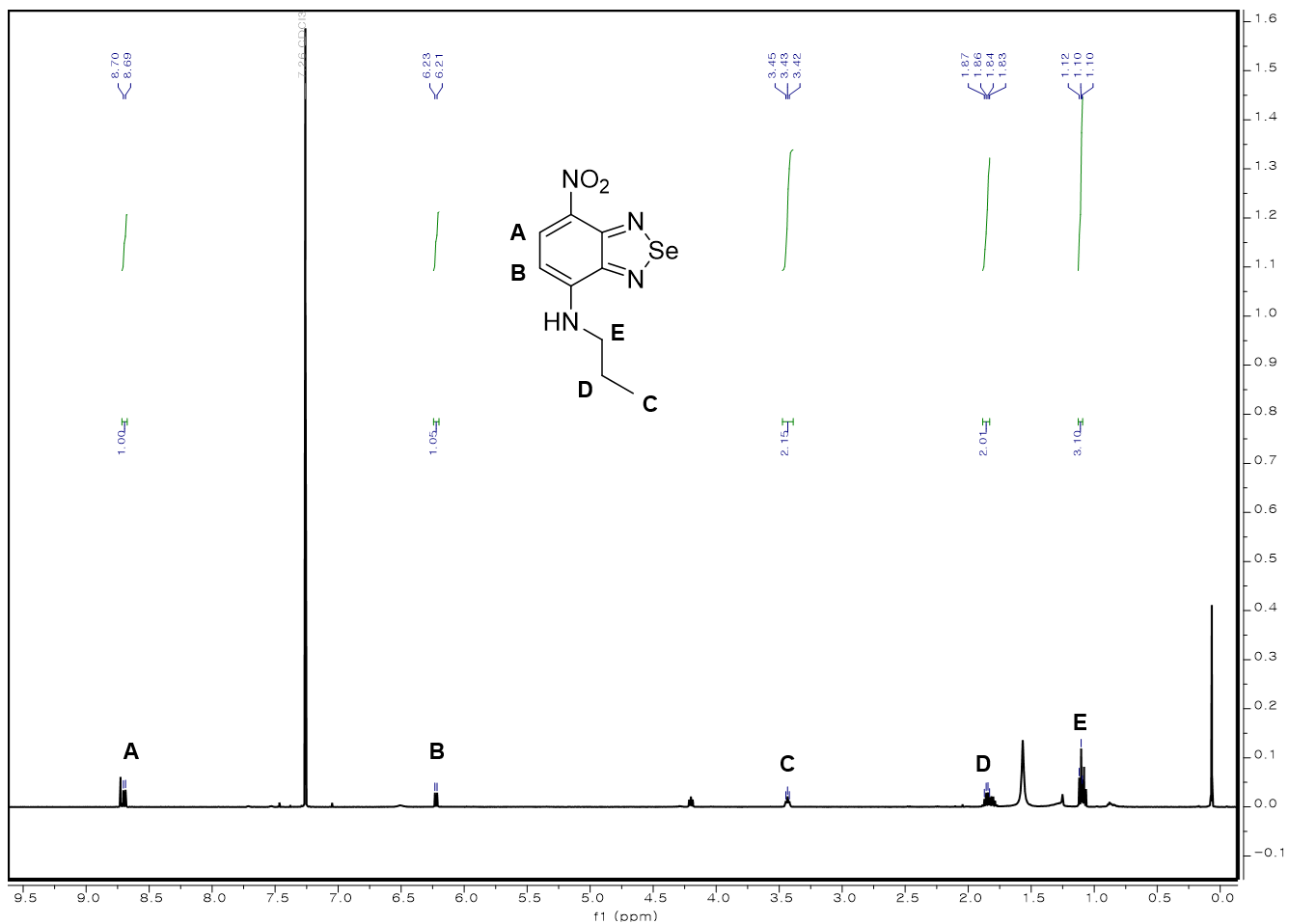


^13^C-NMR of **NBSD-NP**


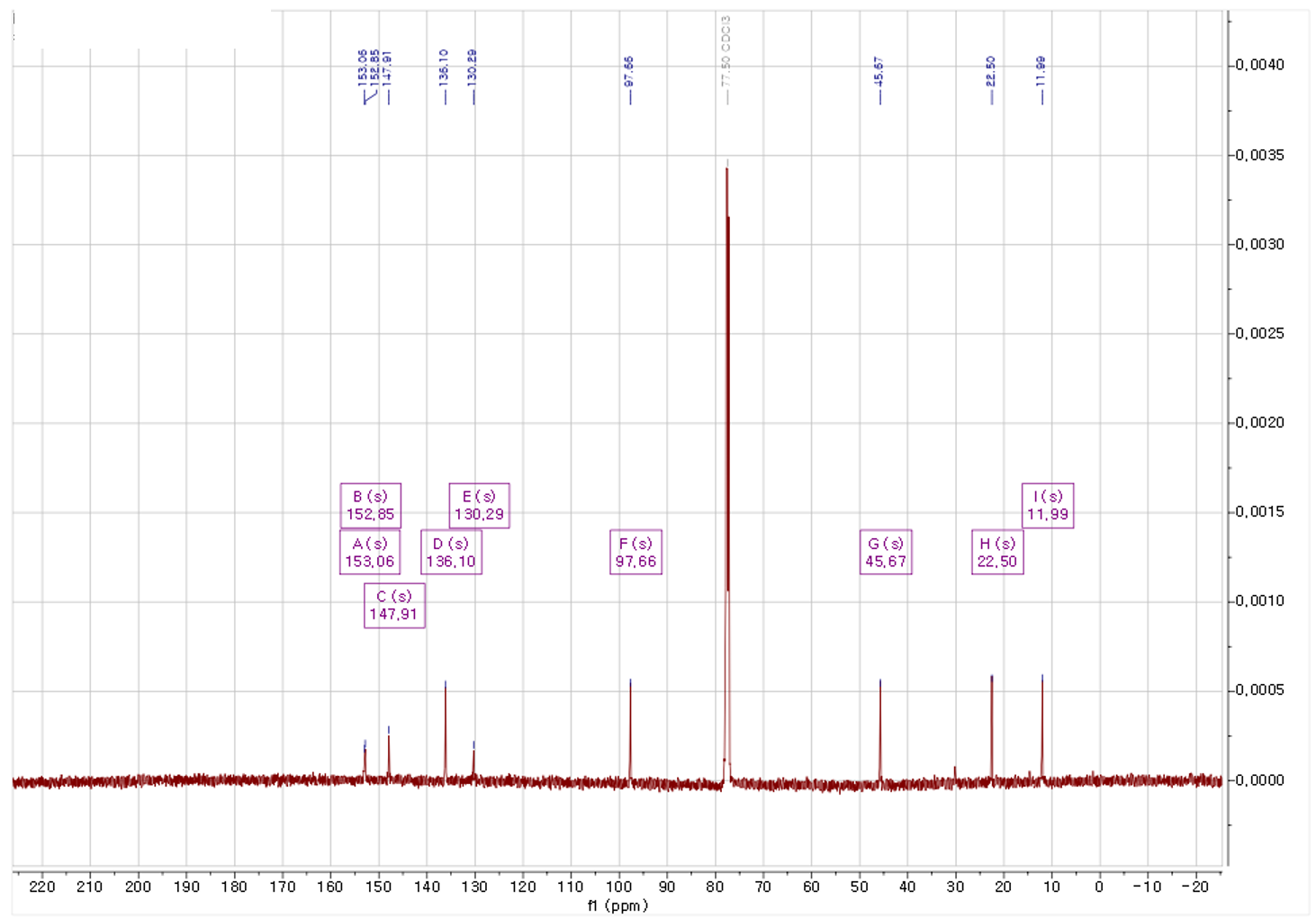


Mass of **NBSD-NP**


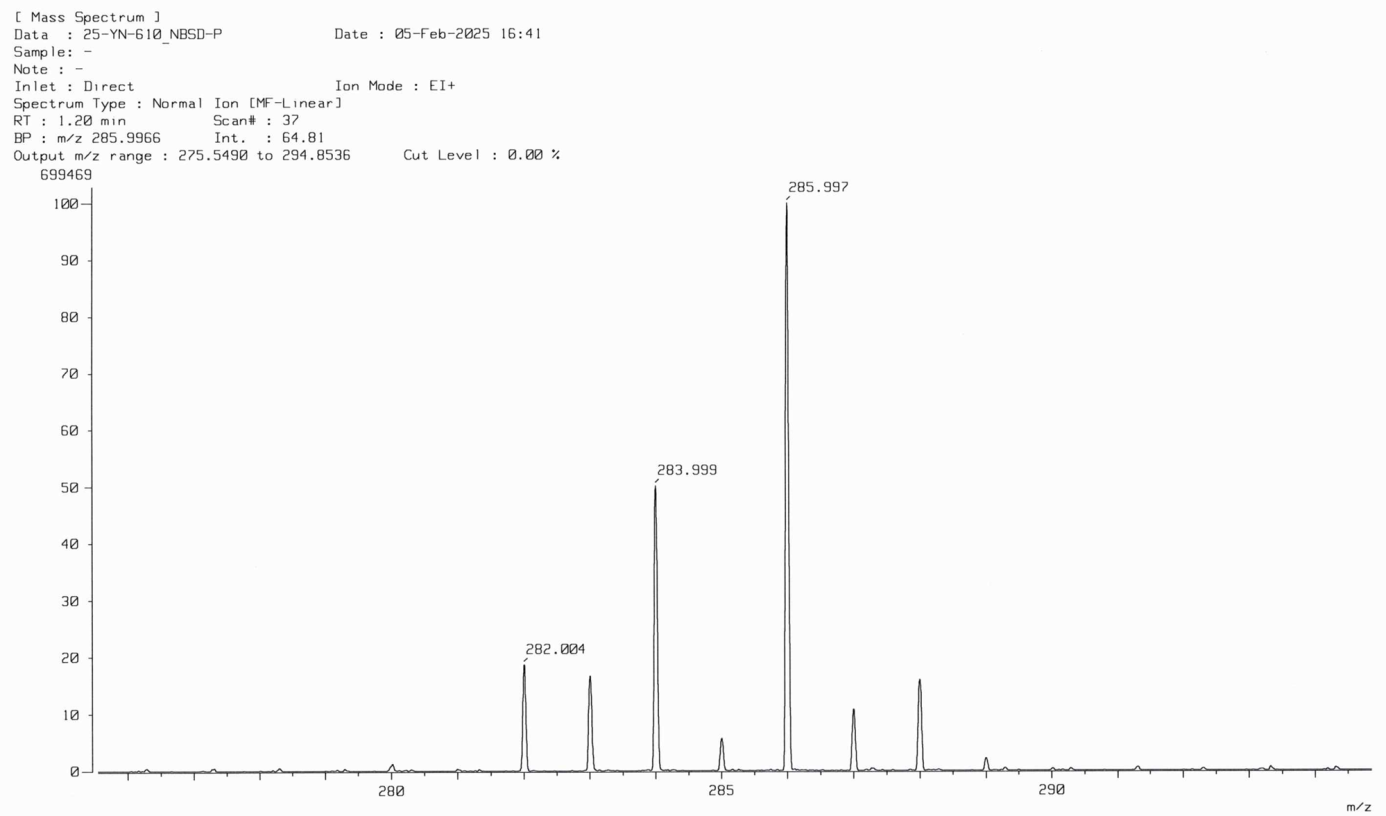


^1^H-NMR of **NBSD-NOc**


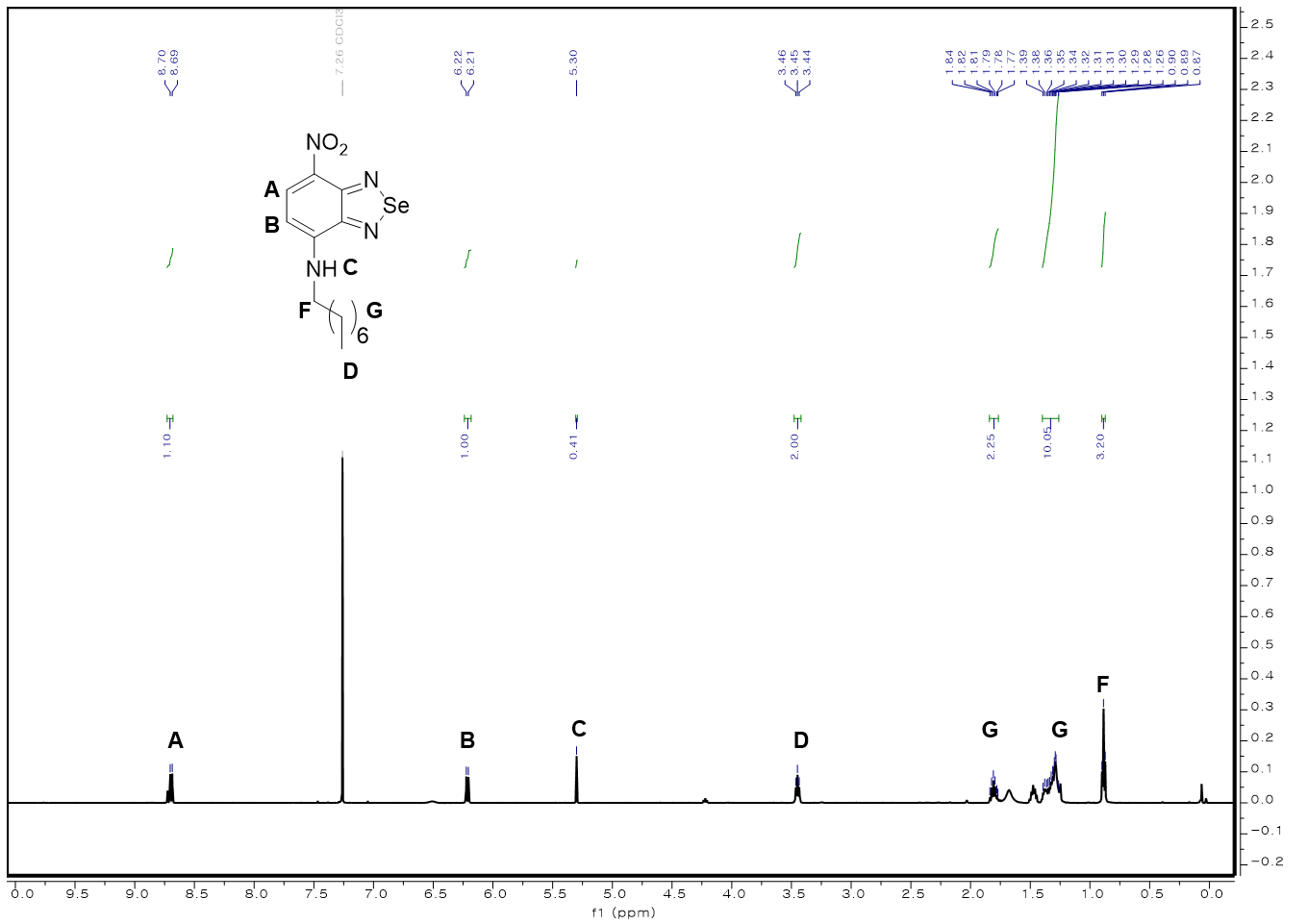


^13^C-NMR of **NBSD-NOc**


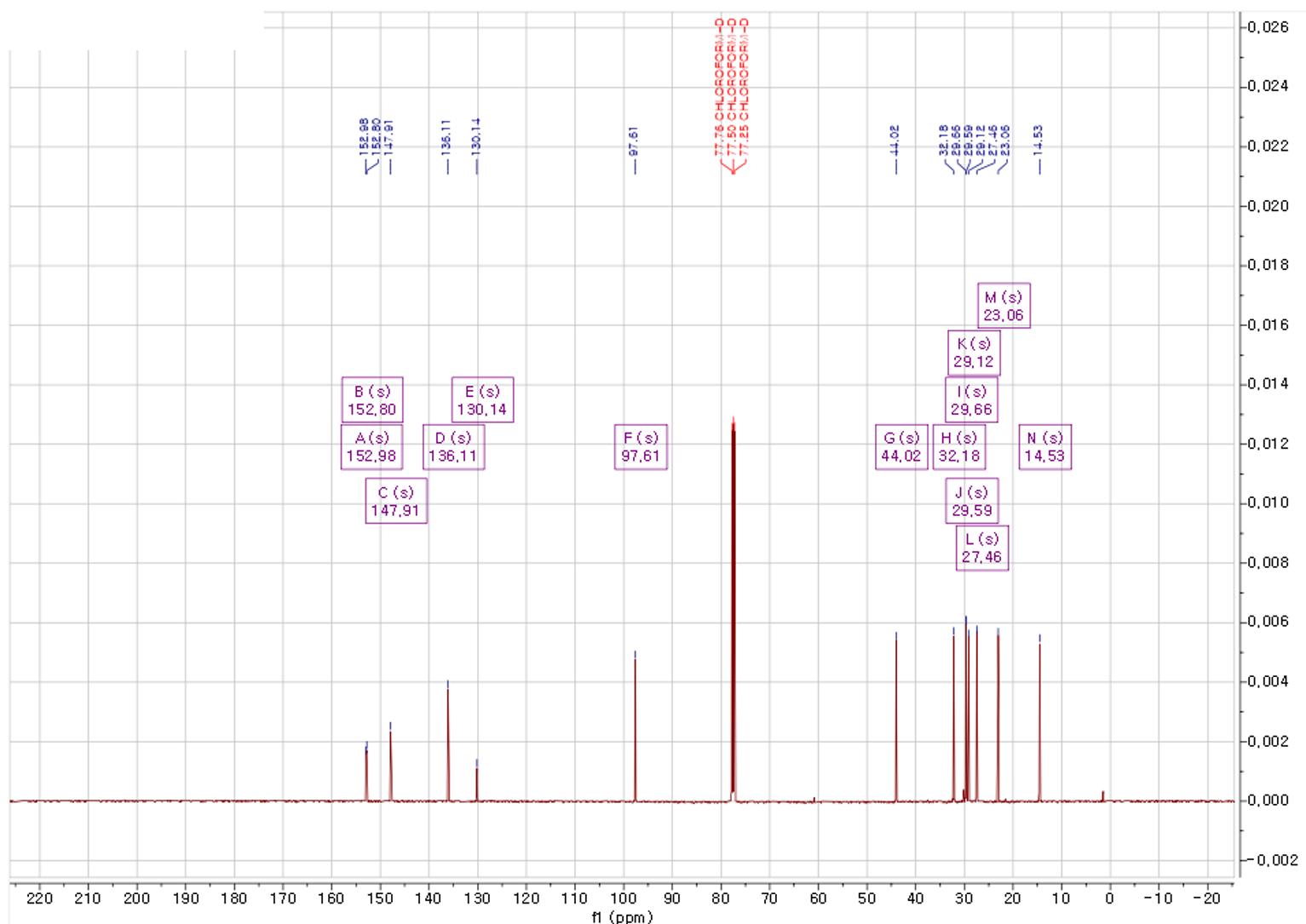


Mass of **NBSD- NOc**


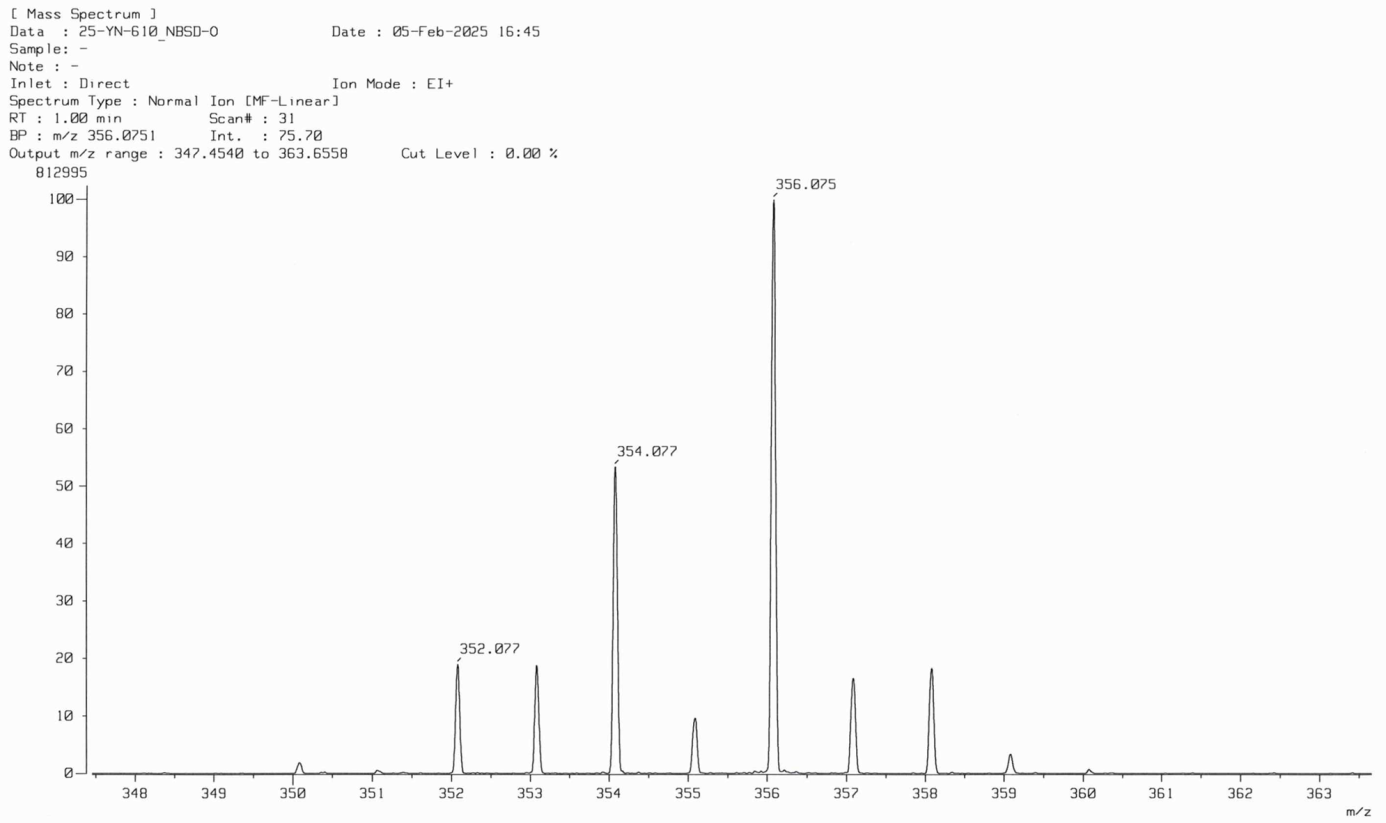


^1^H-NMR of **NBSD-aniline**

**
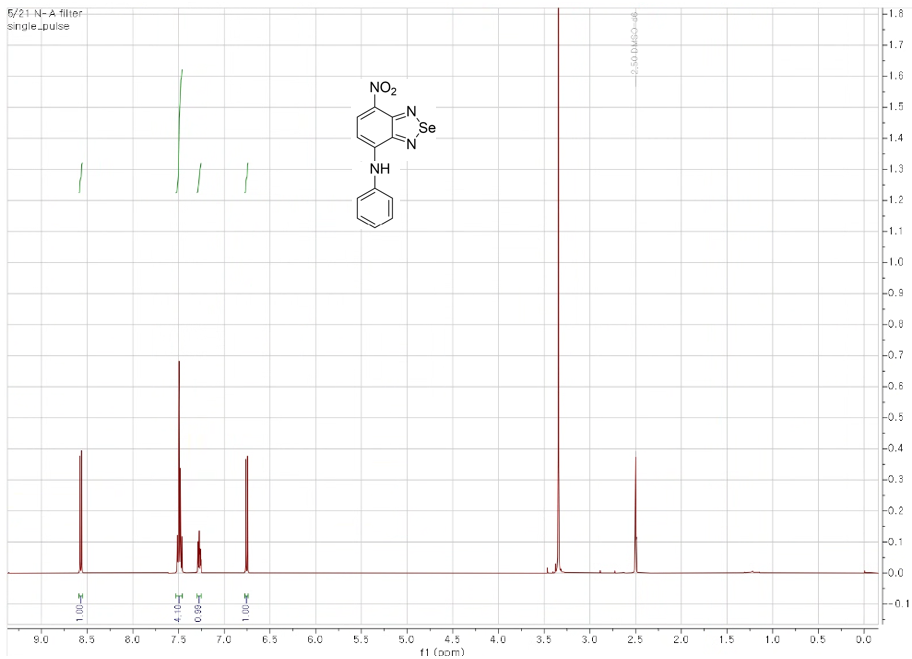
**

Mass of **NBSD-anilne**

**
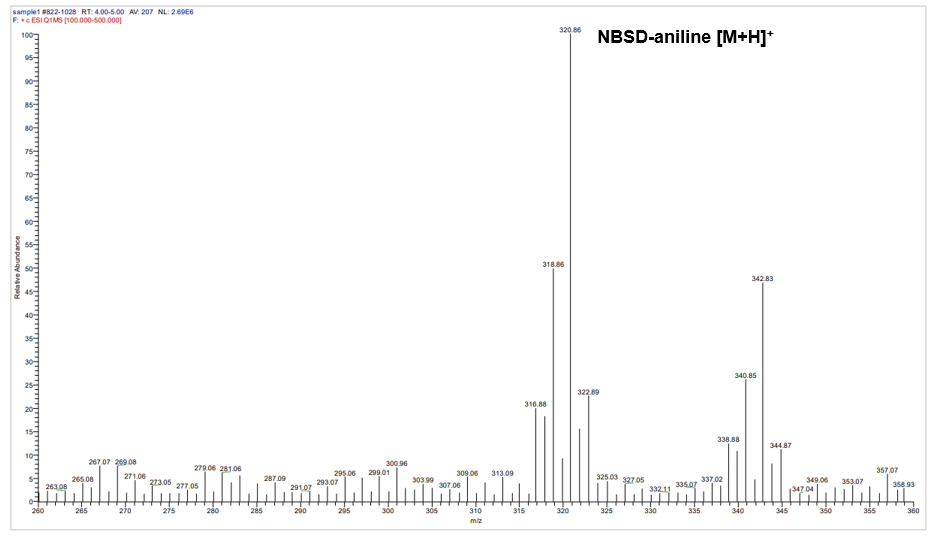
**

**[Supporting Tables]**

**Table S1**. Aggregate size (nm) and PDI of the NBSD series. All values represent the average (n=3). DI H_2_O: deionized water. Ace: acetone, EtOH: ethanol. N.D.: not detected.

| **Binary solvent** | **NBSD-NM** | | **NBSD-NP** | | **NBSD-NOc** | |
| --- | --- | --- | --- | --- | --- | --- |
|  | **Size** | **PDI** | **Size** | **PDI** | **Size** | **PDI** |
| 10%  DI H_2_O/Ace | 182.2 | 0.248 | 316.7 | 0.39 | 1091 | 1.0 |
| 50%  DI H_2_O/Ace | 316.7 | 0.38 | 373.4 | 0.31 | 390.6 | 0.30 |
| 90%  DI H_2_O/Ace | 808.1 | 0.82 | 375.6 | 0.55 | 414.3 | 0.46 |
| 10%  DI H_2_O/EtOH | N.D. | N.D. | 505.4 | 0.78 | N.D. | N.D. |
| 50%  DI H_2_O/EtOH | N.D. | N.D. | 198.1 | 0.417 | N.D. | N.D. |
| 90%  DI H_2_O/EtOH | N.D. | N.D. | 120.0 | 0.352 | 804.5 | 0.636 |

**[Crystallographic Data]**

**Crystallographic data of NBSD-NM**

The crystal structure was deposited at the Cambridge Crystallographic Data Center (CCDC).

CCDC deposition number: 2421472

**Table S2**. Crystal data and structure refinement for NBSD-NM.

Identification code **NBSD-NM**

Empirical formula C7 H6 N4 O2 Se

Formula weight 257.12

Temperature 223(2) K

Wavelength 0.71073 Å

Crystal system Monoclinic

Space group P2**_1_**/c

Unit cell dimensions a = 3.837(3) Å a= 90°.

b = 17.638(14) Å b= 90.91(2)°.

c = 12.389(10) Å g = 90°.

Volume 838.4(11) Å3

Z 4

Density (calculated) 2.037 Mg/m3

Absorption coefficient 4.455 mm-1

F(000) 504

Crystal size 0.124 x 0.070 x 0.036 mm3

Theta range for data collection 2.009 to 28.356°.

Index ranges -2<=h<=5, -23<=k<=23, -16<=l<=16

Reflections collected 8809

Independent reflections 2004 [R(int) = 0.1238]

Completeness to theta = 25.242° 96.6 %

Absorption correction Semi-empirical from equivalents

Max. and min. transmission 0.7457 and 0.5741

Refinement method Full-matrix least-squares on F2

Data / restraints / parameters 2004 / 0 / 128

Goodness-of-fit on F2 1.096

Final R indices [I>2sigma(I)] R1 = 0.0729, wR2 = 0.1508

R indices (all data) R1 = 0.1492, wR2 = 0.1780

Extinction coefficient n/a

Largest diff. peak and hole 1.141 and -0.947 e.Å-3

**Table S3**. Atomic coordinates (×104) and equivalent isotropic displacement parameters (Å2 × 103) for NBSD-NM. U(eq) is defined as one-third of the trace of the orthogonalized Uij tensor.

**x y z U(eq)**

Se(1) 10719(3) 5928(1) 5735(1) 32(1)

O(1) 5750(20) 5134(3) 2590(5) 57(2)

O(2) 2990(20) 5890(3) 1511(5) 53(2)

N(1) 10131(19) 6918(4) 5532(5) 34(2)

N(2) 8730(20) 5684(4) 4458(5) 30(2)

N(3) 4880(20) 5770(4) 2325(6) 36(2)

N(4) 8560(20) 8367(3) 4741(6) 37(2)

C(1) 8570(20) 7013(4) 4575(6) 28(2)

C(2) 7770(20) 6324(4) 3978(7) 29(2)

C(3) 5950(20) 6407(4) 2956(7) 28(2)

C(4) 5190(20) 7126(4) 2591(6) 32(2)

C(5) 6020(20) 7790(4) 3138(6) 32(2)

C(6) 7670(20) 7758(4) 4144(6) 28(2)

C(7) 7890(30) 9137(4) 4382(8) 50(3)

**Table S4**. Bond lengths [Å] and bond angles [°] for NBSD-NM.

Se(1)-N(1) 1.778(7)

Se(1)-N(2) 1.797(7)

O(1)-N(3) 1.215(8)

O(2)-N(3) 1.250(9)

N(1)-C(1) 1.331(9)

N(2)-C(2) 1.325(10)

N(3)-C(3) 1.426(10)

N(4)-C(6) 1.346(10)

N(4)-C(7) 1.451(10)

N(4)-H(4N) 0.8700

C(1)-C(2) 1.452(10)

C(1)-C(6) 1.457(10)

C(2)-C(3) 1.444(11)

C(3)-C(4) 1.376(10)

C(4)-C(5) 1.388(11)

C(4)-H(4) 0.9400

C(5)-C(6) 1.389(10)

C(5)-H(5) 0.9400

C(7)-H(7A) 0.9700

C(7)-H(7B) 0.9700

C(7)-H(7C) 0.9700

N(1)-Se(1)-N(2) 93.4(3)

C(1)-N(1)-Se(1) 107.7(5)

C(2)-N(2)-Se(1) 107.6(5)

O(1)-N(3)-O(2) 121.9(7)

O(1)-N(3)-C(3) 120.2(7)

O(2)-N(3)-C(3) 117.8(7)

C(6)-N(4)-C(7) 122.5(7)

C(6)-N(4)-H(4N) 118.7

C(7)-N(4)-H(4N) 118.7

N(1)-C(1)-C(2) 115.9(7)

N(1)-C(1)-C(6) 122.7(7)

C(2)-C(1)-C(6) 121.4(7)

N(2)-C(2)-C(3) 127.4(7)

N(2)-C(2)-C(1) 115.4(7)

C(3)-C(2)-C(1) 117.2(7)

C(4)-C(3)-N(3) 119.2(7)

C(4)-C(3)-C(2) 118.6(7)

N(3)-C(3)-C(2) 122.2(7)

C(3)-C(4)-C(5) 124.8(7)

C(3)-C(4)-H(4) 117.6

C(5)-C(4)-H(4) 117.6

C(4)-C(5)-C(6) 120.1(7)

C(4)-C(5)-H(5) 120.0

C(6)-C(5)-H(5) 120.0

N(4)-C(6)-C(5) 124.6(7)

N(4)-C(6)-C(1) 117.6(7)

C(5)-C(6)-C(1) 117.9(7)

N(4)-C(7)-H(7A) 109.5

N(4)-C(7)-H(7B) 109.5

H(7A)-C(7)-H(7B) 109.5

N(4)-C(7)-H(7C) 109.5

H(7A)-C(7)-H(7C) 109.5

H(7B)-C(7)-H(7C) 109.5

Symmetry transformations used to generate equivalent atoms

**Table S5**. Anisotropic displacement parameters (Å2 × 103) for NBSD-NM. The anisotropic displacement factor exponent takes the form: -2p2[h2 a*2U11 + ... + 2 h k a* b* U12]

**U11 U22 U33 U23 U13 U12**

Se(1) 30(1) 30(1) 35(1) 1(1) -8(1) 2(1)

O(1) 85(6) 28(3) 58(4) -2(3) -36(4) 5(3)

O(2) 63(6) 54(4) 41(4) -1(3) -26(4) -6(4)

N(1) 27(5) 38(4) 38(4) 1(3) -4(4) 5(3)

N(2) 28(5) 28(3) 32(4) 2(3) 3(3) 4(3)

N(3) 38(5) 33(4) 36(4) -5(3) -5(4) 0(3)

N(4) 39(5) 26(4) 45(4) 1(3) -15(4) -3(3)

C(1) 12(5) 33(4) 38(5) 1(3) 3(4) -1(3)

C(2) 25(6) 22(4) 41(5) 0(3) 2(4) 2(3)

C(3) 21(5) 30(4) 34(4) -1(3) 2(4) -2(3)

C(4) 31(6) 36(4) 29(4) 5(3) -6(4) 2(4)

C(5) 30(6) 30(4) 35(5) 3(3) -4(4) 6(4)

C(6) 19(5) 32(4) 34(4) 0(3) 3(4) 1(3)

C(7) 56(7) 29(5) 65(6) 6(4) -21(6) 3(4)

**Table S6.** Hydrogen coordinates (× 104) and isotropic displacement parameters (Å2 × 103) for NBSD-NM.

**x y z U(eq)**

H(4N) 9573 8297 5366 44

H(4) 4023 7171 1921 38

H(5) 5467 8262 2829 38

H(7A) 9099 9230 3713 76

H(7B) 8715 9491 4929 76

H(7C) 5404 9207 4265 76

**Crystallographic data of NBSD-NP**

The crystal structure was deposited at the Cambridge Crystallographic Data Center (CCDC).

CCDC deposition number: 2421477

**Table S7**. Crystal data and structure refinement for NBSD-NP.

Identification code NBSD-NP

Empirical formula C9 H10 N4 O2 Se

Formula weight 285.17

Temperature 223(2) K

Wavelength 0.71073 Å

Crystal system Triclinic

Space group P-1

Unit cell dimensions a = 7.380(5) Å a= 77.321(14)°.

b = 8.667(6) Å b= 78.791(14)°.

c = 9.170(6) Å g = 68.106(14)°.

Volume 526.8(6) Å3

Z 2

Density (calculated) 1.798 Mg/m3

Absorption coefficient 3.555 mm-1

F(000) 284

Crystal size 0.075 x 0.060 x 0.033 mm3

Theta range for data collection 2.566 to 26.128°.

Index ranges -9<=h<=9, -10<=k<=10, -11<=l<=11

Reflections collected 5329

Independent reflections 2052 [R(int) = 0.0736]

Completeness to theta = 25.242° 98.4 %

Absorption correction Semi-empirical from equivalents

Max. and min. transmission 0.7453 and 0.6180

Refinement method Full-matrix least-squares on F2

Data / restraints / parameters 2052 / 0 / 146

Goodness-of-fit on F2 1.047

Final R indices [I>2sigma(I)] R1 = 0.1015, wR2 = 0.2564

R indices (all data) R1 = 0.1589, wR2 = 0.3061

Extinction coefficient n/a

Largest diff. peak and hole 1.934 and -1.019 e.Å-3

**Table S8**. Atomic coordinates (× 104) and equivalent isotropic displacement parameters (Å2 × 103) for NBSD-NP. U(eq) is defined as one-third of the trace of the orthogonalized Uij tensor.

x y z U(eq)

Se(1) 6926(2) 3302(1) 8960(1) 57(1)

O(1) 3122(13) 8795(11) 7777(9) 67(2)

O(2) 2522(13) 9854(11) 5472(9) 71(3)

N(1) 8174(14) 2999(13) 7122(10) 57(3)

N(2) 5395(13) 5451(11) 8351(9) 48(2)

N(3) 3464(14) 8741(12) 6407(11) 52(2)

N(4) 9471(13) 3276(13) 4025(10) 50(2)

C(1) 7422(16) 4438(15) 6188(12) 50(3)

C(2) 5913(15) 5768(15) 6898(12) 49(3)

C(3) 5008(15) 7290(14) 5880(12) 44(2)

C(4) 5683(16) 7361(15) 4378(12) 49(3)

C(5) 7116(17) 6075(15) 3727(13) 50(3)

C(6) 8066(16) 4631(15) 4566(13) 47(3)

C(7) 10122(16) 3239(14) 2412(12) 48(3)

C(8) 11582(17) 1553(17) 2110(13) 59(3)

C(9) 12260(18) 1580(20) 418(14) 79(4)

**Table S9.** Bond lengths [Å] and bond angles [°] for NBSD-NP.

Se(1)-N(1) 1.780(9)

Se(1)-N(2) 1.810(9)

O(1)-N(3) 1.240(12)

O(2)-N(3) 1.232(12)

N(1)-C(1) 1.340(15)

N(2)-C(2) 1.311(13)

N(3)-C(3) 1.448(14)

N(4)-C(6) 1.359(14)

N(4)-C(7) 1.468(13)

N(4)-H(4N) 0.8700

C(1)-C(2) 1.443(16)

C(1)-C(6) 1.464(15)

C(2)-C(3) 1.450(15)

C(3)-C(4) 1.367(14)

C(4)-C(5) 1.372(16)

C(4)-H(4) 0.9400

C(5)-C(6) 1.335(16)

C(5)-H(5) 0.9400

C(7)-C(8) 1.504(15)

C(7)-H(7A) 0.9800

C(7)-H(7AB) 0.9800

C(8)-C(9) 1.533(16)

C(8)-H(8A) 0.9800

C(8)-H(8AB) 0.9800

C(9)-H(9A) 0.9700

C(9)-H(9B) 0.9700

C(9)-H(9C) 0.9700

N(1)-Se(1)-N(2) 94.0(4)

C(1)-N(1)-Se(1) 107.2(7)

C(2)-N(2)-Se(1) 105.8(7)

O(2)-N(3)-O(1) 122.9(9)

O(2)-N(3)-C(3) 118.3(10)

O(1)-N(3)-C(3) 118.8(9)

C(6)-N(4)-C(7) 123.0(10)

C(6)-N(4)-H(4N) 118.5

C(7)-N(4)-H(4N) 118.5

N(1)-C(1)-C(2) 115.0(10)

N(1)-C(1)-C(6) 122.3(10)

C(2)-C(1)-C(6) 122.7(11)

N(2)-C(2)-C(1) 118.0(10)

N(2)-C(2)-C(3) 126.6(10)

C(1)-C(2)-C(3) 115.2(10)

C(4)-C(3)-N(3) 119.3(10)

C(4)-C(3)-C(2) 118.3(10)

N(3)-C(3)-C(2) 122.3(9)

C(3)-C(4)-C(5) 125.3(11)

C(3)-C(4)-H(4) 117.3

C(5)-C(4)-H(4) 117.3

C(6)-C(5)-C(4) 121.1(11)

C(6)-C(5)-H(5) 119.5

C(4)-C(5)-H(5) 119.5

C(5)-C(6)-N(4) 125.4(11)

C(5)-C(6)-C(1) 117.2(10)

N(4)-C(6)-C(1) 117.2(10)

N(4)-C(7)-C(8) 112.4(9)

N(4)-C(7)-H(7A) 109.1

C(8)-C(7)-H(7A) 109.1

N(4)-C(7)-H(7AB) 109.1

C(8)-C(7)-H(7AB) 109.1

H(7A)-C(7)-H(7AB) 107.9

C(7)-C(8)-C(9) 110.8(10)

C(7)-C(8)-H(8A) 109.5

C(9)-C(8)-H(8A) 109.5

C(7)-C(8)-H(8AB) 109.5

C(9)-C(8)-H(8AB) 109.5

H(8A)-C(8)-H(8AB) 108.1

C(8)-C(9)-H(9A) 109.5

C(8)-C(9)-H(9B) 109.5

H(9A)-C(9)-H(9B) 109.5

C(8)-C(9)-H(9C) 109.5

H(9A)-C(9)-H(9C) 109.5

H(9B)-C(9)-H(9C) 109.5

Symmetry transformations used to generate equivalent atoms

**Table S10.** Anisotropic displacement parameters (Å2 × 103) for NBSD-NP. The anisotropic

displacement factor exponent takes the form: -2p2[h2 a*2U11 + ... + 2 h k a* b* U12]

**U11 U22 U33 U23 U13 U12**

Se(1) 59(1) 59(1) 37(1) 0(1) -3(1) -7(1)

O(1) 77(6) 63(5) 35(4) -7(4) -7(4) 3(4)

O(2) 83(6) 54(5) 39(5) 3(4) -3(4) 11(4)

N(1) 55(5) 61(6) 32(5) 1(5) 4(4) -5(4)

N(2) 58(5) 49(5) 29(5) 0(4) -1(4) -16(4)

N(3) 56(5) 41(5) 51(6) -4(5) -8(5) -11(4)

N(4) 48(5) 70(6) 29(4) -12(5) -3(4) -14(5)

C(1) 50(6) 64(7) 35(6) -9(6) -8(5) -17(5)

C(2) 44(5) 64(7) 35(6) -2(5) -14(5) -14(5)

C(3) 50(6) 46(6) 33(5) -3(5) -11(4) -13(5)

C(4) 53(6) 54(7) 35(6) 1(5) -8(5) -18(5)

C(5) 63(7) 51(7) 35(6) -5(5) -6(5) -21(6)

C(6) 48(6) 57(7) 42(6) -9(6) -7(5) -24(5)

C(7) 47(6) 53(7) 39(6) -5(5) -10(5) -9(5)

C(8) 54(6) 71(8) 39(6) -18(6) 5(5) -8(6)

C(9) 52(7) 110(11) 40(7) -8(7) -8(6) 11(7)

**Table S11.** Hydrogen coordinates (× 104) and isotropic displacement parameters (Å2 × 103)

for NBSD-NP.

**x y z U(eq)**

H(4N) 10010 2390 4665 61

H(4) 5120 8373 3738 58

H(5) 7433 6213 2674 59

H(7A) 10721 4106 2001 58

H(7AB) 8974 3510 1890 58

H(8A) 10977 686 2493 71

H(8AB) 12723 1268 2643 71

H(9A) 13547 719 268 118

H(9B) 12336 2675 -41 118

H(9C) 11326 1352 -42 118

**Crystallographic data of NBSD-NOc**

The crystal structure was deposited at the Cambridge Crystallographic Data Center (CCDC).

CCDC deposition number: 2421479

**Table S12**. Crystal data and structure refinement for NBSD-NOc.

Identification code NBSD-NOc

Empirical formula C14 H20 N4 O2 Se

Formula weight 355.30

Temperature 223(2) K

Wavelength 0.71073 Å

Crystal system Triclinic

Space group P-1

Unit cell dimensions a = 4.6609(19) Å a= 111.975(9)°.

b = 18.922(8) Å b= 93.786(10)°.

c = 21.451(9) Å g = 96.564(10)°.

Volume 1730.7(13) Å3

Z 4

Density (calculated) 1.364 Mg/m3

Absorption coefficient 2.179 mm-1

F(000) 728

Crystal size 0.232 x 0.045 x 0.035 mm3

Theta range for data collection 2.348 to 25.902°.

Index ranges -5<=h<=5, -23<=k<=23, -26<=l<=26

Reflections collected 25202

Independent reflections 6607 [R(int) = 0.2473]

Completeness to theta = 25.242° 98.9 %

Absorption correction Semi-empirical from equivalents

Max. and min. transmission 0.7453 and 0.5412

Refinement method Full-matrix least-squares on F2

Data / restraints / parameters 6607 / 12 / 382

Goodness-of-fit on F2 1.007

Final R indices [I>2sigma(I)] R1 = 0.1017, wR2 = 0.2222

R indices (all data) R1 = 0.2573, wR2 = 0.2845

Extinction coefficient 0.0119(19)

Largest diff. peak and hole 0.814 and -0.746 e.Å-3

**Table S13**. Atomic coordinates (× 104) and equivalent isotropic displacement parameters (Å2 × 103) for NBSD-NOc. U(eq) is defined as one-third of the trace of the orthogonalized Uij tensor.

**x y z U(eq)**

Se(1) -2408(3) 5731(1) 4836(1) 48(1)

Se(2) 8162(3) 10364(1) 5801(1) 64(1)

O(1) -5700(20) 3185(4) 3832(5) 68(3)

O(2) -4490(20) 2469(5) 2889(5) 72(3)

O(3) 8300(30) 8102(5) 4070(5) 98(4)

O(4) 6000(30) 7056(6) 3992(6) 110(5)

N(1) -3570(20) 4725(5) 4396(5) 43(3)

N(2) 100(20) 5773(6) 4271(5) 46(3)

N(3) -4310(30) 3090(7) 3357(6) 53(3)

N(4) 3580(20) 5482(5) 3222(5) 51(3)

N(5) 8050(20) 9442(5) 5139(5) 53(3)

N(6) 5540(20) 10028(6) 6249(6) 65(3)

N(7) 6680(30) 7738(6) 4282(7) 85(5)

N(8) 1530(20) 9176(6) 6646(7) 63(4)

C(1) -2160(30) 4473(6) 3855(6) 40(3)

C(2) -2270(30) 3725(6) 3355(7) 45(3)

C(3) -650(30) 3606(7) 2841(6) 47(3)

C(4) 1240(30) 4170(7) 2774(6) 52(3)

C(5) 1650(30) 4914(6) 3262(6) 40(3)

C(6) -110(30) 5069(6) 3793(6) 39(3)

C(7) 5360(30) 5492(7) 2708(6) 52(4)

C(8) 3630(30) 5659(7) 2175(6) 62(4)

C(9) 5350(30) 5808(8) 1642(8) 75(5)

C(10) 3610(30) 5919(9) 1080(7) 77(5)

C(11) 5370(30) 6123(9) 618(7) 74(5)

C(12) 3490(30) 6203(9) 37(7) 78(5)

C(13) 5200(40) 6393(9) -486(8) 87(5)

C(14) 3360(30) 6465(9) -1031(8) 92(6)

C(15) 6190(30) 8944(7) 5268(8) 59(4)

C(16) 5390(30) 8147(7) 4897(7) 51(4)

C(17) 3290(30) 7716(7) 5121(7) 59(4)

C(18) 2050(30) 8045(7) 5695(8) 59(4)

C(19) 2670(30) 8832(8) 6104(9) 66(5)

C(20) 4720(30) 9273(7) 5871(8) 60(4)

C(21) -510(30) 8782(8) 6963(7) 62(4)

C(22) -1400(30) 9368(8) 7576(8) 72(4)

C(23) 930(30) 9739(8) 8141(8) 76(5)

C(24) -180(30) 10269(9) 8780(8) 87(5)

C(25) 2130(40) 10640(9) 9400(8) 90(5)

C(26) 920(40) 11122(9) 10007(8) 103(6)

C(27) 3100(30) 11482(9) 10649(8) 84(5)

C(28) 1830(40) 11975(9) 11261(8) 95(6)

**Table S14**. Bond lengths [Å] and band angles [°] for NBSD-NOc.

Se(1)-N(2) 1.754(9)

Se(1)-N(1) 1.777(9)

Se(2)-N(5) 1.783(10)

Se(2)-N(6) 1.805(11)

O(1)-N(3) 1.214(12)

O(2)-N(3) 1.215(12)

O(3)-N(7) 1.190(13)

O(4)-N(7) 1.197(12)

N(1)-C(1) 1.331(14)

N(2)-C(6) 1.330(13)

N(3)-C(2) 1.445(14)

N(4)-C(5) 1.353(13)

N(4)-C(7) 1.426(14)

N(4)-H(4) 0.8700

N(5)-C(15) 1.323(14)

N(6)-C(20) 1.346(14)

N(7)-C(16) 1.467(17)

N(8)-C(19) 1.285(17)

N(8)-C(21) 1.493(15)

N(8)-H(8) 0.8700

C(1)-C(2) 1.414(15)

C(1)-C(6) 1.443(14)

C(2)-C(3) 1.341(15)

C(3)-C(4) 1.360(15)

C(3)-H(3) 0.9400

C(4)-C(5) 1.387(15)

C(4)-H(4A) 0.9400

C(5)-C(6) 1.409(15)

C(7)-C(8) 1.503(16)

C(7)-H(7A) 0.9800

C(7)-H(7AB) 0.9800

C(8)-C(9) 1.530(16)

C(8)-H(8A) 0.9800

C(8)-H(8AB) 0.9800

C(9)-C(10) 1.502(17)

C(9)-H(9A) 0.9800

C(9)-H(9AB) 0.9800

C(10)-C(11) 1.461(16)

C(10)-H(10A) 0.9800

C(10)-H(10B) 0.9800

C(11)-C(12) 1.539(16)

C(11)-H(11A) 0.9800

C(11)-H(11B) 0.9800

C(12)-C(13) 1.539(18)

C(12)-H(12A) 0.9800

C(12)-H(12B) 0.9800

C(13)-C(14) 1.460(18)

C(13)-H(13A) 0.9800

C(13)-H(13B) 0.9800

C(14)-H(14A) 0.9700

C(14)-H(14B) 0.9700

C(14)-H(14C) 0.9700

C(15)-C(16) 1.408(16)

C(15)-C(20) 1.463(18)

C(16)-C(17) 1.422(16)

C(17)-C(18) 1.358(17)

C(17)-H(17) 0.9400

C(18)-C(19) 1.400(17)

C(18)-H(18) 0.9400

C(19)-C(20) 1.433(19)

C(21)-C(22) 1.488(17)

C(21)-H(21A) 0.9800

C(21)-H(21B) 0.9800

C(22)-C(23) 1.469(18)

C(22)-H(22A) 0.9800

C(22)-H(22B) 0.9800

C(23)-C(24) 1.528(18)

C(23)-H(23A) 0.9800

C(23)-H(23B) 0.9800

C(24)-C(25) 1.535(19)

C(24)-H(24A) 0.9800

C(24)-H(24B) 0.9800

C(25)-C(26) 1.47(2)

C(25)-H(25A) 0.9800

C(25)-H(25B) 0.9800

C(26)-C(27) 1.53(2)

C(26)-H(26A) 0.9800

C(26)-H(26B) 0.9800

C(27)-C(28) 1.501(19)

C(27)-H(27A) 0.9800

C(27)-H(27B) 0.9800

C(28)-H(28A) 0.9700

C(28)-H(28B) 0.9700

C(28)-H(28C) 0.9700

N(2)-Se(1)-N(1) 93.5(5)

N(5)-Se(2)-N(6) 94.6(5)

C(1)-N(1)-Se(1) 108.8(7)

C(6)-N(2)-Se(1) 107.5(8)

O(1)-N(3)-O(2) 122.3(11)

O(1)-N(3)-C(2) 119.3(12)

O(2)-N(3)-C(2) 118.3(12)

C(5)-N(4)-C(7) 131.1(11)

C(5)-N(4)-H(4) 114.4

C(7)-N(4)-H(4) 114.4

C(15)-N(5)-Se(2) 107.7(10)

C(20)-N(6)-Se(2) 105.4(10)

O(3)-N(7)-O(4) 121.6(15)

O(3)-N(7)-C(16) 118.5(11)

O(4)-N(7)-C(16) 119.8(12)

C(19)-N(8)-C(21) 124.6(12)

C(19)-N(8)-H(8) 117.7

C(21)-N(8)-H(8) 117.7

N(1)-C(1)-C(2) 130.7(11)

N(1)-C(1)-C(6) 113.3(10)

C(2)-C(1)-C(6) 116.0(12)

C(3)-C(2)-C(1) 120.3(11)

C(3)-C(2)-N(3) 118.9(11)

C(1)-C(2)-N(3) 120.6(12)

C(2)-C(3)-C(4) 123.8(13)

C(2)-C(3)-H(3) 118.1

C(4)-C(3)-H(3) 118.1

C(3)-C(4)-C(5) 120.4(13)

C(3)-C(4)-H(4A) 119.8

C(5)-C(4)-H(4A) 119.8

N(4)-C(5)-C(4) 121.9(12)

N(4)-C(5)-C(6) 120.6(10)

C(4)-C(5)-C(6) 117.5(11)

N(2)-C(6)-C(5) 121.2(10)

N(2)-C(6)-C(1) 116.9(11)

C(5)-C(6)-C(1) 121.9(11)

N(4)-C(7)-C(8) 109.5(11)

N(4)-C(7)-H(7A) 109.8

C(8)-C(7)-H(7A) 109.8

N(4)-C(7)-H(7AB) 109.8

C(8)-C(7)-H(7AB) 109.8

H(7A)-C(7)-H(7AB) 108.2

C(7)-C(8)-C(9) 115.9(12)

C(7)-C(8)-H(8A) 108.3

C(9)-C(8)-H(8A) 108.3

C(7)-C(8)-H(8AB) 108.3

C(9)-C(8)-H(8AB) 108.3

H(8A)-C(8)-H(8AB) 107.4

C(10)-C(9)-C(8) 116.1(12)

C(10)-C(9)-H(9A) 108.3

C(8)-C(9)-H(9A) 108.3

C(10)-C(9)-H(9AB) 108.3

C(8)-C(9)-H(9AB) 108.3

H(9A)-C(9)-H(9AB) 107.4

C(11)-C(10)-C(9) 113.9(12)

C(11)-C(10)-H(10A) 108.8

C(9)-C(10)-H(10A) 108.8

C(11)-C(10)-H(10B) 108.8

C(9)-C(10)-H(10B) 108.8

H(10A)-C(10)-H(10B) 107.7

C(10)-C(11)-C(12) 112.1(11)

C(10)-C(11)-H(11A) 109.2

C(12)-C(11)-H(11A) 109.2

C(10)-C(11)-H(11B) 109.2

C(12)-C(11)-H(11B) 109.2

H(11A)-C(11)-H(11B) 107.9

C(13)-C(12)-C(11) 115.3(12)

C(13)-C(12)-H(12A) 108.5

C(11)-C(12)-H(12A) 108.5

C(13)-C(12)-H(12B) 108.5

C(11)-C(12)-H(12B) 108.5

H(12A)-C(12)-H(12B) 107.5

C(14)-C(13)-C(12) 114.0(14)

C(14)-C(13)-H(13A) 108.7

C(12)-C(13)-H(13A) 108.7

C(14)-C(13)-H(13B) 108.7

C(12)-C(13)-H(13B) 108.7

H(13A)-C(13)-H(13B) 107.6

C(13)-C(14)-H(14A) 109.5

C(13)-C(14)-H(14B) 109.5

H(14A)-C(14)-H(14B) 109.5

C(13)-C(14)-H(14C) 109.5

H(14A)-C(14)-H(14C) 109.5

H(14B)-C(14)-H(14C) 109.5

N(5)-C(15)-C(16) 128.9(16)

N(5)-C(15)-C(20) 115.4(12)

C(16)-C(15)-C(20) 115.6(12)

C(15)-C(16)-C(17) 119.9(14)

C(15)-C(16)-N(7) 121.8(12)

C(17)-C(16)-N(7) 118.2(11)

C(18)-C(17)-C(16) 122.2(12)

C(18)-C(17)-H(17) 118.9

C(16)-C(17)-H(17) 118.9

C(17)-C(18)-C(19) 123.0(13)

C(17)-C(18)-H(18) 118.5

C(19)-C(18)-H(18) 118.5

N(8)-C(19)-C(18) 125.8(14)

N(8)-C(19)-C(20) 119.2(13)

C(18)-C(19)-C(20) 115.1(16)

N(6)-C(20)-C(15) 116.7(12)

N(6)-C(20)-C(19) 119.0(16)

C(15)-C(20)-C(19) 124.1(13)

C(22)-C(21)-N(8) 109.2(12)

C(22)-C(21)-H(21A) 109.8

N(8)-C(21)-H(21A) 109.8

C(22)-C(21)-H(21B) 109.8

N(8)-C(21)-H(21B) 109.8

H(21A)-C(21)-H(21B) 108.3

C(23)-C(22)-C(21) 114.7(13)

C(23)-C(22)-H(22A) 108.6

C(21)-C(22)-H(22A) 108.6

C(23)-C(22)-H(22B) 108.6

C(21)-C(22)-H(22B) 108.6

H(22A)-C(22)-H(22B) 107.6

C(22)-C(23)-C(24) 112.3(13)

C(22)-C(23)-H(23A) 109.2

C(24)-C(23)-H(23A) 109.2

C(22)-C(23)-H(23B) 109.2

C(24)-C(23)-H(23B) 109.2

H(23A)-C(23)-H(23B) 107.9

C(23)-C(24)-C(25) 114.6(14)

C(23)-C(24)-H(24A) 108.6

C(25)-C(24)-H(24A) 108.6

C(23)-C(24)-H(24B) 108.6

C(25)-C(24)-H(24B) 108.6

H(24A)-C(24)-H(24B) 107.6

C(26)-C(25)-C(24) 112.5(15)

C(26)-C(25)-H(25A) 109.1

C(24)-C(25)-H(25A) 109.1

C(26)-C(25)-H(25B) 109.1

C(24)-C(25)-H(25B) 109.1

H(25A)-C(25)-H(25B) 107.8

C(25)-C(26)-C(27) 114.8(16)

C(25)-C(26)-H(26A) 108.6

C(27)-C(26)-H(26A) 108.6

C(25)-C(26)-H(26B) 108.6

C(27)-C(26)-H(26B) 108.6

H(26A)-C(26)-H(26B) 107.6

C(28)-C(27)-C(26) 113.9(14)

C(28)-C(27)-H(27A) 108.8

C(26)-C(27)-H(27A) 108.8

C(28)-C(27)-H(27B) 108.8

C(26)-C(27)-H(27B) 108.8

H(27A)-C(27)-H(27B) 107.7

C(27)-C(28)-H(28A) 109.5

C(27)-C(28)-H(28B) 109.5

H(28A)-C(28)-H(28B) 109.5

C(27)-C(28)-H(28C) 109.5

H(28A)-C(28)-H(28C) 109.5

H(28B)-C(28)-H(28C) 109.5

Symmetry transformations used to generate equivalent atoms

**Table S15**. Anisotropic displacement parameters (Å2 × 103) for NBSD-NOc. The anisotropic displacement factor exponent takes the form: -2p2[h2 a*2U11 + ... + 2 h k a* b* U12]

**U11 U22 U33 U23 U13 U12**

Se(1) 42(1) 45(1) 54(1) 19(1) 11(1) 0(1)

Se(2) 52(1) 46(1) 93(1) 32(1) 3(1) -8(1)

O(1) 85(8) 36(5) 79(8) 21(5) 22(6) -10(5)

O(2) 97(9) 41(5) 59(7) 1(5) 16(6) -1(6)

O(3) 133(11) 58(6) 91(8) 22(6) 48(8) -31(7)

O(4) 135(11) 55(7) 111(10) 5(6) 56(8) -33(7)

N(1) 36(6) 46(6) 41(7) 10(5) 21(5) 5(5)

N(2) 46(7) 57(7) 43(7) 27(6) 20(6) 10(6)

N(3) 56(8) 59(8) 59(8) 33(7) 32(7) 14(7)

N(4) 44(7) 45(6) 60(8) 17(6) 21(6) -1(5)

N(5) 51(7) 41(6) 65(8) 25(6) -1(6) -8(5)

N(6) 48(8) 61(7) 103(10) 53(7) 6(7) -2(6)

N(7) 92(11) 36(7) 114(13) 27(8) 3(10) -24(7)

N(8) 44(8) 49(7) 97(11) 29(7) 20(7) -2(6)

C(1) 34(8) 39(7) 48(8) 21(7) -4(7) 2(6)

C(2) 39(8) 32(7) 57(9) 13(7) 3(7) -5(6)

C(3) 44(8) 55(8) 52(9) 34(7) 15(7) 0(7)

C(4) 33(8) 74(9) 43(8) 14(8) 1(7) 12(7)

C(5) 38(8) 36(7) 33(7) 6(6) -10(6) -16(6)

C(6) 35(8) 37(7) 48(8) 22(7) 16(7) -3(6)

C(7) 45(9) 76(9) 39(8) 28(7) 6(7) 1(7)

C(8) 55(10) 69(9) 54(9) 21(8) 1(8) -11(8)

C(9) 51(10) 88(11) 103(13) 53(10) 30(10) 7(8)

C(10) 72(12) 118(13) 65(11) 64(10) 6(9) 2(10)

C(11) 39(9) 120(13) 91(12) 68(11) 21(9) 21(9)

C(12) 41(9) 134(14) 76(11) 61(11) 7(9) 2(9)

C(13) 93(14) 92(12) 80(12) 35(10) 24(11) 11(10)

C(14) 87(13) 131(14) 99(13) 88(12) 5(11) 29(11)

C(15) 42(9) 47(8) 102(13) 47(9) -5(9) 2(7)

C(16) 57(10) 35(7) 50(9) 9(7) 6(8) -5(7)

C(17) 62(11) 26(7) 81(12) 20(7) -17(9) -12(7)

C(18) 50(9) 41(8) 94(12) 34(8) 26(9) 3(7)

C(19) 51(11) 48(9) 104(14) 42(10) -20(10) -7(8)

C(20) 56(10) 32(7) 96(13) 40(8) -22(9) -13(7)

C(21) 51(10) 71(10) 74(11) 42(9) 1(8) 2(8)

C(22) 41(9) 76(10) 90(13) 23(10) 10(9) -2(8)

C(23) 70(12) 72(10) 77(12) 21(9) 20(10) -4(9)

C(24) 55(11) 109(13) 86(13) 28(11) 5(10) 13(10)

C(25) 86(7) 94(6) 87(7) 30(5) 14(5) 16(5)

C(26) 100(7) 105(7) 103(7) 38(5) 11(5) 16(5)

C(27) 43(10) 117(13) 106(15) 56(12) 16(10) 9(10)

C(28) 80(13) 105(13) 68(12) -1(10) -10(10) 11(11)

**Table S16**. Hydrogen coordinates (× 104) and isotropic displacement parameters (Å2 × 103) for NBSD-NOc.

**x y z U(eq)**

H(4) 3771 5917 3571 61

H(8) 1975 9675 6841 76

H(3) -824 3105 2509 56

H(4A) 2264 4055 2394 62

H(7A) 6024 4992 2503 63

H(7AB) 7068 5888 2907 63

H(8A) 2693 6110 2401 74

H(8AB) 2085 5221 1942 74

H(9A) 6804 6269 1872 90

H(9AB) 6409 5373 1441 90

H(10A) 2278 5441 821 93

H(10B) 2426 6325 1279 93

H(11A) 6603 5726 427 89

H(11B) 6653 6612 871 89

H(12A) 2165 5720 -200 94

H(12B) 2307 6610 233 94

H(13A) 6520 6879 -252 105

H(13B) 6383 5988 -684 105

H(14A) 4533 6717 -1267 138

H(14B) 1854 6768 -842 138

H(14C) 2464 5956 -1346 138

H(17) 2755 7186 4863 71

H(18) 707 7730 5824 71

H(21A) -2232 8500 6639 75

H(21B) 444 8411 7088 75

H(22A) -2130 9767 7450 87

H(22B) -3001 9119 7731 87

H(23A) 2444 10041 8008 91

H(23B) 1813 9341 8241 91

H(24A) -1777 9971 8893 104

H(24B) -974 10679 8683 104

H(25A) 2969 10234 9494 108

H(25B) 3690 10958 9299 108

H(26A) -703 10806 10092 124

H(26B) 141 11537 9914 124

H(27A) 3874 11068 10745 101

H(27B) 4732 11797 10565 101

H(28A) 806 12337 11150 143

H(28B) 3385 12255 11625 143

H(28C) 492 11650 11404 143

**Table S17**. Animal study records of tumor size in Group 1 (non-treated). Tumor volume was calculated using the formula:

Volume = 0.5 × (long side) × (short side) × (height)

| Day | Group 1 | Long side (mm) | Short side (mm) | Height (mm) | Volume (mm^3^) | Average with S.D. |
| --- | --- | --- | --- | --- | --- | --- |
| 1 | 1 | 4.19 | 4.19 | 1.31 | 11.50 | 15.90 ± 5.19 |
|  | 2 | 5.95 | 3.4 | 1.12 | 11.33 |  |
|  | 3 | 4.27 | 7.52 | 1.47 | 23.60 |  |
|  | 4 | 4.66 | 5.9 | 1.53 | 21.03 |  |
|  | 5 | 4.87 | 3.99 | 1.53 | 14.86 |  |
|  | 6 | 3.79 | 6.5 | 1.06 | 13.06 |  |
| Day | Group 1 | Long side (mm) | Short side (mm) | Height (mm) | Volume (mm^3^) | Average with S.D. |
| 3 | 1 | 3.31 | 9.11 | 1 | 15.08 | 20.3 ± 5.9 |
|  | 2 | 2.73 | 9.74 | 1.64 | 21.80 |  |
|  | 3 | 5.72 | 5.71 | 1.61 | 26.29 |  |
|  | 4 | 3.79 | 6.59 | 1.27 | 15.86 |  |
|  | 5 | 4.05 | 7.73 | 1.56 | 24.42 |  |
|  | 6 | 4.2 | 9.04 | 1.4 | 26.58 |  |
|  | 7 | 3.77 | 3.64 | 1.76 | 12.08 |  |
| Day | Group 1 | Long side (mm) | Short side (mm) | Height (mm) | Volume (mm^3^) | Average with S.D. |
| 5 | 1 | 4.06 | 5.61 | 1.3 | 14.80 | 24.96 ± 8.59 |
|  | 2 | 6.04 | 5.91 | 1.64 | 29.27 |  |
|  | 3 | 4.17 | 7.51 | 2.18 | 34.14 |  |
|  | 4 | 4.63 | 5.03 | 1.57 | 18.28 |  |
|  | 5 | 9.15 | 3.55 | 1.1 | 17.87 |  |
|  | 6 | 4.19 | 8.57 | 2.05 | 36.81 |  |
|  | 7 | 4.63 | 7.49 | 1.36 | 23.58 |  |
| Day | Group1 | Long side (mm) | Short side (mm) | Height (mm) | Volume (mm^3^) | Average with S.D. |
| 7 | 1 | 6.98 | 6.51 | 2.05 | 46.58 | 48.97 ± 17.93 |
|  | 2 | 6.73 | 9.6 | 2.53 | 81.73 |  |
|  | 3 | 5 | 9.67 | 2.13 | 51.49 |  |
|  | 4 | 5.03 | 9.34 | 2.14 | 50.27 |  |
|  | 5 | 5.76 | 5.99 | 2.18 | 37.61 |  |
|  | 6 | 5.39 | 9.12 | 2.14 | 52.60 |  |
|  | 7 | 5.57 | 5.57 | 1.45 | 22.49 |  |
| Day | Group 1 | Long side (mm) | Short side (mm) | Height (mm) | Volume (mm^3^) | Average with S.D. |
| 9 | 1 | 6.66 | 12.05 | 2.57 | 103.13 | 77.93 ± 19.26 |
|  | 2 | 7.34 | 7.97 | 2.21 | 64.64 |  |
|  | 3 | 6.56 | 6.31 | 2.24 | 46.36 |  |
|  | 4 | 5.69 | 11.13 | 2.53 | 80.11 |  |
|  | 5 | 6.59 | 7.43 | 2.98 | 72.96 |  |
|  | 6 | 6.84 | 12.44 | 1.9 | 80.84 |  |
|  | 7 | 6.65 | 11.54 | 2.54 | 97.46 |  |
| Day | Group 1 | Long side (mm) | Short side (mm) | Height (mm) | Volume (mm^3^) | Average with S.D. |
| 11 | 1 | 8.97 | 9.96 | 3.6 | 160.81 | 130.67 ± 17.52 |
|  | 2 | 8.39 | 11.01 | 3 | 138.56 |  |
|  | 3 | 7.26 | 10.47 | 3.04 | 115.54 |  |
|  | 4 | 8.25 | 9.25 | 3.11 | 118.67 |  |
|  | 5 | 6.65 | 13.78 | 3.09 | 141.58 |  |
|  | 6 | 8.44 | 8.17 | 3.23 | 111.36 |  |
|  | 7 | 8.49 | 10.9 | 2.77 | 128.17 |  |
| **Post-surgery** | | | | | | |
| Day | Group 1 | Long side (mm) | Short side (mm) | Height (mm) | Volume (mm^3^) | Average with S.D. |
| 1 | 1 | 0 | 0 | 0 | 0.00 | 27.8 ± 37.10 |
|  | 2 | 5.53 | 10.22 | 3.89 | 109.92 |  |
|  | 3 | 5.57 | 3.75 | 1.22 | 12.74 |  |
|  | 4 | 3.64 | 5.44 | 2.59 | 25.64 |  |
|  | 5 | 3.96 | 5.17 | 1.99 | 20.37 |  |
|  | 6 | 3.32 | 4.56 | 1.34 | 10.14 |  |
|  | 7 | 5.65 | 4.3 | 1.3 | 15.79 |  |
| Day | Group 1 | Long side (mm) | Short side (mm) | Height (mm) | Volume (mm^3^) | Average with S.D. |
| 3 | 1 | 7.32 | 13.69 | 4.6 | 230.48 | 105.33 ± 91.95 |
|  | 2 | 4.04 | 4.62 | 2.05 | 19.13 |  |
|  | 3 | 2.84 | 3.9 | 1.4 | 7.75 |  |
|  | 4 | 7.39 | 4.67 | 2.32 | 40.03 |  |
|  | 5 | 8.25 | 7.39 | 3.52 | 107.30 |  |
|  | 6 | 7.81 | 7.98 | 3.52 | 109.69 |  |
|  | 7 | 9.21 | 14.76 | 3.28 | 222.94 |  |
| Day | Group 1 | Long side (mm) | Short side (mm) | Height (mm) | Volume (mm^3^) | Average with S.D. |
| 5 | 1 | 12.71 | 18.05 | 3.79 | 434.74 | 343.77 ± 216.10 |
|  | 2 | 10.36 | 12.39 | 3.84 | 246.45 |  |
|  | 3 | 10.29 | 20.88 | 6.59 | 707.95 |  |
|  | 4 | 7.71 | 20.02 | 3.92 | 302.53 |  |
|  | 5 | 8.13 | 13.54 | 5.14 | 282.91 |  |
|  | 6 | 5 | 7.17 | 2.82 | 50.55 |  |
|  | 7 | 12.44 | 15.06 | 4.07 | 381.25 |  |
| Day | Group 1 | Long side (mm) | Short side (mm) | Height (mm) | Volume (mm^3^) | Average with S.D. |
| 7 | 1 | 9.03 | 16.69 | 7.13 | 537.28 | 725.54 ± 352.13 |
|  | 2 | 10.71 | 10.96 | 4.11 | 241.22 |  |
|  | 3 | 18.18 | 17.76 | 4.9 | 791.05 |  |
|  | 4 | 13.36 | 12.98 | 6.41 | 555.79 |  |
|  | 5 | 14.79 | 23.71 | 7.79 | 1365.86 |  |
|  | 6 | 17.08 | 19.83 | 5.3 | 897.55 |  |
|  | 7 | 12.2 | 24.12 | 4.69 | 690.05 |  |

**Table S18**. Animal study records of tumor size in Group 2 (light only, 530 nm, 75 mW/cm^2^, 3 min). Tumor volume was calculated using the formula:

Volume = 0.5 × (long side) × (short side) × (height)

| Day | Group 2 | Long side (mm) | Short side (mm) | Height (mm) | Volume (mm^3^) | Average with S.D. |
| --- | --- | --- | --- | --- | --- | --- |
| 1 | 1 | 5.44 | 5.07 | 2.03 | 27.99 | 31.23 ± 21.27 |
|  | 2 | 4.62 | 4.34 | 1.58 | 15.84 |  |
|  | 3 | 5.59 | 4 | 2.15 | 24.04 |  |
|  | 4 | 4.12 | 5.45 | 1.77 | 19.87 |  |
|  | 5 | 9.71 | 5.92 | 2.38 | 68.41 |  |
| Day | Group 2 | Long side (mm) | Short side (mm) | Height (mm) | Volume (mm^3^) | Average with S.D. |
| 3 | 1 | 5.74 | 4.59 | 1.74 | 22.92 | 31.12 ± 21.64 |
|  | 2 | 4.7 | 6.39 | 1.83 | 27.48 |  |
|  | 3 | 5.1 | 4.05 | 1.58 | 16.32 |  |
|  | 4 | 4.25 | 7.37 | 1.26 | 19.73 |  |
|  | 5 | 7.03 | 8.16 | 2.41 | 69.12 |  |
| Day | Group 2 | Long side (mm) | Short side (mm) | Height (mm) | Volume (mm^3^) | Average with S.D. |
| 5 | 1 | 5.35 | 9.22 | 1.77 | 43.65 | 29.19 ± 11.16 |
|  | 2 | 5.75 | 4.2 | 1.02 | 12.32 |  |
|  | 3 | 4.79 | 5.68 | 2.21 | 30.06 |  |
|  | 4 | 5.71 | 4.51 | 2.24 | 28.84 |  |
|  | 5 | 4.93 | 8.41 | 1.5 | 31.10 |  |
| Day | Group 2 | Long side (mm) | Short side (mm) | Height (mm) | Volume (mm^3^) | Average with S.D. |
| 7 | 1 | 6.82 | 7.54 | 2.22 | 57.08 | 67.22 ± 56.83 |
|  | 2 | 5.85 | 7.3 | 2.33 | 49.75 |  |
|  | 3 | 4.5 | 7.3 | 1.44 | 23.65 |  |
|  | 4 | 7.23 | 4.68 | 2.32 | 39.25 |  |
|  | 5 | 8.34 | 10 | 3.99 | 166.38 |  |
| Day | Group 2 | Long side (mm) | Short side (mm) | Height (mm) | Volume (mm^3^) | Average with S.D. |
| 9 | 1 | 6.82 | 7.54 | 2.22 | 57.08 | 67.22 ± 56.83 |
|  | 2 | 5.85 | 7.3 | 2.33 | 49.75 |  |
|  | 3 | 4.5 | 7.3 | 1.44 | 23.65 |  |
|  | 4 | 7.23 | 4.68 | 2.32 | 39.25 |  |
|  | 5 | 8.34 | 10 | 3.99 | 166.38 |  |
| Day | Group 2 | Long side (mm) | Short side (mm) | Height (mm) | Volume (mm^3^) | Average with S.D. |
| 11 | 1 | 6.62 | 9.75 | 2.48 | 80.04 | 103.63 ± 55.04 |
|  | 2 | 4.92 | 6.85 | 2.67 | 44.99 |  |
|  | 3 | 9.01 | 10.87 | 3.78 | 185.10 |  |
|  | 4 | 7.9 | 11.58 | 2.87 | 131.28 |  |
|  | 5 | 6.82 | 7.84 | 2.87 | 76.73 |  |
| **Post-surgery** | | | | | | |
| Day | Group 2 | Long side (mm) | Short side (mm) | Height (mm) | Volume (mm^3^) | Average with S.D. |
| 1 | 1 | 8.7 | 5.74 | 3.24 | 80.90 | 58.51 ± 38.48 |
|  | 2 | 3.97 | 3.97 | 1.88 | 14.82 |  |
|  | 3 | 6.57 | 11.25 | 2.84 | 104.96 |  |
|  | 4 | 1.71 | 8.73 | 3.1 | 23.14 |  |
|  | 5 | 5.53 | 6.51 | 3.82 | 68.76 |  |
| Day | Group2 | Long side (mm) | Short side (mm) | Height (mm) | Volume (mm^3^) | Average with S.D. |
| 3 | 1 | 7.96 | 8.82 | 3.21 | 112.68 | 111.45 ± 60.52 |
|  | 2 | 5.15 | 5.15 | 2.21 | 29.31 |  |
|  | 3 | 8 | 11.03 | 4.1 | 180.89 |  |
|  | 4 | 8.22 | 7.75 | 2.46 | 78.36 |  |
|  | 5 | 7.65 | 13.2 | 3.09 | 156.01 |  |
| Day | Group2 | Long side (mm) | Short side (mm) | Height (mm) | Volume (mm^3^) | Average with S.D. |
| 5 | 1 | 9.06 | 10.05 | 3.37 | 153.42 | 201.59 ± 106.51 |
|  | 2 | 8.91 | 15.08 | 4.02 | 270.07 |  |
|  | 3 | 5.57 | 6.91 | 2.8 | 53.88 |  |
|  | 4 | 9.06 | 12.05 | 6.05 | 330.25 |  |
|  | 5 | 9.96 | 11.46 | 3.51 | 200.32 |  |
| Day | Group2 | Long side (mm) | Short side (mm) | Height (mm) | Volume (mm^3^) | Average with S.D. |
| 7 | 1 | 11.25 | 10.09 | 3.06 | 173.67 | 408.64 ± 230.43 |
|  | 2 | 13.31 | 13.74 | 6.71 | 613.56 |  |
|  | 3 | 13.35 | 17.94 | 4.44 | 531.69 |  |
|  | 4 | 11.52 | 17.85 | 5.65 | 580.91 |  |
|  | 5 | 9.41 | 8.19 | 3.72 | 143.35 |  |

**Table S19**. Animal study records of tumor size in Group 3 (1% agar gel). Tumor volume was calculated using the formula:

Volume = 0.5 × (long side) × (short side) × (height)

| Day | Group 3 | Long side (mm) | Short side (mm) | Height (mm) | Volume (mm^3^) | Average with S.D. |
| --- | --- | --- | --- | --- | --- | --- |
| 1 | 1 | 5.33 | 5.36 | 2.08 | 29.71 | 19.51 ± 6.03 |
|  | 2 | 3.07 | 6.2 | 1.5 | 14.28 |  |
|  | 3 | 5.26 | 4.07 | 1.55 | 16.59 |  |
|  | 4 | 4.87 | 4.19 | 1.94 | 19.79 |  |
|  | 5 | 5.83 | 4.33 | 1.36 | 17.17 |  |
| Day | Group 3 | Long side (mm) | Short side (mm) | Height (mm) | Volume (mm^3^) | Average with S.D. |
| 3 | 1 | 5.66 | 3.71 | 1.27 | 13.33 | 21.06 ± 6.71 |
|  | 2 | 5.36 | 4.68 | 1.56 | 19.57 |  |
|  | 3 | 5.34 | 5.22 | 1.63 | 22.72 |  |
|  | 4 | 4.33 | 5.58 | 1.51 | 18.24 |  |
|  | 5 | 5.38 | 6.15 | 1.9 | 31.43 |  |
| Day | Group 3 | Long side (mm) | Short side (mm) | Height (mm) | Volume (mm^3^) | Average with S.D. |
| 5 | 1 | 4.7 | 5.32 | 1.4 | 17.50 | 26.98 ± 8.61 |
|  | 2 | 7.31 | 4.76 | 1.77 | 30.79 |  |
|  | 3 | 5.54 | 4.36 | 1.65 | 19.93 |  |
|  | 4 | 5.5 | 5.18 | 1.95 | 27.78 |  |
|  | 5 | 5.86 | 5.65 | 2.35 | 38.90 |  |
| Day | Group 3 | Long side (mm) | Short side (mm) | Height (mm) | Volume (mm^3^) | Average with S.D. |
| 7 | 1 | 5.99 | 5.79 | 2.04 | 35.38 | 41.65 ± 17.05 |
|  | 2 | 7.43 | 4.78 | 2.4 | 42.62 |  |
|  | 3 | 6.81 | 6.81 | 2.82 | 65.39 |  |
|  | 4 | 7.02 | 3.87 | 1.36 | 18.47 |  |
|  | 5 | 4.71 | 7.94 | 2.48 | 46.37 |  |
| Day | Group3 | Long side (mm) | Short side (mm) | Height (mm) | Volume (mm^3^) | Average with S.D. |
| 9 | 1 | 6.71 | 5.89 | 1.53 | 30.23 | 60 ± 26.94 |
|  | 2 | 7.71 | 7.71 | 2.72 | 80.84 |  |
|  | 3 | 4.7 | 7.07 | 1.94 | 32.23 |  |
|  | 4 | 6.61 | 11.32 | 2.22 | 83.06 |  |
|  | 5 | 6.27 | 9.28 | 2.64 | 76.80 |  |
| Day | Group 3 | Long side (mm) | Short side (mm) | Height (mm) | Volume (mm^3^) | Average with S.D. |
| 11 | 1 | 8.81 | 11.47 | 3.02 | 152.59 | 95 ± 36.20 |
|  | 2 | 7.65 | 7.19 | 2.03 | 55.83 |  |
|  | 3 | 6.84 | 9.89 | 3.09 | 104.52 |  |
|  | 4 | 6.35 | 9.9 | 2.7 | 84.87 |  |
|  | 5 | 7.96 | 7.96 | 2.55 | 80.79 |  |
| **Post-surgery** | | | | | | |
| Day | Group 3 | Long side (mm) | Short side (mm) | Height (mm) | Volume (mm^3^) | Average with S.D. |
| 1 | 1 | 4.79 | 8.08 | 4.77 | 92.31 | 84.87 ± 6.25 |
|  | 2 | 5.83 | 7.96 | 3.59 | 83.30 |  |
|  | 3 | 5.73 | 7.96 | 3.38 | 77.08 |  |
|  | 4 | 4.87 | 11.8 | 2.84 | 81.60 |  |
|  | 5 | 6 | 8.78 | 3.42 | 90.08 |  |
| Day | Group 3 | Long side (mm) | Short side (mm) | Height (mm) | Volume (mm^3^) | Average with S.D. |
| 3 | 1 | 8.74 | 9.28 | 5.48 | 222.23 | 249.44 ± 143.94 |
|  | 2 | 8.61 | 13.77 | 3.76 | 222.89 |  |
|  | 3 | 16.99 | 15.53 | 3.58 | 472.30 |  |
|  | 4 | 8.81 | 13.12 | 4.47 | 258.34 |  |
|  | 5 | 5.39 | 7.3 | 3.63 | 71.41 |  |
| Day | Group 3 | Long side (mm) | Short side (mm) | Height (mm) | Volume (mm^3^) | Average with S.D. |
| 5 | 1 | 10.01 | 10.78 | 5.57 | 300.52 | 407.24 ± 158.38 |
|  | 2 | 20.07 | 15.1 | 4.37 | 662.18 |  |
|  | 3 | 10.26 | 16.58 | 5.12 | 435.48 |  |
|  | 4 | 7.89 | 16.99 | 3.84 | 257.38 |  |
|  | 5 | 9.64 | 13.71 | 5.76 | 380.63 |  |
| Day | Group 3 | Long side (mm) | Short side (mm) | Height (mm) | Volume (mm^3^) | Average with S.D. |
| 7 | 1 | 13.95 | 14.09 | 8.85 | 869.76 | 851.99 ± 141.51 |
|  | 2 | 21.63 | 19.06 | 4.85 | 999.75 |  |
|  | 3 | 14.47 | 17.28 | 7.57 | 946.41 |  |
|  | 4 | 11.42 | 20.32 | 5.47 | 634.67 |  |
|  | 5 | 14.39 | 16.47 | 6.83 | 809.37 |  |

**Table S20**. Animal study records of tumor size in Group 4 (1% agar gel + light, 530 nm, 75 mW/cm^2^, 3 min). Tumor volume was calculated using the formula:

Volume = 0.5 × (long side) × (short side) × (height)

| Day | Group 4 | Long side (mm) | Short side (mm) | Height (mm) | Volume (mm^3^) | Average with S.D. |
| --- | --- | --- | --- | --- | --- | --- |
| 1 | 1 | 5.98 | 3.15 | 2.24 | 21.10 | 19.36 ± 7.32 |
|  | 2 | 4.13 | 4.03 | 1.2 | 9.99 |  |
|  | 3 | 7.47 | 3.45 | 2.31 | 29.77 |  |
|  | 4 | 4.77 | 4.18 | 1.56 | 15.55 |  |
|  | 5 | 4.7 | 4.45 | 1.95 | 20.39 |  |
| Day | Group 4 | Long side (mm) | Short side (mm) | Height (mm) | Volume (mm^3^) | Average with S.D. |
| 3 | 1 | 6.73 | 5.73 | 2.03 | 39.14 | 23.94 ± 9.45 |
|  | 2 | 5.03 | 4.8 | 1.82 | 21.97 |  |
|  | 3 | 4.33 | 7.4 | 1.44 | 23.07 |  |
|  | 4 | 3.57 | 7.25 | 1.74 | 22.52 |  |
|  | 5 | 5.32 | 4.07 | 1.2 | 12.99 |  |
| Day | Group 4 | Long side (mm) | Short side (mm) | Height (mm) | Volume (mm^3^) | Average with S.D. |
| 5 | 1 | 4 | 8.57 | 2.22 | 38.05 | 31.91 ± 6.80 |
|  | 2 | 5.32 | 5.56 | 2.17 | 32.09 |  |
|  | 3 | 3.86 | 8.3 | 2.26 | 36.20 |  |
|  | 4 | 5.28 | 4.08 | 1.91 | 20.57 |  |
|  | 5 | 5.25 | 5.29 | 2.35 | 32.63 |  |
| Day | Group 4 | Long side (mm) | Short side (mm) | Height (mm) | Volume (mm^3^) | Average with S.D. |
| 7 | 1 | 5.09 | 6.43 | 2.38 | 38.95 | 29.97 ± 10.04 |
|  | 2 | 5.24 | 6.59 | 2.2 | 37.98 |  |
|  | 3 | 6.7 | 4.4 | 1.45 | 21.37 |  |
|  | 4 | 8.71 | 5.13 | 1.54 | 34.41 |  |
|  | 5 | 4.64 | 4.64 | 1.59 | 17.12 |  |
| Day | Group 4 | Long side (mm) | Short side (mm) | Height (mm) | Volume (mm^3^) | Average with S.D. |
| 9 | 1 | 7.79 | 7.52 | 1.93 | 56.53 | 47.6 ± 24.32 |
|  | 2 | 5.78 | 11.27 | 2.03 | 66.12 |  |
|  | 3 | 4.25 | 7.7 | 1.54 | 25.20 |  |
|  | 4 | 4.92 | 4.72 | 1.58 | 18.35 |  |
|  | 5 | 5.76 | 8.97 | 2.78 | 71.82 |  |
| Day | Group 4 | Long side (mm) | Short side (mm) | Height (mm) | Volume (mm^3^) | Average with S.D. |
| 11 | 1 | 9.51 | 8.4 | 2.31 | 92.27 | 126.13 ± 73.25 |
|  | 2 | 10.16 | 16.43 | 2.96 | 247.05 |  |
|  | 3 | 6.87 | 13.98 | 2.27 | 109.01 |  |
|  | 4 | 5.94 | 8.74 | 2.03 | 52.69 |  |
|  | 5 | 7.62 | 10.87 | 3.13 | 129.63 |  |
| **Post-surgery** | | | | | | |
| Day | Group 4 | Long side (mm) | Short side (mm) | Height (mm) | Volume (mm^3^) | Average with S.D. |
| 1 | 1 | 5.98 | 8.88 | 3.42 | 90.81 | 79.06 ± 57.75 |
|  | 2 | 5.05 | 4.48 | 1.72 | 19.46 |  |
|  | 3 | 4.05 | 6.4 | 1.47 | 19.05 |  |
|  | 4 | 7.75 | 7.88 | 4 | 122.14 |  |
|  | 5 | 7.65 | 7.77 | 4.84 | 143.85 |  |
| Day | Group 4 | Long side (mm) | Short side (mm) | Height (mm) | Volume (mm^3^) | Average with S.D. |
| 3 | 1 | 9.14 | 9.18 | 3.92 | 164.45 | 172.51 ± 90.72 |
|  | 2 | 8.02 | 9.37 | 4.06 | 152.55 |  |
|  | 3 | 8.84 | 8.92 | 5.5 | 216.85 |  |
|  | 4 | 11 | 12.69 | 4.12 | 287.56 |  |
|  | 5 | 5.97 | 5.47 | 2.52 | 41.15 |  |
| Day | Group 4 | Long side (mm) | Short side (mm) | Height (mm) | Volume (mm^3^) | Average with S.D. |
| 5 | 1 | 10.15 | 10.69 | 5.1 | 276.68 | 305.75 ± 140.14 |
|  | 2 | 9.24 | 10.47 | 4.68 | 226.38 |  |
|  | 3 | 7.15 | 9.65 | 3.85 | 132.82 |  |
|  | 4 | 14.66 | 15.01 | 4.34 | 477.50 |  |
|  | 5 | 10.34 | 15.48 | 5.19 | 415.36 |  |
| Day | Group 4 | Long side (mm) | Short side (mm) | Height (mm) | Volume (mm^3^) | Average with S.D. |
| 7 | 1 | 12.69 | 13.44 | 7.33 | 625.08 | 640.58 ± 266.376 |
|  | 2 | 11.54 | 12.25 | 6.26 | 442.47 |  |
|  | 3 | 10.09 | 10.69 | 5.99 | 323.05 |  |
|  | 4 | 13.92 | 17.12 | 7.31 | 871.02 |  |
|  | 5 | 16.36 | 17.2 | 6.69 | 941.26 |  |

**Table S21**. Animal study records of tumor size in Group 5 (NBSD-NOc-treated). Tumor volume was calculated using the formula:

Volume = 0.5 × (long side) × (short side) × (height)

| Day | Group 5 | Long side (mm) | Short side (mm) | Height (mm) | Volume (mm^3^) | Average with S.D. |
| --- | --- | --- | --- | --- | --- | --- |
| 1 | 1 | 3.05 | 4.97 | 1.02 | 7.73 | 15.41 ± 9.15 |
|  | 2 | 4.71 | 3.91 | 1.46 | 13.44 |  |
|  | 3 | 7.88 | 2.55 | 1.58 | 15.87 |  |
|  | 4 | 4.94 | 4.84 | 2.57 | 30.72 |  |
|  | 5 | 3.13 | 5.8 | 1.02 | 9.26 |  |
| Day | Group 5 | Long side (mm) | Short side (mm) | Height (mm) | Volume (mm^3^) | Average with S.D. |
| 3 | 1 | 6.57 | 3.31 | 1.33 | 14.46 | 17.96 ± 9.44 |
|  | 2 | 3.79 | 4.17 | 1.21 | 9.56 |  |
|  | 3 | 4.09 | 4.38 | 1.88 | 16.84 |  |
|  | 4 | 5.04 | 3.71 | 1.58 | 14.77 |  |
|  | 5 | 5.38 | 5.45 | 2.33 | 34.16 |  |
| Day | Group 5 | Long side (mm) | Short side (mm) | Height (mm) | Volume (mm^3^) | Average with S.D. |
| 5 | 1 | 4.73 | 5.69 | 2.1 | 28.26 | 24.89 ± 7.62 |
|  | 2 | 6.22 | 4.33 | 2.24 | 30.16 |  |
|  | 3 | 6.91 | 3.71 | 1.23 | 15.77 |  |
|  | 4 | 4.41 | 6.56 | 2.25 | 32.55 |  |
|  | 5 | 3.67 | 5.05 | 1.91 | 17.70 |  |
| Day | Group 5 | Long side (mm) | Short side (mm) | Height (mm) | Volume (mm^3^) | Average with S.D. |
| 7 | 1 | 5.46 | 6.47 | 1.99 | 35.15 | 32.1 ± 9.98 |
|  | 2 | 6.69 | 4.34 | 1.56 | 22.65 |  |
|  | 3 | 5.87 | 6.54 | 2.48 | 47.60 |  |
|  | 4 | 7.48 | 4.47 | 1.83 | 30.59 |  |
|  | 5 | 4.74 | 6.63 | 1.56 | 24.51 |  |
| Day | Group 5 | Long side (mm) | Short side (mm) | Height (mm) | Volume (mm^3^) | Average with S.D. |
| 9 | 1 | 6.7 | 7.1 | 1.82 | 43.29 | 55.31 ± 22.71 |
|  | 2 | 7.34 | 7.02 | 1.87 | 48.18 |  |
|  | 3 | 6.51 | 8.73 | 2.83 | 80.42 |  |
|  | 4 | 3.95 | 7.4 | 1.89 | 27.62 |  |
|  | 5 | 4.88 | 11.44 | 2.76 | 77.04 |  |
| Day | Group 5 | Long side (mm) | Short side (mm) | Height (mm) | Volume (mm^3^) | Average with S.D. |
| 11 | 1 | 6.71 | 11.19 | 3.23 | 121.26 | 109.78 ± 16.62 |
|  | 2 | 8.48 | 9.71 | 2.9 | 119.39 |  |
|  | 3 | 7.58 | 8.05 | 3.26 | 99.46 |  |
|  | 4 | 8.02 | 10.25 | 3 | 123.31 |  |
|  | 5 | 5.52 | 10.02 | 3.09 | 85.45 |  |
| **Post-surgery** | | | | | | |
| Day | Group 5 | Long side (mm) | Short side (mm) | Height (mm) | Volume (mm^3^) | Average with S.D. |
| 1 | 1 | 5.98 | 8.88 | 3.42 | 90.81 | 23.57 ± 32.06 |
|  | 2 | 5.05 | 4.48 | 1.72 | 19.46 |  |
|  | 3 | 4.05 | 6.4 | 1.47 | 19.05 |  |
|  | 4 | 7.75 | 7.88 | 4 | 122.14 |  |
|  | 5 | 7.65 | 7.77 | 4.84 | 143.85 |  |
| Day | Group 5 | Long side (mm) | Short side (mm) | Height (mm) | Volume (mm^3^) | Average with S.D. |
| 3 | 1 | 9.14 | 9.18 | 3.92 | 164.45 | 187.74 ± 66.43 |
|  | 2 | 8.02 | 9.37 | 4.06 | 152.55 |  |
|  | 3 | 8.84 | 8.92 | 5.5 | 216.85 |  |
|  | 4 | 11 | 12.69 | 4.12 | 287.56 |  |
|  | 5 | 5.97 | 5.47 | 2.52 | 41.15 |  |
| Day | Group 5 | Long side (mm) | Short side (mm) | Height (mm) | Volume (mm^3^) | Average with S.D. |
| 5 | 1 | 10.15 | 10.69 | 5.1 | 276.68 | 277.16 ± 106.70 |
|  | 2 | 9.24 | 10.47 | 4.68 | 226.38 |  |
|  | 3 | 7.15 | 9.65 | 3.85 | 132.82 |  |
|  | 4 | 14.66 | 15.01 | 4.34 | 477.50 |  |
|  | 5 | 10.34 | 15.48 | 5.19 | 415.36 |  |
| Day | Group 5 | Long side (mm) | Short side (mm) | Height (mm) | Volume (mm^3^) | Average with S.D. |
| 7 | 1 | 12.69 | 13.44 | 7.33 | 625.08 | 615.91 ± 234.66 |
|  | 2 | 11.54 | 12.25 | 6.26 | 442.47 |  |
|  | 3 | 10.09 | 10.69 | 5.99 | 323.05 |  |
|  | 4 | 13.92 | 17.12 | 7.31 | 871.02 |  |
|  | 5 | 16.36 | 17.2 | 6.69 | 941.26 |  |

**Table S22**. Animal study records of tumor size in Group 6 (NBSD-NOc-treated + light, 530 nm, 75 mW/cm^2^, 3 min). Tumor volume was calculated using the formula:

Volume = 0.5 × (long side) × (short side) × (height)

| Day | Group 6 | Long side (mm) | Short side (mm) | Height (mm) | Volume (mm^3^) | Average with S.D. |
| --- | --- | --- | --- | --- | --- | --- |
| 1 | 1 | 4.79 | 5.6 | 2.36 | 31.65 | 26.70 ± 11.31 |
|  | 2 | 8.78 | 4.58 | 2.45 | 49.26 |  |
|  | 3 | 4.91 | 4.51 | 2 | 22.14 |  |
|  | 4 | 6.45 | 3.93 | 2.15 | 27.25 |  |
|  | 5 | 6.19 | 3.97 | 1.86 | 22.85 |  |
|  | 6 | 7.01 | 3.55 | 1.48 | 18.42 |  |
|  | 7 | 3.86 | 4.33 | 1.83 | 15.29 |  |
| Day | Group 6 | Long side (mm) | Short side (mm) | Height (mm) | Volume (mm^3^) | Average with S.D. |
| 3 | 1 | 5.35 | 4.3 | 1.74 | 20.01 | 26.51 ± 11.33 |
|  | 2 | 4.96 | 9.89 | 1.79 | 43.90 |  |
|  | 3 | 3.96 | 7.32 | 1.87 | 27.10 |  |
|  | 4 | 5.56 | 4.34 | 1.8 | 21.72 |  |
|  | 5 | 5.2 | 3.96 | 1.16 | 11.94 |  |
|  | 6 | 5.41 | 6.45 | 1.97 | 34.37 |  |
| Day | Group 6 | Long side (mm) | Short side (mm) | Height (mm) | Volume (mm^3^) | Average with S.D. |
| 5 | 1 | 4.84 | 5.19 | 1.06 | 13.31 | 27.32 ± 15.27 |
|  | 2 | 3.73 | 7.59 | 1.87 | 26.47 |  |
|  | 3 | 4.24 | 7.07 | 1.88 | 28.18 |  |
|  | 4 | 4.88 | 5.22 | 1.3 | 16.56 |  |
|  | 5 | 5.93 | 4.18 | 1.87 | 23.18 |  |
|  | 6 | 6.58 | 6.81 | 2.51 | 56.24 |  |
| Day | Group 6 | Long side (mm) | Short side (mm) | Height (mm) | Volume (mm^3^) | Average with S.D. |
| 7 | 1 | 6.91 | 10.77 | 2.32 | 86.33 | 54.99 ± 26.80 |
|  | 2 | 6.96 | 8.16 | 2.49 | 70.71 |  |
|  | 3 | 4.54 | 6.41 | 1.13 | 16.44 |  |
|  | 4 | 6.72 | 9.65 | 2.34 | 75.87 |  |
|  | 5 | 6.21 | 6.1 | 2.01 | 38.07 |  |
|  | 6 | 6.04 | 6.02 | 2.34 | 42.54 |  |
| Day | Group 6 | Long side (mm) | Short side (mm) | Height (mm) | Volume (mm^3^) | Average with S.D. |
| 9 | 1 | 6.59 | 10.1 | 2.93 | 97.51 | 84.52 ± 45.40 |
|  | 2 | 7.37 | 12.63 | 2.93 | 136.37 |  |
|  | 3 | 8.17 | 9.46 | 3.37 | 130.23 |  |
|  | 4 | 5.09 | 4.83 | 1.91 | 23.48 |  |
|  | 5 | 7.39 | 4.92 | 2.5 | 45.45 |  |
|  | 6 | 6.7 | 8.12 | 2.72 | 73.99 |  |
| Day | Group 6 | Long side (mm) | Short side (mm) | Height (mm) | Volume (mm^3^) | Average with S.D. |
| 11 | 1 | 8.79 | 12.5 | 2.65 | 145.58 | 124.39 ± 47.35 |
|  | 2 | 8.89 | 14.05 | 2.84 | 177.36 |  |
|  | 3 | 5.84 | 8.29 | 2.09 | 50.59 |  |
|  | 4 | 8.06 | 10.17 | 3.16 | 129.51 |  |
|  | 5 | 7.48 | 13.31 | 3.15 | 156.81 |  |
|  | 6 | 8.78 | 8.01 | 2.46 | 86.50 |  |
| **Post-surgery** | | | | | | |
| Day | Group 6 | Long side (mm) | Short side (mm) | Height (mm) | Volume (mm^3^) | Average with S.D. |
| 1 | 1 | 2.94 | 4.38 | 1.02 | 6.57 | 34.69 ± 39.38 |
|  | 2 | 2.16 | 2.16 | 0.97 | 2.26 |  |
|  | 3 | N.D. | N.D. | N.D. | N.D. |  |
|  | 4 | 6.58 | 7.23 | 3.76 | 89.44 |  |
|  | 5 | 4.93 | 7.18 | 1.91 | 33.80 |  |
|  | 6 | 5.66 | 8.45 | 3.18 | 76.04 |  |
| Day | Group 6 | Long side (mm) | Short side (mm) | Height (mm) | Volume (mm^3^) | Average with S.D. |
| 3 | 1 | 2.03 | 2.55 | 0.74 | 1.92 | 49.46 ± 52.48 |
|  | 2 | 8.63 | 6.88 | 3.35 | 99.45 |  |
|  | 3 | 6.47 | 9.77 | 3.51 | 110.94 |  |
|  | 4 | 2.31 | 1.56 | 0.47 | 0.85 |  |
|  | 5 | 6.27 | 8.39 | 3 | 78.91 |  |
|  | 6 | 3.46 | 2.55 | 1.07 | 4.72 |  |
| Day | Group 6 | Long side (mm) | Short side (mm) | Height (mm) | Volume (mm^3^) | Average with S.D. |
| 5 | 1 | 7.85 | 10.96 | 3.76 | 161.75 | 91.07 ± 88.45 |
|  | 2 | 4.24 | 3.79 | 1.23 | 9.88 |  |
|  | 3 | 4.71 | 4.55 | 2.01 | 21.54 |  |
|  | 4 | 1.53 | 2.58 | 0.38 | 0.75 |  |
|  | 5 | 10.29 | 9.44 | 3.71 | 180.19 |  |
|  | 6 | 8.47 | 11.43 | 3.56 | 172.33 |  |
| Day | Group 6 | Long side (mm) | Short side (mm) | Height (mm) | Volume (mm^3^) | Average with S.D. |
| 7 | 1 | 3.02 | 3.36 | 1 | 5.07 | 210.78 ± 186.00 |
|  | 2 | 12.58 | 12.33 | 5.74 | 445.17 |  |
|  | 3 | 9.23 | 14.91 | 5.41 | 372.26 |  |
|  | 4 | 3.12 | 4.24 | 1.22 | 8.07 |  |
|  | 5 | 10 | 11.31 | 4.99 | 282.18 |  |
|  | 6 | 9.13 | 7.48 | 4.45 | 151.95 |  |

**Reference**

1. An, J. M.; Jeong, M.; Jung, J.; Yeo, S. G.; Park, S.; Kim, D., Next-Generation Femtech: Urine-Based Cervical Cancer Diagnosis Using a Fluorescent Biothiol Probe with Controlled Smiles Rearrangement. *ACS Applied Materials & Interfaces* **2024,** *16* (4), 4493-4504.

2. Daina, A.; Michielin, O.; Zoete, V., SwissADME: a free web tool to evaluate pharmacokinetics, drug-likeness and medicinal chemistry friendliness of small molecules. *Scientific Reports* **2017,** *7* (1), 42717.
